# Antennal transcriptome analysis and expression profiles of odorant binding proteins in *Eogystia hippophaecolus* (Lepidoptera: Cossidae)

**DOI:** 10.1186/s12864-016-3008-4

**Published:** 2016-08-18

**Authors:** Ping Hu, Jing Tao, Mingming Cui, Chenglong Gao, Pengfei Lu, Youqing Luo

**Affiliations:** Key Laboratory for Silviculture and Conservation of Ministry of Education, Beijing Forestry University, No.35 Tsinghua East Road, Haidian District, Beijing, 100083 People’s Republic of China

**Keywords:** *Eogystia hippophaecolus*, Olfactory proteins, Expression profile, Transcriptome

## Abstract

**Background:**

*Eogystia hippophaecolus* (Hua et al.) (Lepidoptera: Cossidae) is the major threat to seabuckthorn plantations in China. Specific and highly efficient artificial sex pheromone traps was developed and used to control it. However, the molecular basis for the pheromone recognition is not known. So we established the antennal transcriptome of *E. hippophaecolus* and characterized the expression profiles of odorant binding proteins. These results establish and improve the basis knowledge of the olfactory receptive system, furthermore provide a theoretical basis for the development of new pest control method.

**Results:**

We identified 29 transcripts encoding putative odorant-binding proteins (OBPs), 18 putative chemosensory proteins (CSPs), 63 odorant receptors (ORs), 13 gustatory receptors (GRs), 12 ionotropic receptors (IRs), and two sensory neuron membrane proteins (SNMPs). Based on phylogenetic analysis, we found one Orco and three pheromone receptors of *E. hippophaecolus* and found that *EhipGR13* detects sugar, EhipGR11 and EhipGR3 detect bitter. Nine OBPs expression profile indicated that most were the highest expression in antennae, consistent with functions of OBPs in binding and transporting odors during the antennal recognition process. *OBP6 was* external expressed in male genital-biased in, and this locus may be responsible for pheromone binding and recognition as well as mating. *OBP1* was the highest and biased expressed in the foot and may function as identification of host plant volatiles.

**Conclusions:**

One hundred thirty-seven chemosensory proteins were identified and the accurate functions and groups of part proteins were obtained by phylogenetic analysis. The most OBPs were antenna-biased expressed, which are involved in antennal recognition. However, few OBP was detected biased expression in the foot and external genitalia, and these loci may function in pheromone recognition, mating, and the recognition of plant volatiles.

**Electronic supplementary material:**

The online version of this article (doi:10.1186/s12864-016-3008-4) contains supplementary material, which is available to authorized users.

## Background

The olfactory recognition system plays a vital role in insect survival and reproduction owing to its roles of a number of essential processes, such as feeding, orientation, searching for hosts, mating, and oviposition. Studies of the molecular mechanisms of the olfactory system have provided new prospects for integrated pest management. The seabuckthorn carpenterworm *Eogystia hippophaecolus* (Hua et al.) (Lepidoptera: Cossidae) damages the seabuckthorn *Hippophae rhamnoides* L. (Rosales: Elaeagnaceae), which is widely distributed throughout northern and western China and prevents soil erosion and desertification [1]. Zhou reported that outbreaks of *E. hippophaecolus* can lead to greater than 70 % seabuckthorn mortality in plantations in the Inner Mongolia Autonomous Region [2]. In addition to damaging seabuckthorn, it has destroyed *Ulmus pumila* L. (Urticales: Ulmaceae) as well as two or three species in the family Rosaceae [3]. In 2003, 66,500 ha of seabuckthorn plantations were infested with the seabuckthorn carpenterworm [4], which is considered a major threat to seabuckthorn plantations in China [5].

A highly effective method to control the damaging larvae has not been developed owing to its complex ecological and life history traits; for example, the larvae bore into trunks and roots, complete one generation every 3–4 years, and exhibit 16 larval stages. By extracting female sex pheromone glands, identifying extracts, electroantennographic (EAG) analyses, and field trials, the sex pheromones of *E. hippophaecolus* female sex pheromone gland have been identified as (*Z*)-7-tetradecenyl acetate (Z7-14:Ac) and (*E*)-3-tetradecenyl acetate (E3-14:Ac) [6], and have been used to develop specific and efficient artificial sex pheromone traps [7]. Based on plant volatile identification and a Y-tube bioassay using seabuckthorn plants, (*Z*)-3-hexen-1-ol acetate is an effective compound for the detection of host location [8]; however, its application has not been examined in field trials. The sensilla of female *E. hippophaecolus* can be classified into seven subtypes on the antennae, i.e., chaetica, trichodea (two subtypes), basiconica (two subtypes), coeloconica, and Böhm’s bristles. In addition, chaetica, trichodea, and basiconic sensilla have been detected on the ovipositor [9]. Some studies have evaluated the molecular biological properties of *E. hippophaecolus*. For example, the *Hh-DH-PBAN* gene expression profile may be correlated with larval development and sex pheromone biosynthesis in female *E. hippophaecolus* [10]; by using Amplified Fragment Length Polymorphism markers, found the genetic structure of *E. hippophaecolus* be influenced by various confounding bio-geographical factors [11].

The olfactory recognition process includes perireceptor and receptor events. Perireceptor events of the chemosensory system involve odorant-binding proteins (OBPs) and chemosensory proteins (CSPs), and these are located in the lymph of sensilla at a high concentration. Binding proteins function by binding hydrophobic odorants (e.g., pheromones and plant volatiles) at the pores of sensilla and transporting them through the sensilla lymph to facilitate solubilization [12–15]. OBPs are soluble proteins characterized by a conserved pattern of six cysteines that form three disulfide bridges [16, 17]. One specific and important subfamily of OBPs is the group of pheromone binding proteins (PBPs) [18], which bind to pheromone compounds and participate in the pheromone recognition process. Many studies have shown that the expression profiles of most OBPs are antenna-biased, especially those of PBPs of *Sesamia nonagrioides* and *Helicoverpa assulta* [19, 20]; however, OBP expression is not restricted to olfactory tissues, and they may participate in other physiological processes [12, 21, 22]. CSPs have fewer cysteines (4) and are smaller than OBPs [12]; they bind to various odors [13, 23–26]. Interestingly, CSPs are expressed in almost all chemosensory organs and non-olfactory organs, indicating that their functions in odor transport are not restricted [27]. In addition, an analysis of nine Arthropoda genomes supported the birth-and-death model of OBP and CSP evolution [28].

Receptor events involve three receptor types, odorant receptors (ORs), ionotropic receptors (IRs), and gustatory receptors (GRs). These receptors are membrane proteins located in the outer dendrites of olfactory receptor neurons. Most food odorants are detected by members of the OR family [29]. IRs mediate olfactory responses to a variety of odors, including acids, aldehydes, and perhaps humidity [29]. GRs detect sugars, salts, amino acids, nucleotides, acidic pH conditions, a large variety of bitter compounds that activate bitter receptors, and CO_2_ [30]. In addition, functional studies have suggested that ORs and GRs act as ligand-gated ion channels in the detection of environmental chemicals and pheromones, and emerging data implicates particular family members in thermosensation and photoreception as well as in non-sensory roles [31]. Based on a phylogenetic analysis of nine species of Lepidoptera, there are several highly conserved clades of olfactory receptors, representing ancestral paralogous lineages with functional divergence in some lineages [32].

ORs involved in odorant reception and signal transduction or those that bind to volatile chemicals [33, 34] have been studied most extensively, especially in *Bombyx mori* and *Drosophila melanogaster*. The heptahelical domain in ORs is thought to function as a ligand-gated ion channel and/or to act metabotropically as a G protein-coupled receptor (GPCR) [35]. GPCRs contain seven putative transmembrane helices [36, 37], prompting a long-standing supposition that ORs act metabotropically [38–41]. Insect ORs dimerize with a highly conserved and universal co-receptor, *Orco* [42–45], to form odorant-gated ion channels [40, 46–48]. *OR1* and *OR3* of *Bombyx mori* have been examined in *Xenopus laevis* oocytes using the voltage-clamp technique [49]. Based on observed patterns of covariation in *OR* amino acid sequences in various species, functionally important residues are located in highly constrained *OR* regions and *OR* models exhibit a transmembrane domain packing arrangement that differs from that of canonical GPCRs [35]. Regarding the mechanisms underlying the high specificity of ORs, insect chemosensory systems are hybrids between evolved combinatorial coding and conserved dedicated circuits. The relative contribution of these two modes depends on perceived chemical space and OR repertoire size in different species. Combinatorial coding may be the dominant strategy in insects that are able to discriminate between many odorants and odor objects, or it may increase the perceptible odor space in species with small OR repertoires [50]. With respect to combinatorial coding, ORs exhibit both broad molecular receptivity and narrow olfactory tuning [51]; odorant intensity is determined by paralogous OR pairs, such as 42a and 42b of *D. melanogaster*, which detect low and high amounts of the same odorant ligand [52]. In addition, *N*,*N*-diethyl-meta-toluamide (DEET) acts as a molecular “confusant” that scrambles the insect odor code; this effect provides a compelling explanation for the broad-spectrum efficacy of DEET against multiple insect species [53]. In migratory locusts, RNA interference and behavioral assays have indicated that an OR-based signaling pathway, not an IR-based pathway, mediates the attraction of locusts to aggregation pheromones [54].

IRs are a conserved family of synaptic ligand-gated ion channels that evolved from ionotropic glutamate receptors (iGluRs) [55, 56], which are involved in both smell and taste in insects [57, 58]. Mutations in *IR84a*, *IR64a*, *IR8a*, and *IR25a* of *Drosophila* inhibit odor-evoked neuronal responses [59, 60]. However, the specificity of ligand recognition by IRs is unclear [57]. Some members of the IR superfamily are expressed in taste neurons. Intriguingly, *Drosophila* IR94b has been implicated in auditory system functions [61].

GRs have been studied most extensively in *D. melanogaster*, which can detect sugars, salts, amino acids, acidic pH conditions, bitter molecules, CO_2_, and nucleotides in taste receptors [30]. *Dmel*GR5a [62], *Dmel*GR64a [63], and *Dmel*GR64f [64] are involved in broad responses to sugars. *Dmel*GR43a [65] is a specific fructose receptor. *Dmel*GR33a [66] detects a wide range of bitter chemicals. *Dmel*GR21a and *Dmel*GR63a [67] detect CO_2_. In addition to specific tastes, *GRs* involved in the detection of glycerol, fatty acids, and hydrogen peroxide have also been identified in *D. melanogaster* [68–71].

In this study, we examined the antennal transcriptome of *E. hippophaecolus*, verified the accuracy of the transcriptome data, determined the expression profiles of OBPs, and evaluated the phylogenetic relationships between *E. hippophaecolus* OBPs and those of other species. We identified olfactory proteins that are the basis for the olfactory system. These data may help reveal olfactory receptive mechanisms and provide a theoretical basis for new pest control methods that impede the main olfactory recognition processes.

## Results

### Transcriptome sequencing and sequence assembly

In total, we generated 42,000,000 raw reads from a cDNA library of the male *E. hippophaecolus* antenna. The percentages of reads with q20 and q30quality scores were 97.10 and 92.12 %, respectively. The female antennal transcriptome yielded 36,000,000 raw reads, and the percentages of reads with q20 and q30 scores were 97.02 and 91.94 %, respectively. After trimming adapters, removing low-quality raw sequences using Trimmomatic (http://www.usadellab.org/cms/index.php?page=trimmomatic), blending male and female sequences, splicing and assembly (using Trinity), we obtained 17348 transcripts, with an N50 of 1418 bp, average length of 944 bp, and maximal length of 10,205 bp. The raw reads for *E. hippophaecolus* have been deposited in the NCBI SRA database under the GenBank accession number SRP070604.

### Homology analysis and gene ontology annotation

For 34.10 % of transcripts, we obtained matches to entries in the NCBI non-redundant (nr) protein database by blastx with an E-value cut-off value of 1e^−5^. We observed the most sequence matches to *B. mori* (47.00 %), followed by *Danaus plexippus* (18.82 %), *Tribolium castaneum* (2.62 %), *Papilio xuthus* (2.50 %), and *Acyrthosiphon pisum* (2.34 %). For the remaining 26.72 % of sequences, we detected matches with loci in other insects.

**Fig. 1:**
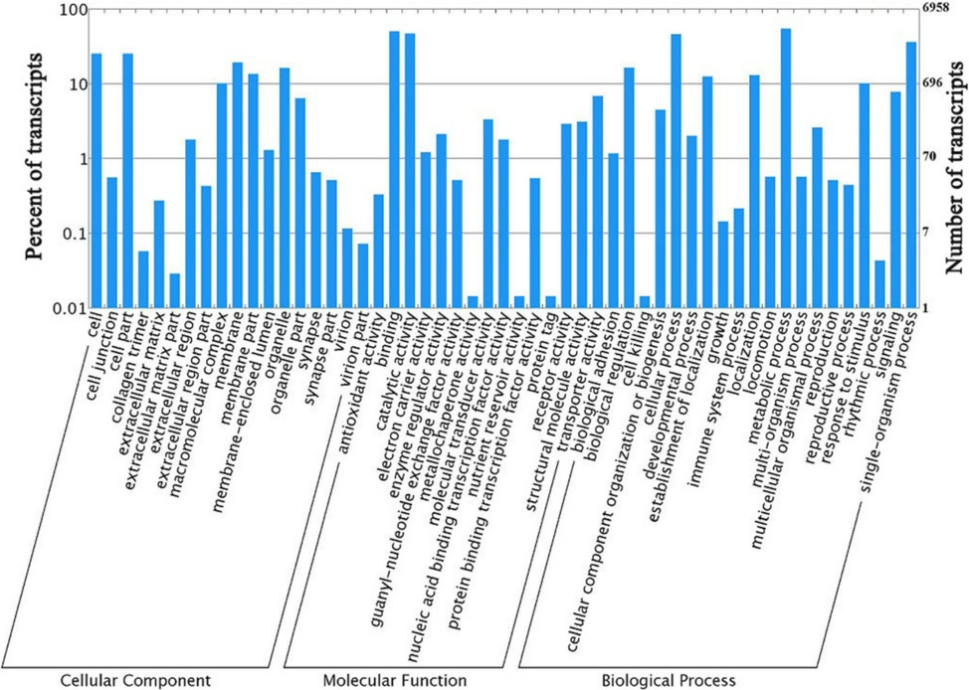
Gene Ontology (GO) results. GO analysis of 6958 genes in female and male *E. hippophaecolus* antenna transcriptome, according to their involvement in biological processes, cellular component and molecular function

We used gene ontology (GO) annotations to classify the 50,853 transcripts into functional groups using BLAST2GO which with P value calculated by hypergeometric distribution test and the E-value was less than 1 × 10^−5^. In the *E. hippophaecolus* transcriptome, molecular functions accounted for the majority of the GO annotations (81.65 %), and followed by biology process (65.78 %) and cellular component (41.25 %). In the molecular function category, the terms antioxidant activity, binding, and transporter activity were the most highly represented. In the biology process category, the terms cellular process, metabolic process, and single-organism process were most frequent. Cell, cell part, and membrane were the most abundant cellular component terms (Fig. 1).

## Nonreceptor olfactory gene families

### Odorant binding proteins

**Fig. 2:**
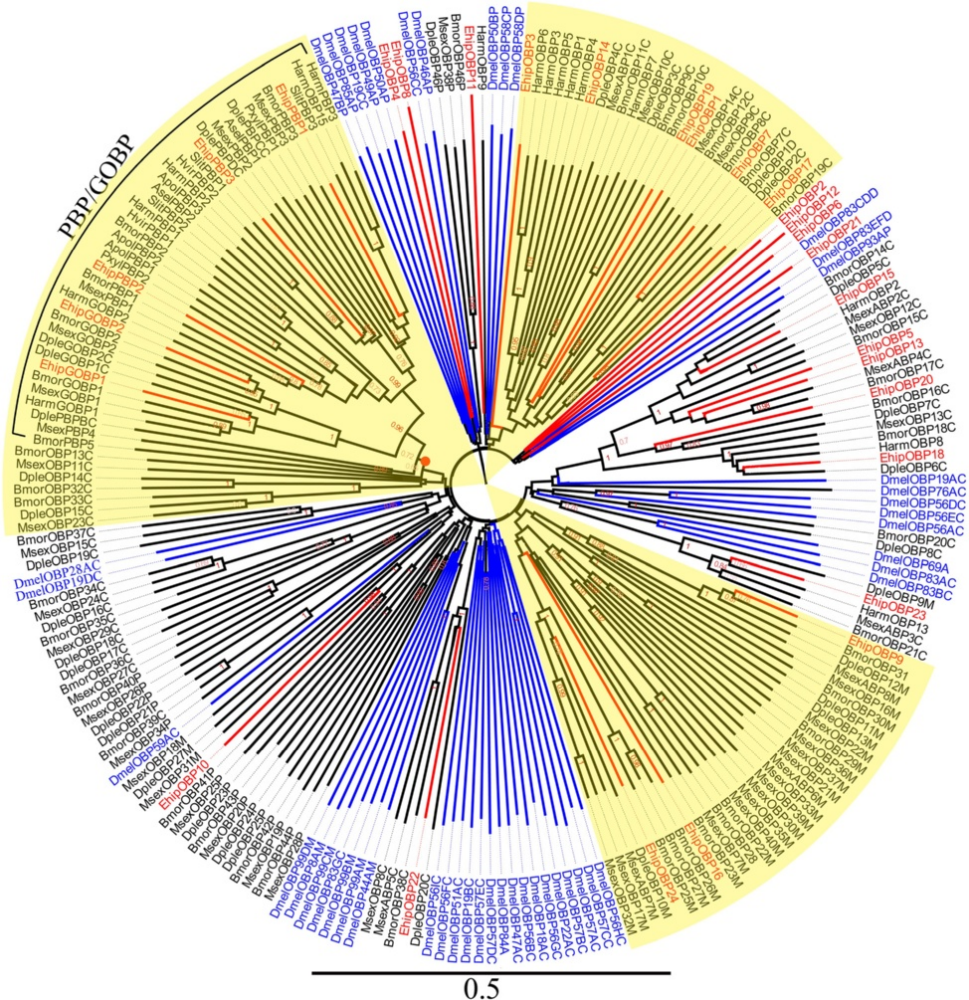
Neighbor-joining phylogenetic tree of odorant-binding proteins (OBPs). The NJ phylogenetic analysis of OBPs of *E. hippophaecolus* (*EhipOBP*, red) was performed with reference OBPs of *D. melanogaster* (*DmelOBP*, Diptera, blue) and OBPs of Lepidoptera species (black) [79]. The red circles refer to PBP/GOBP lineage. Pale yellow sector refer to the Lepidoptera-specific lineages. The stability of the nodes was assessed by bootstrap analysis with 1000 replications, and only bootstrap values ≥ 0.6 are shown at the corresponding nodes. The scale bar represents 05 substitutions per site

We identified 29 transcripts encoding putative OBPs in *E. hippophaecolus*. Seven were full-length genes with complete open reading frames (ORFs) of at least 400 bp and a signal peptide. Two were general odorant binding proteins (GOBPs), *EhipGOBP1* and *EhipGOBP2*; three (*EhipPBP1*, *EhipPBP2*, and *EhipPBP3*) were PBPs (Additional file 1: Table S1). Remarkably, using Blastx to identify gene homology, we observed the phenomenon in which more than one *E. hippophaecolus* gene exhibited a best match with the same species and gene sequence in the database. For example, we detected two unigenes (c31073_g1 and c19074_g1) that exhibited best matches with *Spodoptera exigua* AGP03457.1.The FPKM of female and male showed that *EhipOBP4*, *EhipGOBP1*, *EhipGOBP2*, *EhipPBP1* and *EhipPBP2* were the first five highest expressed in antenna. But *EhipPBP3* was much lower expressed in both female and male among PBPs. In the phylogenetic tree (Fig. 2), the distinct PBP/GOBP clade labelled with red circle included four PBP-specific lineages and a GOBP-specific lineage. The four PBP-specific lineages, the PBP-A sub-lineage (with 0.99 bootstrap support value) contained the most PBP genes, in which the EhipPBP2 was in this sub-lineage. The PBP-B sub-lineage contained *MsexPBP4* and *BmorPBP5*. The PBP-C sub-lineage contains *EhipPBP1* and *EhipPBP3* was in the PBP-D sub-lineage. Moreover, except PBP-B sub-lineage, the PBP-C sub-lineage with PBP-D sub-lineage, and the PBP-A sub-lineage were monophyletic. The GOBP1 and GOBP2 sub-lineages construct the GOBP-specific lineage with 1.0 bootstrap support. And consistently, *EhipGOBP1* and *EhipGOBP2* were clustered in GOBP1 and GOBP2 sub-lineages respectively. Besides, three Lepidopteran-specific lineages were labelled with green circle in Fig. 2.

### Chemosensory proteins

**Fig. 3:**
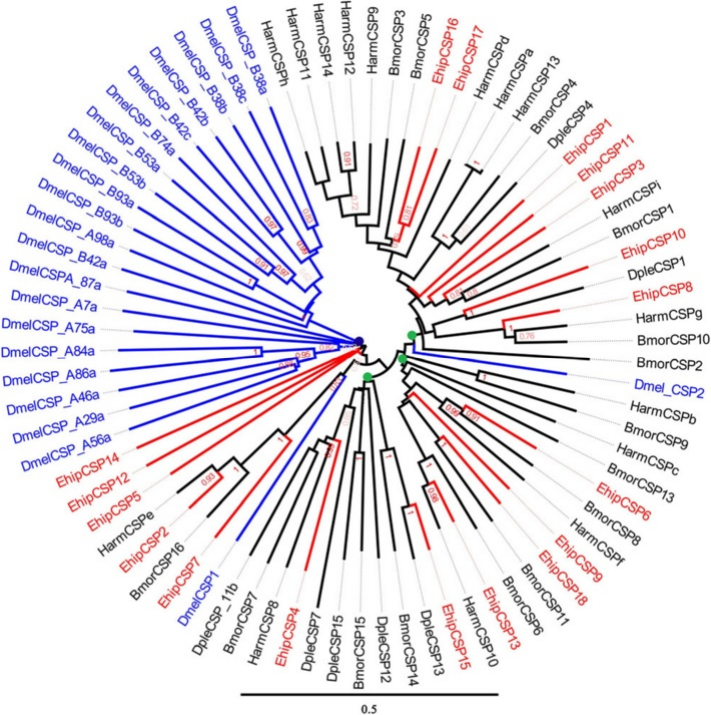
Neighbor-joining phylogenetic tree of chemosensory proteins (CSPs). The NJ phylogenetic analysis of CSPs of *E. hippophaecolus* (*EhipCSP*, red) was performed with reference CSPs of *D. melanogaster* (*DmelCSP*, Diptera, blue) and CSPs of Lepidoptera species (black). The stability of the nodes was assessed by bootstrap analysis with 1000 replications, and only bootstrap values ≥0.6 are shown at the corresponding nodes. The scale bar represents 0.5 substitutions per site

We identified 18 transcripts encoding putative CSPs. However, we did not obtain full-length genes encoding CSPs (Table 1). The FPKM of female and male showed that *EhipCSP14, EhipCSP5, EhipCSP6, EhipCSP8, EhipCSP10 and EhipCSP16* were the first six highest expressed in antenna. Based on a neighbor-joining tree of CSPs (Fig. 3), we found that *EhipCSP14*, *EhipCSP5*, and *EhipCSP12* were monophyletic with the big Dipteran (*D. melanogaster*) clade labelled with blue circle; *EhipCSP2, EhipCSP7, BmorCSP16 and HarmCSPe* were monophyletic with *DmelCSP1*; and *DmelCSP2* were monophyletic with the big Lepidoptera-specific lineage (labelled with green circle) contained with *EhipCSP16, EhipCSP17, EhipCSP1, EhipCSP11, EhipCSP3, EhipCSP10, and EhipCSP8*. Besides, the remaining two Lepidoptera-specific lineages (labelled with green circle) contain all other EhipCSPs.

**Table 1:** Best blastx hits for putative chemosensory proteins of *Eogystia hippophaecolus*

								Best Blast Match					
Number	Gene ID	Unigene length(bp)	ORF length(bp)	Compele ORF	Signal peptide	Male FPKM	Female FPKM	Name	ACC. Number	Speceies	Score	*E*-value	Identity
EhipCSP1	c18130_g1	555	378	Y	N	6.55	9.15	chemosensory protein 3	AGR39573.1	*Agrotis ipsilon*	171	1.00E-50	60 %
EhipCSP2	c24905_g1	745	324	Y	Y	6.25	12.57	chemosensory protein 5	AGR39575.1	*Agrotis ipsilon*	179	4.00E-53	85 %
EhipCSP3	c31175_g1	1159	372	Y	N	340.13	772.34	chemosensory protein 8	AGR39578.1	*Agrotis ipsilon*	162	7.00E-45	59 %
EhipCSP4	c33479_g1	511	369	Y	Y	185.69	151.70	chemosensory protein	AHC05672.1	*Chilo suppressalis*	138	4.00E-38	53 %
EhipCSP5	c20987_g1	2813	1572	Y	N	2248.90	2165.30	chemosensory protein	AHC05674.1	*Chilo suppressalis*	176	3.00E-47	68 %
EhipCSP6	c18929_g1	1710	375	Y	N	2480.61	1942.30	chemosensory protein 2	AGI37363.1	*Cnaphalocrocis medinalis*	182	4.00E-51	74 %
EhipCSP7	c13549_g1	851	336	Y	N	1.13	0.89	chemosensory protein	AIX97831.1	*Cnaphalocrocis medinalis*	153	8.00E-43	78 %
EhipCSP8	c7730_g1	1011	387	Y	Y	1454.17	1421.14	chemosensory protein 2	AHX37219.1	*Conogethes punctiferalis*	208	4.00E-63	76 %
EhipCSP9	c26669_g1	937	435	Y	N	2.43	1.80	chemosensory protein 5	AHX37227.1	*Conogethes punctiferalis*	138	2.00E-36	58 %
EhipCSP10	c10515_g1	2378	519	Y	N	1818.70	2000.39	chemosensory protein 4	AHX37226.1	*Conogethes punctiferalis*	228	3.00E-51	69 %
EhipCSP11	c18659_g1	902	372	Y	N	2.66	3.18	chemosensory protein	EHJ78408.1	*Danaus plexippus*	165	9.00E-47	60 %
EhipCSP12	c31194_g3	2895	309	Y	N	56.42	52.15	chemosensory protein	AIW65104.1	*Helicoverpa armigera*	205	6.00E-56	52 %
EhipCSP13	c22559_g1	660	366	Y	N	11.98	5.02	chemosensory protein 10	AFR92094.1	*Helicoverpa armigera*	220	2.00E-69	83 %
EhipCSP14	c3982_g1	691	396	Y	N	34811.82	41838.76	chemosensory protein	AAF71289.1	*Mamestra brassicae*	184	2.00E-55	69 %
EhipCSP15	c23394_g1	1087	378	Y	Y	7.71	11.47	chemosensory protein 13	BAG71921.1	*Papilio xuthus*	222	3.00E-68	84 %
EhipCSP16	c7482_g1	1082	384	Y	N	1048.02	1148.76	chemosensory protein CSP1	ABM67688.1	*Spodoptera exigua*	196	6.00E-58	72 %
EhipCSP17	c7703_g1	1039	384	Y	N	800.51	599.15	chemosensory protein CSP1	ABM67688.1	*Spodoptera exigua*	187	6.00E-55	70 %
EhipCSP18	c25262_g1	1684	366	Y	N	706.04	965.13	CSP6	AEX07267.1	*Helicoverpa armigera*	191	2.00E-55	73 %

### Sensory neuron membrane proteins

**Fig. 4:**
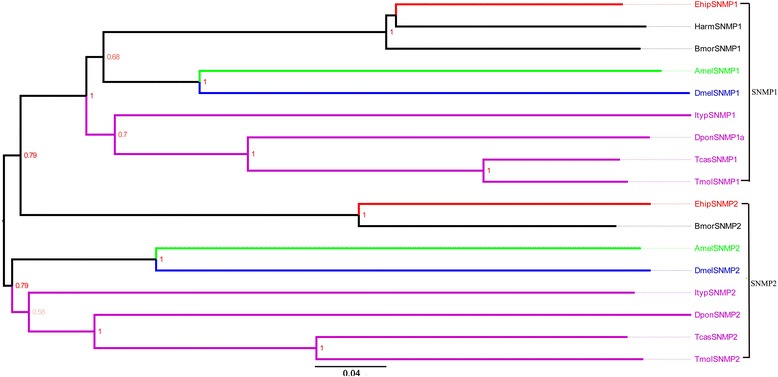
Neighbor-joining phylogenetic tree of sensory neuron membrane proteins (SNMPs). The NJ phylogenetic analysis of SNMPs of *E. hippophaecolus* (*EhipSNMP*, red) was performed with reference SNMPs of *D. ponderosae* (*DponSNMP*, purple), *I. typographus* (*ItypSNMP*, purple), *T. molitor* (Tmol*SNMP*, purple), *T. castaneum* (*TcasSNMP*, purple), *D. melanogaster* (*DmelSNMP*, Diptera, blue), *B. mori* (*BmorSNMP*, Lepidoptera, dark), *H.armigera* (*HarmSNMP*, dark),and *A. mellifera* (*AmelSNMP*, Hymenoptera, green). The stability of the nodes was assessed by bootstrap analysis with 1000 replications, and only bootstrap values ≥0.6 are shown at the corresponding nodes. The scale bar represents 0.04 substitutions per site

We identified two transcripts that encode putative sensory neuron membrane protein (SNMPs) with ORFs of approximately 1500 bp, indicating that both were nearly full-length genes. The FPKM of female and male showed that *EhipSNMP2* was much higher expressed than EhipSNMP1 in antenna (Additional file 1: Table S2). The E-values for Blastx searches were 0, indicating that they were homologous to known sequences in *Ostrinia nubilalis*. In the neighbor-joining tree of SNMPs (Fig. 4), we observed *EhipSNMP1* and *EhipSNMP2* in two Lepidoptera-specific lineages. SNMP1 and SNMP2 did not cluster in a monophyletic group respectively.

## Receptor encoding genes

### Odorant receptors

**Fig. 5:**
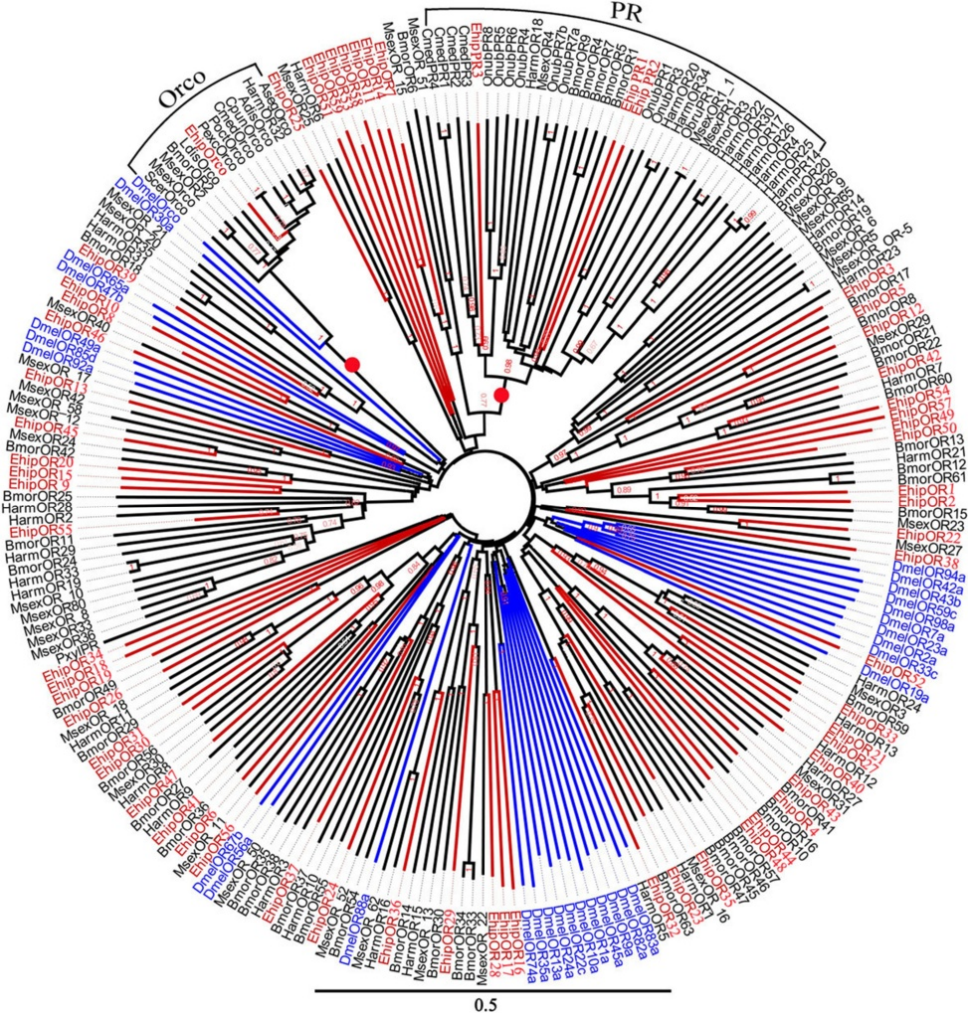
Neighbor-joining phylogenetic tree of odorant receptors (ORs). The NJ phylogenetic analysis of ORs of *E. hippophaecolus* (*EhipOR*, red) was performed with reference ORs of *D. melanogaster* (*DmelOR*, Diptera, blue) and ORs of Lepidoptera species (black). The red circles refer to Orco and PR lineage. The stability of the nodes was assessed by bootstrap analysis with 1000 replications, and only bootstrap values ≥0.6 are shown at the corresponding nodes. The scale bar represents 0.5 substitutions per site

We identified transcripts encoding 63 putative ORs. Among them, we detected 44 that likely represented full-length genes, encoding proteins of longer than 330 amino acids with complete ORFs. We detected multiple *E. hippophaecolus* ORs that were best matches with the same species and sequence (i.e., accession number). For example, the best match for *EhipOR1* and *EhipOR2* was *B. mori* NP_001091789.1, *EhipOR49* and *EhipOR50* exhibited best matches with *B. mori* NP_001166603.1, *EhipOR16* and *EhipOR17* matched *Danaus plexippus* EHJ75140.1, and *EhipOR30* and *EhipOR54* exhibited best matches with *Helicoverpa armigera* AIG51887.1. *EhipPR1, EhipOR16. EhipOrco and EhipOR58* were first four highest ORs expressed in antenna (male or female, or both) due to FPKM (Additional file 1: Table S3). In the phylogenetic tree (Fig. 5), the Orco lineage (labelled with red circle) with 1.0 bootstrap support value included *EhipOrco*, *DmelOrco* and Orcos of other Lepidoptera species. Besides, the pheromone receptor lineage (labelled with red circle) with 0.98 bootstrap support value contained all pheromone receptor (PR) of Lepidoptera species, except PxylPR, in which *EhipPR1, EhipPR2, and EhipPR3*were included (Fig. 4). Besides, all of the ORs of *E. hippophaecolus* clustered with Lepidopteran species in nine Lepidoptera-specific lineages, *EhipOrco*, *EhipOR52*, *EhipOR32*, *EhipOR13*, *EhipOR46*, *EhipOR8* and *EhipOR10* were excepted.

### Ionotropic receptors

**Fig. 6:**
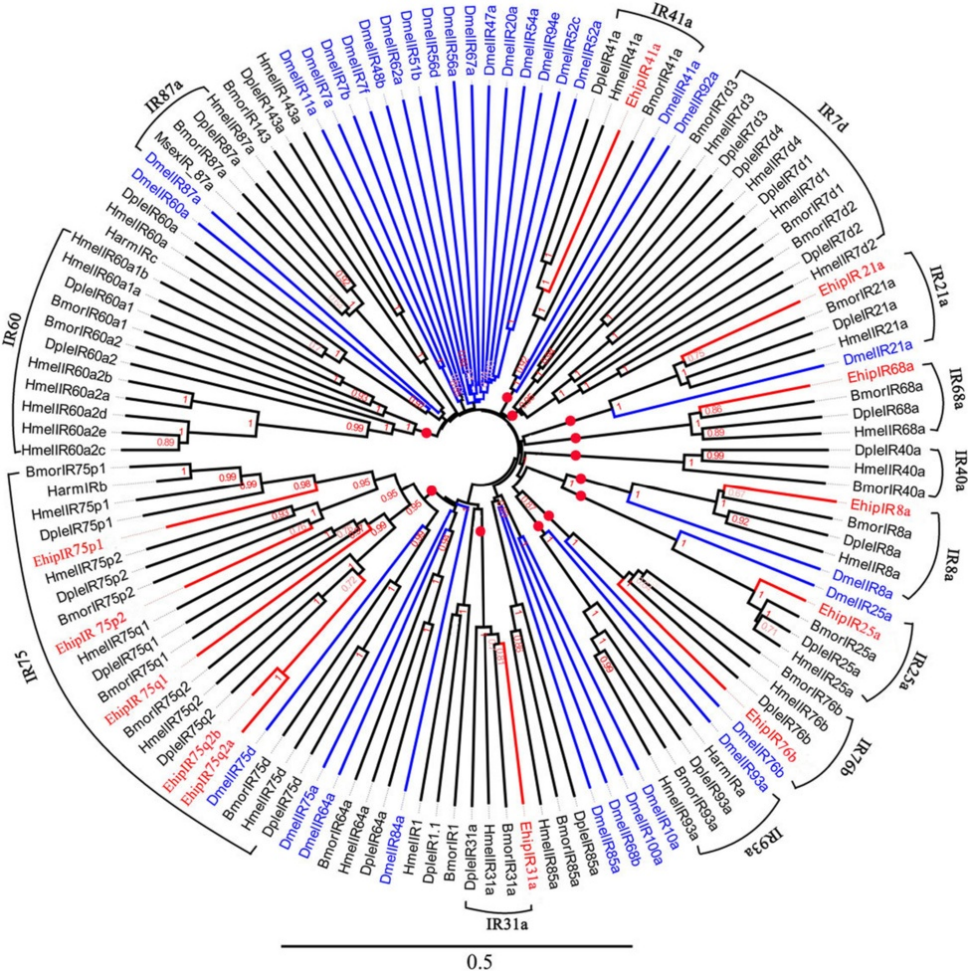
Neighbor-joining phylogenetic tree of ionotropic receptors (IRs). The NJ phylogenetic analysis of IRs of *E. hippophaecolus* (*EhipIR*, red) was performed with reference IRs of *H.armigera* (*HarmIR*, black), *D. melanogaster* (*DmelIR*, Diptera, blue) and IRs of other Lepidoptera species (black). The IRs groups labelled with red circle were reference of Van Schooten [82]. The stability of the nodes was assessed by bootstrap analysis with 1000 replications, and only bootstrap values ≥0.6 are shown at the corresponding nodes. The scale bar represents 0.5 substitutions per site

Twelve IRs, but not *EhipIR68a* which was most similar to ADR64682.1 of *Spodoptera littoralis*, were best matches with *Ostrinia furnacalis*. Among them, we detected eight that were likely to be full-length genes with complete bigger than 100 bp ORFs. *EhipIR76b* was the highest IRs expressed in antenna according to the FPKM of female and male (Additional file 1: Table S4). In the phylogenetic tree (Fig. 6), Most IRs were clustered as known group. For example, the IR41a group contained *EhipIR41a*, *DpleIR41a*, *HmelIR41a* and *BmorIR41a. EhipIR21a, EhipIR68, EhipIR8a, EhipIR25a, EhipIR76b*and *EhipIR31a* were in IR21a group, IR68 group, IR8a group, IR25a group, IR76b group and IR31a group which labelled with red circle respectively. The red circle labelled biggest group, IR75, were constructed by *EhipIR75p1, EhipIR75p1, EhipIR75p2, EhipIR75q2b, EhipIR75q2a, DmelIR75d* and IRs of other Lepidoptera species. All identified IRs of *E. hippophaecolus* were divided into eight IRs group. Besides, other known IRs group, IR60, IR93a, IR40a, IR7d, and IR87a were also clustered in the tree.

### Gustatory receptors

**Fig. 7:**
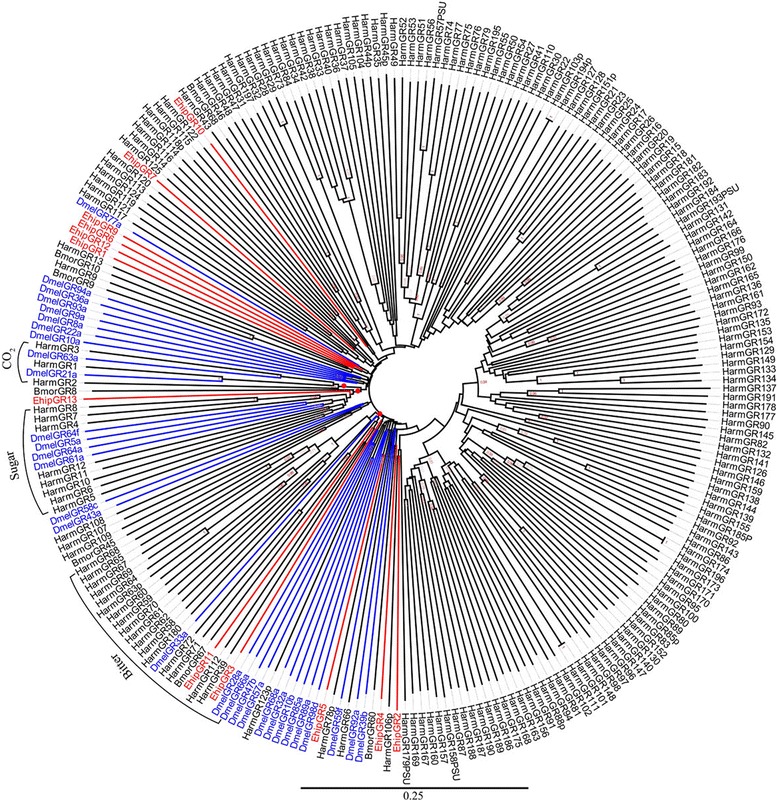
Neighbor-joining phylogenetic tree of gustatory receptors (GRs). The NJ phylogenetic analysis of GRs of *E. hippophaecolus* (*EhipGR*, red) was performed with reference GRs of *B.mori* (*BmorGR*, dark), *H.armigera* (*HarmGR*, dark) [85], *A. mellifera* (*AmelGR*, Hymenoptera, green), *T. castaneum* (*TcasGR*, Coleoptera, purple) and *D. melanogaster* (*DmelGR*, Diptera, blue). The GRs group labelled with red circle refers to detect CO_2_, sugar and bitter. The stability of the nodes was assessed by bootstrap analysis with 1000 replications, and only bootstrap values ≥0.6 are shown at the corresponding nodes. The scale bar represents 0.25 substitutions per site

We identified 13 transcripts encoding putative GRs. Only the ORFs of *EhipGR3* and *EhipGR13* were close to full-length genes with complete bigger than 1000 bp ORFs. Of the 13 GRs, we detected four (*EhipGR1*, *EhipGR7*, *EhipGR8*, and *EhipGR9*) that were best matches with *B. mori* DAA06391.1 and two (*EhipGR5* and *EhipGR10*) that were best matches with *B. mori* NP_001233217.1. *EhipGR11* was the highest GRs expressed in antenna according to the FPKM of female and male (Additional file 1: Table S5)*.* In the phylogenetic tree (Fig. 7), the sugar lineage contained *EhipGR13*. The bitter lineage were constructed by *EhipGR3, EhipGR11, DmelGR33a, DmelGR28a, DmelGR66a, BmorGR67* and bitter type 2 of *HarmGRs*.

## Fluorescence quantitative real-time PCR

**Fig. 8:**
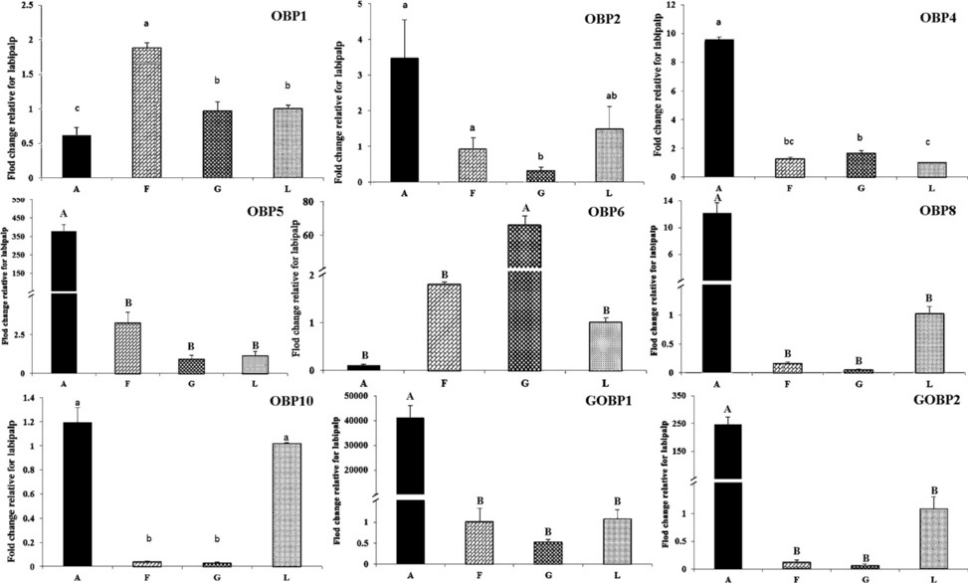
Odorant binding protein (OBPs) transcript levels of *E. hippophaecolus* in four tissues. A: antennae; F:foot; G: external genitals; L: labipalp. Actin was used as the reference gene to normalize target gene expression. The standard errors are represented by the error bars, different lowercase letters (a, b, c) above the bars denote significant differences at *p* < 0.05, and different capital letters (A, B) above the bars denote significant differences at *p* < 0.01

We verified OBP expression in antennae and characterized the expression profiles of nine OBPs in four chemosensory tissues of male (antennae, feet, external genitalia, and labial palps). For seven of the nine OBPs, we observed the highest expression levels in antennae (Fig. 8). Moreover, we detected very significantly higher expression levels of *OBP5*, G*OBP1*, G*OBP2*, and *OBP8* in antennae than in all other organs (*p* < 0.01), and we observed significantly antennal-biased expression for *OBP4* in all other organs (*p* < 0.05). We observed the highest *OBP2* and *OBP10* expression in antennae, and these expression levels were significantly higher than those in the feet and external genitalia, but were not significantly different than those in the labial palps. In labial palps, we observed that *OBP2* and *OBP10*were most highly expressed. Only *OBP1* was most highly expressed in the foot, and *OBP1* expression in the foot was significantly higher than that in other organs. We detected external genital-biased expression of *OBP6*. Noticeably, *OBP6* expression in external genitalia was extremely significantly higher than *OBP6*expression in all other organs. In summary, we observed antennal-biased expression of most *E. hippophaecolus* OBPs, but a few loci exhibited biased expression in the foot and external genitalia, such as *OBP1* and *OBP6*, respectively. We did not observe any OBPs in *E. hippophaecolus* that exhibited biased expression in labial palps.

## Discussion

Many olfactory proteins in Lepidoptera defoliators have prophylactic and treatment applications. However, the olfactory proteins in lepidopteran borer species, especially those belonging to Cossidae, have not been studied to date. To better understand the vital role of olfactory proteins in borer moths, we investigated chemosensory proteins in the antennal transcriptome of *E. hippophaecolus* and characterized the expression profiles of these loci by fluorescence quantitative real-time PCR. Our results provide direct molecular evidence for a role of olfactory proteins in chemosensory reception, establish a foundation for understanding the molecular mechanisms of olfactory recognition, and have applications to borer integrated pest management.

OBPs are considered the first gate in the odorant recognition process, especially for hydrophobic odors; they bind and transport odors, including pheromones and plant volatiles, across the lymph in the sensillum [17]. We identified 29 putative OBPs, of which we studied the expression of nine in antennae and other chemosensory tissues. Remarkably, most OBPs exhibited biased expression in antennae, suggesting a vital role of OBPs in antennal recognition processes. These results were consistent with previous observations in *Helicoverpa assulta* [20], *Chilo suppressalis* [72], *Loxostege sticticalis* [73], *Sesamia inferens* [74], and *Agrotis ipsilon* [75]. And found except *EhipOBP6* and *EhipOBP8*, other four antenna-biased expressed OBPs were with obviously high FPKM consistently, which further supported the antenna-biased expression characteristic of OBPs. Some OBPs exhibited biased expression in non-antennal chemosensory tissues; we observed biased *EhipOBP1* expression in the foot, and this may be related to the identification of host plant volatiles. We also observed extremely biased *EhipOBP6* expression in male external genitalia, providing direct molecular evidence that the locus is responsible for pheromone binding and involved in mate recognition. We did not detect *E. hippophaecolus* OBPs that exhibited biased expression in labial palps; considering the function of labial palps, we speculated that gustatory molecular binding proteins and contact sensillum-biased chemosensory proteins are highly expressed in labial palps. The expression profiles of other OBPs in Lepidoptera species have been investigated at various stages, in both sexes, and in chemosensory and non-chemosensory tissues [74, 76, 77]. CSPs are regarded as a subfamily of OBPs. OBPs and CSPs exhibited different expression profiles; OBP expression was antennal-biased, but CSPs have no obvious expression bias [74]. The expression of CSPs in *Anoplophora glabripennis* is not antennal-biased, but is high in other chemosensory tissues, such as the maxillary palps and propodeum [78]. In brief, these results indicated that OBPs mainly participate in antennal olfactory recognition, but CSPs are involved in olfactory recognition in many chemosensory organs. In the dendrogram of OBPs, the four PBP lineages and GOBP lineage comprised the PBP/GOBP complex, which support Vogt’s result of the monophyletic of PBP/GOBP and PBP with more dynamic evolution than GOBP [79]. Besides, 18 candidate chemosensory proteins were identified. Based on the phylogenetic analysis, Almost all CSPs of Diptera formed a taxon-specific clade, which diversification according to the divergence of insect orders has also been observed in *Mamestra brassicae* [26]. The SNMPs are conserved throughout holometabolous insects [80, 81], however SNMP1 and SNMP2 did not cluster in a monophyletic group respectively, which indicated the differentiation of SNMPs among classification.

ORs connect binding proteins with olfactory sensory neurons and conduct olfactory signal transduction. In female and male antennae, we detected 63 ORs. In the neighbor-joining tree, we observed *EhipOrco* in the Orco lineage, which suggests that we found the *E. hippophaecolus* Ocro. And three *EhipPRs* were in the pheromone receptor lineage with high bootstrap support value, which indicates that *EhipPR1, EhipPR2* and *EhipPR3* are pheromone receptor of *E. hippophaecolus.* But the relationship between three pheromone receptors and three pheromone binding protein need to study further.

We identified 12 IRs in the antennal transcriptome. According to the genome-wide analysis of ionotropic receptors in *Heliconius* butterflies, the IRs group was added and improved [82]. With reference of this study, we found seven IR groups in *E. hippophaecolus,* which were *EhipIR41a, EhipIR21a, EhipIR68, EhipIR8a, EhipIR25a, EhipIR76b,* and *EhipIR31a*. Besides, *EhipIR75p1, EhipIR75p1, EhipIR75p2, EhipIR75q2b* and *EhipIR75q2a* were IR75 of *E. hippophaecolus,* which were new identified IRs diversity group and consistent with corresponding functions. Notably, transcripts putatively encoding *IR8a* and *IR25a* which are thought to function as IR co-receptors [55, 56] were also found in *E. hippophaecolus*.

We also detected 13 GRs in the antennal transcriptome, which provides important sequence information. GRs typically function in sensing sugar, CO_2_ and bitter molecules [30]. In the phylogenetic tree, GRs involved in the detection of sugar, CO2 and bitter molecules clustered in a group. The sugar receptor lineages were included *BmorGR8* and *BmorGR9*, consistent with the functions of them [83, 84] and include *HarmGR8, HarmGR7, HarmGR4, HarmGR12, HarmGR10, HarmGR6* and *HarmGR5*, consistent with their functions [85]. *EhipGR13* belonged to the sugar-sensing lineage, indicating that it is function in sugar detection. As for bitter sensing, with reference of genome analysis of gustatory receptor in *H. armigera*, the type 2 bitter GRs with long intronless and clustered together in the phylogenetic tree [85] was also clustered in tree and formed bitter lineage that contain *EhipGR11* and *EhipGR3.* Known other more EhipGRs’ function should study and research systematically and deeply.

The FPKM of ORs, GRs and IRs were much less than binding proteins (OBPs and CSPs) integrally, which indicated that most olfactory membrane proteins genes were low expressed in antenna. Besides, result of two or more unigenes matched to the same gene was common in *E. hippophaecolus* transcriptome data and others. The reason may as follows. Firstly was due to the insufficient elaborate gene diversity and gene type in nr database. Second, this unigens contiained fragments of a same gene, which couldn't be properly assembled. Third, this unigens were paralogs with the same gene.

## Conclusion

We reported the *E. hippophaecolus* antennal transcriptome, which is the first analysis of olfactory proteins in a Cossidae species. We identified 137 olfactory genes that provide a foundation for studies of the olfactory recognition process and the olfactory system. For instance, the PBP/GOBP complex supported the monophyletic between them *Orco* and *three* pheromone receptors were found in *E. hippophaecolus*; *EhipGR13* detects sugar, and *EhipGR11* and *EhipGR3* detect bitter. Additionally, we verified the expression of nine OBPs in antennae and confirmed the accuracy of the antennal transcriptome data. And we observed antennal-biased expression for nearly all nine OBPs, and observed extremely antennal-biased expression of *OBP4*, *OBP5*, *OBP8,* G*OBP1*, and G*OBP2* demonstrating that OBPs primarily function in the antennal recognition process. Moreover, a few OBPs exhibited biased expression in other chemosensory tissues and may a component of olfactory binding that target pheromones and volatiles in external genitals and feet.

## Methods

### Ethics statement

The seabuckthorn carpenterworm *Eogystia hippophaecolus*(Lepidoptera: Cossidae) is a common forestry pest in China, which collections were made with the direct permission of Jianping forestry bureau. It’s not included in the “List of Endangered and Protected Animals in China”. All operations were performed according to ethical guidelines in order to minimize pain and discomfort to the insects.

### Insect and tissue collection

*E. hippophaecolus* were collected from damaging seabuckthorn forest by light and sex pheromone trip during middle of June to end of July 2014 to 2015 in Jianping, Liaoning, China. Antennae, foot (propodeums, mesopodiums, metapedes), external genitals, labipalp from males and females were excised and stored in RNAlater (Ambion, Austin, TX, USA). Then all samples were taken back indoor and stored at -80 °C.

### cDNA library construction and Illumina sequencing

Total RNA was extracted from two female and two male antennae using TRIzol reagent (Ambion) and the RNeasy Plus Mini Kit (No. 74134; Qiagen, Hilden, Germany) following the manufacturer’s instructions. RNA quantity was detected using the NanoDrop 8000 (Thermo, Waltham, MA, USA). RNA of male and female antennae was used to construct the cDNA library respectively. cDNA library construction and Illumina sequencing of samples were performed at CapitalBio Corporation (Beijing, China). mRNA samples were purified and fragmented using the TruSeq RNA Sample Preparation Kit v2-Set A (No. RS-122-2001; Illumina, San Diego, CA, USA). Random hexamer primers were used to synthesize the first-strand cDNA, followed by synthesis of the second-strand cDNA using buffer, dNTPs, RNase H, and DNA polymerase I at 16 °C for 1 h. After end repair, A-tailing, and the ligation of adaptors, the products were amplified by PCR and quantified precisely using the Qubit DNA Br Assay Kit (Q10211; Invitrogen, Carlsbad, CA, USA). They were then purified using the MinElute Gel Extraction Kit (Qiagen, Cat No. 28604) to obtain a cDNA library. The cDNA library was sequenced on the HiSeq2500 platform.

### Assembly and functional annotation

All raw reads were processed to remove low-quality and adaptor sequences by Trimmomatic (http://www.usadellab.org/cms/index.php?page=trimmomatic) . Clean reads assembly was carried out with the short-read assembly program Trinity (Version: r2014-04-13) with the default parameters after combined the male and female clean reads. The largest alternative splicing variants in the Trinity results were called unigenes. The annotation of unigenes was performed by NCBI BLASTx searches against the Nr protein database, with an E-value threshold of 1e-5. The blast results were then imported into the Blast2GO pipeline for GO annotation. The longest ORF for each unigene was determined by the NCBI ORF Finder tool (http://www.ncbi.nlm.nih.gov/gorf/gorf.html). Expression levels were expressed in terms of FPKM values (fragments per kilobase per million reads) [86], which was calculated by RSEM (RNA-Seq by Expectation-Maximization) (Version: v1.2.6) with default parameters [87].

### Identification of chemosensory genes

With BLASTx, the available sequences of OBP, CSP, OR, GR, IR, and SNMP proteins from insecta species were used as queries to identify candidate unigenes involved in olfaction in *E. hippophaecolus* from Nr database. All candidate OBPs, CSPs, ORs, GRs, IRs, and SNMPs were manually checked by tBLASTn in NCBI online assessing the BLASTx results. The Nucleic acid sequences encoded by all chemosensory genes that were identified from the *E. hippophaecolus* antennal transcriptome are listed in Additional file 2.

### Sequence and phylogenetic analysis

The candidate OBPs and PBPs were searched for the presence of N-terminal signal peptides using SignalP4.0 (http://www.cbs.dtu.dk/services/SignalP/). Amino acid sequence alignment was performed using Muscle method implemented in Mega v6.0 software package [88]. The phylogenetic tree was constructed using the neighbor-joining (NJ) method [89] with P-distances model and a pairwise deletion of gaps performed in Mega v6.0 software package. The reliability of the tree structure and node support was evaluated by bootstrap analysis with 1000 replicates. To obtain a tree with higher support, sequences of less than 122 amino acids (binding proteins)/300 amino acids (membrane protein) were removed, except *E. hippophaecolus* olfactory proteins. The phylogenetic trees were colored and arranged in FigTree (Version 1.4.2). The phylogenetic analyses of *OBPs* was based on 24 amino sequences of OBPs, 2 GOBPs and 3 PBPs of *E. hippophaecolus,* 13 OBPs, 1GOBPs, and 3PBPs of *Helicoverpa armigera* and with reference of Vogt used OBPs of *Drosophila melanogaster*, *Bombyx mori, Manduca sexta, Danaus plexippus*, *Heliconius Melpomene, Spodoptera littoralis, Heliothis virescens, Antheraea Polyphemus, A. pernyi, Ascotis selenaria, Ectropis obliqua and Plutella xylostella* [79], and the last one capital letter of OBP name, C,P,M,D refer to classic, plus-C, minus-C and duplex OBP respectively. CSPs tree was based on 18 of *E. hippophaecolus,* 15 of *B. mori,* 16 of *H. armigera,* and 22 of *D. melanogaster.* ORs tree were based on 63 ORs of *E. hippophaecolus,* 47 of *B. mori,* 35 of *H. armigera,* 40 of *M. sexta,* 30 of *D. melanogaster,* eight Orco and 16 PR. IRs tree was based on 13 IRs of *E. hippophaecolus,* 3 IRs of *H. armigera,* and 35 of *D. melanogaster* and with reference of van Schooten used IRs of *M. sexta, B. mori, D. plexippus*, and *H. Melpomene* [82]. GRs tree was based on 13 GRs of *E. hippophaecolus*, 7 of *B. mori,* 30 of *D. melanogaster* and with reference of XU used GRs of *H. armigera* [85] SNMPs tree was based on 2 amino sequences of SNMPs of *E. hippophaecolus*, 2 of *B. mori,* 1 of *H. armigera, 2* of *A. mellifera, 2* of *T. castaneum, 2 of D. ponderosae*, 2 of *I. typographus*, 2 of *T. molitor* and 2 of *D. melanogaster* in SNMPs tree. Accession number of chemosensory proteins used in tree without reference was listed in Additional file 3.

### Expression analysis by fluorescence quantitative real-time PCR

Fluorescence quantitative real-time PCR was performed to verify the expression of candidate chemosensory genes. Six chemosensory tissues, antennae, foots (including the propodeum, mesopodium, and metapedes), external genitals, labial palps were collected from ten male *E. hippophaecolus* and one total RNA was extracted following the methods described above. The propodeum, mesopodium, and metapedes RNA were accounted for one third of all foot RNA. NanoDrop2008 and agarose gel electrophoresis examined density and quality of RNA. cDNA was synthesized from total RNA using the PrimeScriptRT Reagent Kit with gDNA Eraser to remove gDNA(No. RR047A; TaKaRa, Shiga, Japan). Gene-specific primers were designed using Primer3 (http://bioinfo.ut.ee/primer3-0.4.0/) (Additional file 4). Four actin genes were identified and selected from the *E. hippophaecolus* antennal transcriptome, then used Normfinder and GeNorm to evaluate, and selected the actin gene with minimum M value(GeNorm) and Stability value(Normfinder) as reference gene for qPCR(Additional file 5: Table S1 and Figure S1). A PCR analysis was conducted using the Bio-Rad CFX96 PCR System (Hercules, CA, USA). SYBRPremix ExTaq™ II (No. RR820A; TaKaRa) was used for the PCR reaction under a three-step amplification. Each PCR reaction was conducted in a 25-ml reaction mixturecontaining12.5 μl of SYBR Premix Ex Taq II, 1 ml of each primer (10 mM), 2 μl of sample cDNA (2.5 ng of RNA), and 8.5 μl of dH2O (sterile distilled water). The RT-qPCR cycling parameters were as follows: 95 °C for 30 s, followed by 40 cycles of 95 °C for 5 s, 60 °C for 30 s, and 65 to 95 °C in increments of 0.5 °C for 5 s to generate the melting curves. To examine reproducibility, each qPCR reaction for each tissue was performed in three biological replicates and three technical replicates, in which each biological replication was with ten individuals, each biological replication with three technical replicates. Negative controls without either template were included in each experiment. Bio-Rad CFX Manager (version 3.1.1517.0823) was used to normalize expression based on ΔΔCq values, with labial palps in analyze mode as control samples, and the 2^-ΔΔCT^ method was used (the amplification efficiency for 9 genes was equal to 100 %) [90]. Before comparative analyses, examined the normal distribution and equal variances test, and all taken logarithm data followed normal distribution and with equal variances (Additional file 5: Figure S2-S4). So the comparative analyses for every gene among six tissue types were assessed by a one-way nested analysis of variance (ANOVA), followed by Tukey’s honestly significance difference (HSD) tests implemented in SPSS Statistics 18.0. Values are presented as means ± SE.

## Additional files

Additional file 1: Best blastx hits for putative odorant binding proteins (OBPs), sensory neuron membrane proteins(SNMPs), odorant receptors(ORs), ionotropic receptors(IRs), and gustatory receptors(GRs) of *Eogystia hippophaecolus.* Table S1, Table S2, Table S3, Table S4. **Table S1.** Sequence information and best blaxts match information of putative odorant binding proteins (OBPs). **Table S2.** Sequence information and best blaxts match information of sensory neuron membrane proteins (SNMPs). **Table S3.** Sequence information and best blaxts match information of Odorant receptors (ORs). **Table S4.** Sequence information and best blaxts match information of ionotropic receptors (IRs). **Table S5.** Sequence information and best blaxts match information of gustatory receptors (GRs). (PDF 233 kb) Additional file 2: Nucleic acid sequences of all candidate chemosensory proteins identified in *Eogystia hippophaecolus* transcriptome. (PDF 586 kb) Additional file 3: The protein names and gene accession numbers were used in phylogenetic trees. (PDF 182 kb) Additional file 4: Primers were designed for fluorescence quantitative real-time PCR. (PDF 90 kb) Additional file 5: Reference gene and statistics analysis of CQ in fluorescence quantitative real-time PCR. **Table S1.** Normfinder result of four reference genes. **Figure S1.** M value of gNorm result of four reference genes. **Figure S2.** Normal distribution tests results of nine OBPs. **Figure S3.** Normal Q-Q plot results of nine OBPs. **Figure S4.** Equal variances test results of nine OBPs. (PDF 501 kb)

## Abbreviations

CSPs: Chemosensory proteins
DEET: N,N-diethyl-meta-toluamide
GO: Gene ontology
GPCRs: G protein-coupled receptors
GRs: Gustatory receptors
iGluRs: Ionotropic glutamate receptors
IRs: Ionotropic receptors
OBPs: Odorant binding proteins
Orco: Odorant receptor co-receptor
ORFs: Open reading frame
ORNs: Olfactory receptor neuron
ORs: Odorant receptors
PR: Pheromone receptor
SBCM: *Eogystia hippophaecolus*
SNMPs: Sensory neuron membrane proteins.
