# Supplementary material for: Antennal transcriptome analysis and expression profiles of odorant binding proteins in Eogystia hippophaecolus (Lepidoptera: Cossidae)
Source: BMC Genomics. 2016 Aug 18;17:651. doi: 10.1186/s12864-016-3008-4 (PMC4989532; doi:10.1186/s12864-016-3008-4)
Supplement: Additional file 1: — Best blastx hits for putative odorant binding proteins (OBPs), sensory neuron membrane proteins(SNMPs), odorant receptors(ORs), ionotropic receptors(IRs), and gustatory receptors(GRs) of Eogystia hippophaecolus. Table S1, Table S2, Table S3, Table S4. Table S1. Sequence information and best blaxts match information of putative odorant binding proteins (OBPs). Table S2. Sequence information and best blaxts match information of sensory neuron membrane proteins (SNMPs). Table S3. Sequence information and best blaxts match information of Odorant receptors (ORs). Table S4. Sequence information and best blaxts match information of ionotropic receptors (IRs). Table S5. Sequence information and best blaxts match information of gustatory receptors (GRs). (PDF 233 kb) [file 12864_2016_3008_MOESM1_ESM.pdf]

Antennal transcriptome analysis and expression profile of odorant binding proteins in *Eogystia hippophaecolus* (Lepidoptera: Cossidae)

Ping Hu[a], Jing Tao[a] , Mingming Cui[a], Chenglong Gao[a], Pengfei Lu[a] , Youqing Luo[a]

Additional file1

Best blastx hits for putative odorant binding proteins (OBPs), sensory neuron membrane proteins(SNMPs), odorant receptors(ORs), ionotropic receptors(IRs) and gustatory receptors(GRs) of *Eogystia hippophaecolus*

Table S1 Best blastx hits for putative odorant binding proteins of *Eogystia hippophaecolus*

								Best Blast Match					
**Number**	**Gene ID**	**Gene length(bp)**	**ORF length(bp)**	**Completed ORF**	**Signal peptide**	**Male FPKM**	**Female FPKM**	**Name**	**ACC. Number**	**Species**	**Score**	**E-value**	**Identity**
HhipOBP1	c7136_g1	582	459	Y	N	35.02	28.77	odorant binding protein 1	AFD34177.1	*Argyresthia conjugella*	125	1.00E-32	50%
HhipOBP2	c31984_g4	976	726	Y	Y	171.52	226.07	odorant binding protein fmxg18C17 precursor	NP_001157372.1	*Bombyx mori*	237	4.00E-73	49%
HhipOBP3	c36615_g1	320	306	N	Y	0.00	0.67	odorant binding protein LOC100301496 precursor	NP_001153664.1	*Bombyx mori*	67.4	1.00E-11	33%
HhipOBP4	c23833_g1	3233	420	Y	N	11702.90	6246.99	odorant binding protein	AGM38607.1	*Chilo suppressalis*	174	2.00E-46	75%
HhipOBP5	c27927_g1	4663	441	Y	N	1243.17	925.32	odorant binding protein	AGM38605.1	*Chilo suppressalis*	405	2.00E-60	72%
HhipOBP6	c19939_g1	738	378	Y	N	0.88	2.69	odorant binding protein	AII01008.1	*Dendrolimus kikuchii*	232	7.00E-73	65%
HhipOBP7	c16117_g1	646	345	Y	N	1.31	0.65	odorant-binding protein	AEX07279.1	*Helicoverpa armigera*	194	6.00E-59	68%
HhipOBP8	c31073_g1	2496	312	Y	N	32.68	12.41	odorant binding protein 11	AGP03457.1	*Spodoptera exigua*	166	9.00E-44	60%
HhipOBP9	c5992_g1	1097	402	Y	Y	3662.71	6274.55	odorant binding protein 9	AGH70105.1	*Spodoptera exigua*	243	3.00E-76	86%
HhipOBP10	c19074_g1	933	720	Y	Y	19.31	31.56	odorant binding protein11	AGP03457.1	*Spodoptera exigua*	166	9.00E-44	60%

EhipOBP11	c654_g1	400	222	N	N	0.58	0.20	odorant binding protein 19	ALD65893.1	*Spodoptera litura*	68.2	1.00E-11	56%
EhipOBP12	c11842_g1	1177	756	Y	Y	45.87	112.77	odorant-binding protein 4	ALT31634.1	*Cnaphalocrocis medinalis*	333	1.00E-110	61%
EhipOBP13	c18654_g1	804	444	Y	N	816.17	1869.63	odorant-binding protein 1	AFG72998.1	*Cnaphalocrocis medinalis*	233	1.00E-74	74%
EhipOBP14	c19159_g1	764	459	Y	N	1745.90	3628.80	odorant-binding protein	AAR28762.1	*Spodoptera frugiperda*	134	5.00E-35	45%
EhipOBP15	c27927_g2	1621	366	Y	N	938.86	493.99	odorant-binding protein 1	EHJ65653.1	*Danaus plexippus*	207	2.00E-60	78%
EhipOBP16	c31534_g1	1981	420	Y	N	39.88	47.30	odorant-binding protein 1	AGK24577.1	*Chilo suppressalis*	108	4.00E-24	36%
EhipOBP17	c32463_g4	1486	363	Y	N	13.94	4.17	odorant binding protein	AII00969.1	*Dendrolimus houi*	158	5.00E-43	52%
EhipOBP18	c16693_g1	603	420	Y	N	2089.96	1438.87	odorant binding protein 8	AKI87969.1	*Spodoptera litura*	226	4.00E-73	85%
EhipOBP19	c23004_g1	1255	522	Y	N	8.13	8.33	odorant-binding protein 25	ANC68513.1	*Chilo suppressalis*	143	2.00E-36	55%
EhipOBP20	c29509_g3	3375	309	Y	N	19.08	43.58	SexiOBP10	AGP03456.1	*Spodoptera exigua*	112	3.00E-24	63%
EhipOBP21	c38862_g1	627	496	N	N	0.45	0.30	SexiOBP9	AGP03455.1	*Spodoptera exigua*	155	1.00E-43	54%
EhipOBP22	c21596_g1	1470	543	Y	N	2823.19	3233.83	antennal binding protein 5	AAL60423.1	*Manduca sexta*	168	1.00E-45	49%
EhipOBP23	c27810_g2	3683	426	Y	N	274.26	280.07	odorant binding protein 5	AFD34173.1	*Argyresthia conjugella*	226	5.00E-65	78%
EhipOBP24	c29324_g1	1430	414	Y	N	27.21	51.23	odorant-binding protein 18	ALT31648.1	*Cnaphalocrocis medinalis*	192	3.00E-56	67%
HhipGOBP1	c20085_g1	861	600	Y	Y	33136.69	45534.35	general odorant binding protein 1	AFP66957.1	*Cydia pomonella*	230	1.00E-71	80%
HhipGOBP2	c10856_g2	954	501	Y	N	14972.03	31357.86	general odorant binding protein	AEZ52491.1	*Orthaga achatina*	275	8.00E-89	77%
HhipPBP1	c29512_g2	863	498	Y	N	50912.89	62237.13	pheromone-binding protein 3	AHZ89399.1	*Grapholita molesta*	236	3.00E-74	63%
HhipPBP2	c12955_g1	3526	498	Y	Y	21271.65	6853.11	pheromone binding protein	AAF06141.1	*Pectinophora gossypiella*	317	1.00E-76	71%
HhipPBP3	c29244_g1	1895	507	Y	Y	1902.94	1926.05	pheromone binding protein 2	AGH13203.1	*Plutella xylostella*	217	8.00E-63	58%

Table S2 Best blastx hits for sensory neuron membrane protein of *E. hippophaecolus*

Number	Gene ID	Gene length	ORF length (bp)	Completed ORF	Male FPKM	Female FPKM	Best Blast Match: Name	ACC. Number	Species	Score	E-value	Identity
HhipSNMP1	c32085_g1	3791	1539	Y	267.60	426.16	sensory neuron membrane protein 1	ADQ73892.1	*Ostrinia nubilalis*	754	0.00E+00	74%
HhipSNMP2	c26041_g1	2411	1560	N	1288.55	991.36	sensory neuron membrane protein 2	ADQ73889.1	*Ostrinia nubilalis*	779	0.00E+00	73%

Table S3 Best blastx hits for putative odorant receptors of *E. hippophaecolus*

Number	Gene ID	Unigene Length(bp)	ORF Length(bp)	Complete ORF	Male FPKM	Female FPKM	Best Blast Match: Name	ACC. Number	Speceies	Score	E-value	Identity
HhipPR1	c13014_g1	1701	1290	Y	440.17	136.13	olfactory receptor 1	CBH19583.1	*Antheraea pernyi*	391	4.00E-127	46%
HhipOR1	c14018_g1	1215	987	Y	1.11	2.66	olfactory receptor 15	NP_001091789.1	*Bombyx mori*	374	4.00E-123	53%
HhipOR2	c27608_g1	1762	1194	Y	1.87	2.79	olfactory receptor 15	NP_001091789.1	*Bombyx mori*	419	4.00E-138	55%
HhipOR3	c26944_g1	1397	1203	Y	1.20	1.38	olfactory receptor 17	NP_001157210.1	*Bombyx mori*	387	3.00E-127	45%
HhipOR4	c24637_g1	1550	1164	Y	17.47	29.01	olfactory receptor 16	NP_001104832.2	*Bombyx mori*	572	0.00E+00	69%
HhipOR5	c38176_g1	1625	1203	Y	0.57	1.20	olfactory receptor 8	NP_001157209.1	*Bombyx mori*	539	0.00E+00	76%
HhipOR6	c29879_g1	1720	1200	Y	7.97	8.61	olfactory receptor 35	AIT69889.1	*Ctenopseustis herana*	524	0.00E+00	62%
HhipOR7	c30360_g2	1043	702	Y	82.22	28.84	olfactory receptor 22	AIT69882.1	*Ctenopseustis herana*	242	1.00E-73	49%
HhipOR8	c31027_g1	2047	384	Y	39.84	102.97	olfactory receptor 67	AIT69909.1	*Ctenopseustis herana*	156	5.00E-38	58%
HhipOR9	c30526_g1	1549	1302	Y	0.00	10.77	olfactory receptor 11	AIT69876.1	*Ctenopseustis herana*	330	3.00E-104	40%
HhipOR10	c20905_g1	1370	1254	Y	2.26	0.96	olfactory receptor 67	AIT72018.1	*Ctenopseustis obliquana*	420	8.00E-140	48%
HhipOR11	c25804_g1	1773	1149	Y	53.95	82.49	olfactory receptor 22	AIT71991.1	*Ctenopseustis obliquana*	358	4.00E-114	44%
HhipOR12	c30494_g1	3143	1194	Y	1.74	1.84	olfactory receptor 9	AIT71983.1	*Ctenopseustis obliquana*	438	7.00E-140	64%
HhipOR13	c10266_g1	1285	1221	Y	0.94	1.56	olfactory receptor 61	AIT72014.1	*Ctenopseustis obliquana*	330	2.00E-105	49%

HhipOR14	c22361_g1	1450	1233	Y	0.28	20.13	olfactory receptor 22	AIT71991.1	*Ctenopseustis obliquana*	353	1.00E-113	43%
HhipOR15	c26131_g1	2127	1227	Y	6.50	23.97	olfactory receptor 11	AIT71985.1	*Ctenopseustis obliquana*	354	2.00E-111	45%
HhipPR2	c26484_g1	1651	1239	Y	15.84	23.50	odorant receptor 3	AFC91713.2	*Cydia pomonella*	338	2.00E-106	43%
HhipOR16	c21084_g1	2042	1266	Y	526.82	84.16	olfactory receptor	EHJ75140.1	*Danaus plexippus*	171	3.00E-46	56%
HhipOR17	c27762_g1	1907	1266	Y	0.06	44.59	olfactory receptor	EHJ75140.1	*Danaus plexippus*	197	1.00E-55	62%
HhipOR18	c36145_g1	1179	1079	N	0.35	0.25	olfactory receptor 29	EHJ78030.1	*Danaus plexippus*	535	0.00E+00	72%
HhipOR19	c26052_g1	1384	1191	Y	4.34	5.32	odorant receptor	AII01045.1	*Dendrolimus houi*	380	2.00E-124	47%
HhipOR20	c23838_g1	1737	1161	Y	1.75	3.18	odorant receptor	AII01058.1	*Dendrolimus houi*	434	3.00E-144	59%
HhipOR21	c27681_g1	1473	1170	Y	5.54	7.79	odorant receptor	AII01110.1	*Dendrolimus kikuchii*	436	5.00E-146	51%
HhipOR22	c25978_g1	1550	1287	Y	11.98	18.57	odorant receptor	AII01085.1	*Dendrolimus kikuchii*	701	0.00E+00	78%
HhipOR23	c25082_g1	1619	1185	Y	13.23	15.92	odorant receptor	AII01084.1	*Dendrolimus kikuchii*	526	1.00E-180	70%
HhipOR24	c28753_g1	2134	1161	Y	7.38	10.27	odorant receptor	AII01092.1	*Dendrolimus kikuchii*	426	5.00E-139	59%
HhipOR25	c27322_g1	2356	1251	Y	7.39	13.07	odorant receptor	AII01083.1	*Dendrolimus kikuchii*	517	3.00E-173	59%
HhipOrco	c29483_g1	2065	1422	Y	260.03	206.56	olfactory receptor-2	BAG71418.1	*Diaphania indica*	912	0.00E+00	90%
HhipOR26	c28921_g1	1819	1245	Y	3.80	5.98	odorant receptor 3	ACJ12929.2	*Epiphyas postvittana*	488	3.00E-164	58%
HhipOR27	c20108_g1	1468	1236	Y	4.91	7.46	odorant receptor	AIG51875.1	*Helicoverpa armigera*	535	0.00E+00	67%
HhipOR28	c29513_g1	1648	1290	Y	42.06	31.17	odorant receptor	AIG51891.1	*Helicoverpa armigera*	495	1.00E-167	63%
HhipOR29	c30817_g1	1947	1185	Y	4.87	2.89	odorant receptor	AIG51902.1	*Helicoverpa armigera*	359	2.00E-114	47%
HhipOR30	c32133_g1	1829	1206	Y	8.09	12.31	odorant receptor	AIG51887.1	*Helicoverpa armigera*	564	0.00E+00	73%
HhipOR31	c27151_g1	1462	1215	Y	8.15	15.61	odorant receptor	AIG51879.1	*Helicoverpa armigera*	458	1.00E-154	56%
HhipOR32	c30040_g2	1875	1323	Y	6.94	7.87	odorant receptor	AIG51892.1	*Helicoverpa armigera*	530	3.00E-180	59%
HhipOR33	c31240_g1	2890	1179	Y	9.35	10.22	odorant receptor	AIG51873.1	*Helicoverpa armigera*	477	8.00E-156	62%
HhipOR34	c28211_g2	1542	1068	Y	10.65	9.56	olfactory receptor 4	ACF32962.1	*Helicoverpa armigera*	524	1.00E-179	74%
HhipOR35	c31439_g2	2553	579	Y	16.55	18.32	olfactory receptor 43	AJD81577.1	*Helicoverpa assulta*	252	1.00E-73	58%
HhipOR36	c21762_g1	1380	1206	Y	6.34	5.97	odorant receptor 62	AFL70825.1	*Manduca sexta*	469	3.00E-159	56%
HhipOR37	c31099_g1	1794	678	Y	5.49	3.43	putative olfactory receptor 9	BAR43451.1	*Ostrinia furnacalis*	401	1.00E-100	66%

HhipOR38	c7064_g1	1625	1215	Y	0.80	0.76	olfactory receptor OR44	AJE25889.1	*Planotortrix excessana*	144	3.00E-34	29%
HhipOR39	c27715_g1	1432	897	Y	1.04	5.93	olfactory receptor OR25	AJE25878.1	*Planotortrix excessana*	452	1.00E-152	56%
HhipOR40	c30401_g1	1443	1221	Y	20.30	53.81	olfactory receptor OR57	AJE25897.1	*Planotortrix excessana*	189	8.00E-51	30%
HhipOR41	c32164_g1	1800	1209	Y	16.12	56.94	olfactory receptor OR27	AJE25880.1	*Planotortrix excessana*	527	8.00E-180	59%
HhipOR42	c32615_g1	4943	1359	Y	5.32	8.14	olfactory receptor OR47	AJE25892.1	*Planotortrix excessana*	640	0.00E+00	75%
HhipOR43	c29160_g1	2614	786	Y	8.84	13.50	olfactory receptor OR18	AJE25875.1	*Planotortrix excessana*	580	2.00E-155	72%
HhipOR44	c17125_g1	1656	1131	Y	5.22	10.10	olfactory receptor OR53	AJF23811.1	*Planotortrix octo*	256	1.00E-75	46%
HhipOR45	c24505_g1	1397	1206	Y	6.46	9.07	olfactory receptor OR59	AJF23815.1	*Planotortrix octo*	617	0.00E+00	76%
HhipOR46	c25024_g1	1731	1221	Y	16.12	19.17	olfactory receptor OR72	AJF23825.1	*Planotortrix octo*	347	3.00E-110	44%
HhipOR47	c28736_g1	1459	1224	Y	4.04	5.44	olfactory receptor OR10	AJF23787.1	*Planotortrix octo*	585	0.00E+00	62%
HhipOR48	c32504_g1	1758	1221	Y	7.04	8.55	olfactory receptor OR54	AJF23812.1	*Planotortrix octo*	242	4.00E-70	38%
HhipPR3	c30396_g1	1618	1272	Y	24.32	21.14	odorant receptor 13a-like	NP_001292415.1	*Plutella xylostella*	371	1.00E-119	47%
HhipOR49	c4188_g1	559	504	N	1.08	1.72	olfactory receptor 13	NP_001166603.1	*Bombyx mori*	182	4.00E-52	56%
HhipOR50	c11540_g1	722	510	Y	1.12	0.79	olfactory receptor 13	NP_001166603.1	*Bombyx mori*	231	5.00E-70	57%
HhipOR51	c18392_g1	377	276	N	19.33	38.11	olfactory receptor 27	AIT69885.1	*Ctenopseustis herana*	136	1.00E-35	63%
HhipOR52	c28482_g7	547	183	Y	0.23	0.83	olfactory receptor 54	AIT69900.1	*Ctenopseustis herana*	75.9	5.00E-13	68%
HhipOR53	c32853_g5	414	189	N	8.11	7.63	putative odorant receptor OR43	AFC91751.1	*Cydia pomonella*	54.3	7.00E-07	50%
HhipOR54	c27593_g1	868	333	N	6.09	9.85	odorant receptor	AIG51887.1	*Helicoverpa armigera*	204	2.00E-43	55%
HhipOR55	c12406_g1	527	267	Y	2.11	1.16	odorant receptor	AIG51899.1	*Helicoverpa armigera*	174	4.00E-49	64%
HhipOR56	c40921_g1	406	197	N	0.37	0.00	olfactory receptor 40	AJD81574.1	*Helicoverpa assulta*	112	1.00E-16	42%
HhipOR57	c9881_g1	554	267	Y	0.43	0.35	odorant receptor 50	AFL70813.1	*Manduca sexta*	68.9	1.00E-10	89%
HhipOR58	c30360_g1	632	558	N	108.40	38.07	olfactory receptor OR22	AJF23793.1	*Planotortrix octo*	137	2.00E-34	38%
HhipOR59	c41377_g1	305	246	N	0.00	1.52	olfactory receptor OR27	AJF23796.1	*Planotortrix octo*	93.6	3.00E-20	55%

TableS4 Best blastx hits for ionotropic receptors of *E. hippophaecolus*

							Best Blast Match					
**Number**	**Gene ID**	**Unigene Length(bp)**	**ORF Length(bp)**	**Complete ORF**	**Male FPKM**	**Female FPKM**	**Name**	**ACC. Number**	**species**	**Score**	**E-value**	**Identify**
*Ehip*IR75q2a	c21361_g1	1045	990	N	1.75	1.32	ionotropic receptor	BAR64808.1	*Ostrinia furnacalis*	420	1.00E-138	60%
*Ehip*IR75q2b	c21361_g2	1263	666	Y	2.39	1.38	ionotropic receptor	BAR64808.1	*Ostrinia furnacalis*	533	1.00E-141	79%
*Ehip*IR8a	c22122_g1	2997	2703	Y	17.82	21.21	ionotropic receptor	BAR64796.1	*Ostrinia furnacalis*	1467	0.00E+00	80%
*Ehip*IR41a	c24503_g1	2006	1803	Y	3.00	2.02	ionotropic receptor	BAR64800.1	*Ostrinia furnacalis*	850	0.00E+00	69%
*Ehip*IR25a	c26331_g1	3014	2793	Y	37.23	34.28	ionotropic receptor	BAR64798.1	*Ostrinia furnacalis*	1623	0.00E+00	92%
*Ehip*IR75q1	c30701_g1	2679	1560	Y	15.51	4.11	ionotropic receptor	BAR64803.1	*Ostrinia furnacalis*	644	9.00E-175	52%
*Ehip*IR31a	c31718_g1	2283	1863	Y	7.85	6.09	ionotropic receptor	BAR64813.1	*Ostrinia furnacalis*	415	1.00E-134	59%
*Ehip*IR76b	c32775_g2	3397	1635	Y	130.28	247.77	ionotropic receptor	BAR64809.1	*Ostrinia furnacalis*	779	0.00E+00	67%
*Ehip*IR75p1	c34630_g1	326	192	Y	0.31	0.00	ionotropic receptor	BAR64807.1	*Ostrinia furnacalis*	113	1.00E-26	72%
*Ehip*IR21a	c29422_g1	2884	2571	Y	5.84	6.98	ionotropic receptor	BAR64797.1	*Ostrinia furnacalis*	1222	0.00E+00	73%
*Ehip*IR75p2	c32337_g1	1609	1218	Y	3.70	4.27	ionotropic receptor	BAR64805.1	*Ostrinia furnacalis*	577	0.00E+00	70%
*Ehip*IR68a	c14556_g1	2169	2101	N	0.67	0.47	putative chemosensory ionotropic receptor IR68a	ADR64682.1	*Spodoptera littoralis*	1011	0.00E+00	73%

Table S5 Best blastx hits for gustatory receptors of *E. hippophaecolus*

Number	Gene ID	Unigene Length(bp)	ORF Length(bp)	Complete ORF	Male FPKM	Female FPKM	Best Blast Match					
							Name	ACC. Number	Species	Score	E-value	Identity
HhipGR1	c1747_g1	714	417	Y	0.37	2.23	TPA: gustatory receptor 57	DAA06391.1	*Bombyx mori*	70.5	1.00E-10	33%
HhipGR2	c2204_g1	468	285	Y	2.74	3.36	gustatory receptor 46	ACD85125.1	*Bombyx mori*	58.2	4.00E-07	38%
HhipGR3	c3627_g1	1129	1083	Y	0.26	0.67	TPA: gustatory receptor 17	DAA06380.1	*Bombyx mori*	139	1.00E-33	27%
HhipGR4	c6427_g1	551	192	Y	1.32	0.80	TPA: gustatory receptor 58	DAA06392.1	*Bombyx mori*	62.8	2.00E-08	60%
HhipGR5	c37098_g1	337	312	N	0.56	0.59	gustatory receptor 68	NP_001233217.1	*Bombyx mori*	89.7	7.00E-19	41%
HhipGR6	c39266_g1	579	366	Y	0.00	0.87	TPA: gustatory receptor 11	DAA06375.1	*Bombyx mori*	71.6	2.00E-11	32%
HhipGR7	c40435_g1	690	285	Y	0.32	0.59	TPA: gustatory receptor 63	DAA06395.1	*Bombyx mori*	130	7.00E-32	61%
HhipGR8	c47186_g1	567	408	Y	1.79	0.00	TPA: gustatory receptor 63	DAA06395.1	*Bombyx mori*	89.7	8.00E-18	36%
HhipGR9	c49142_g1	377	363	N	0.43	0.00	TPA: gustatory receptor 63	DAA06395.1	*Bombyx mori*	88.6	5.00E-18	49%
HhipGR10	c51163_g1	330	268	N	0.89	0.00	gustatory receptor 68	NP_001233217.1	*Bombyx mori*	72	2.00E-12	48%
HhipGR11	c22101_g1	926	354	Y	10.18	9.35	putative gustatory receptor candidate 59	EHJ69979.1	*Danaus plexippus*	101	8.00E-23	67%
HhipGR12	c41858_g1	563	552	N	0.86	0.00	gustatory receptor 10	AJD81603.1	*Helicoverpa assulta*	91.7	6.00E-19	41%
HhipGR13	c42417_g1	1829	1188	Y	0.12	0.54	gustatory receptor 5	AGK90025.1	*Helicoverpa assulta*	183	1.00E-47	29%
