# Supplementary material for: Antennal transcriptome analysis and expression profiles of odorant binding proteins in Eogystia hippophaecolus (Lepidoptera: Cossidae)
Source: BMC Genomics. 2016 Aug 18;17:651. doi: 10.1186/s12864-016-3008-4 (PMC4989532; doi:10.1186/s12864-016-3008-4)
Supplement: Additional file 2: — Nucleic acid sequences of all candidate chemosensory proteins identified in Eogystia hippophaecolus transcriptome. (PDF 586 kb) [file 12864_2016_3008_MOESM2_ESM.pdf]

Antennal transcriptome analysis and expression profiles of odorant binding proteins in *Eogystia hippophaecolus* (Lepidoptera: Cossidae)

Ping Hu[a], Jing Tao[a], Mingming Cui[a], Chenglong Gao[a], Pengfei Lu[a], Youqing Luo[a]

Additional file 2

Nucleic acid sequences of all candidate chemosensory proteins identified in *Eogystia hippophaecolus* transcriptome

>EhipOBP1
ATGGCCGTAAAAATTTTCATTGCTATGTTTGTCGCTATCTTTGCGGCAACAATGCAAAGT
ACCGAAGCAATAACCGAAGAGAAGAAAACGGAGCTTAAAGCCAAGTTAGTCCCTATTTTA
ACTGAATGCGGCAAGGAGCACAGCATCACATTGGATTATTTGAAGGAATTCAGACAACAA
CAAAAGCGTCCTGATGAATCTAACGCATGTTTCTTTGCGTGTGTCTTCCAGAAGACCGGA
CTTTTGGATGACAAAGGCTTATTCGTCGAAGCTAAAGCAGCTGAAAGAGGCCAATATTAT
GCTGAGGAGGGTGAATTGGAGAAGGCAAATGAAGCAGTCAAAGCATGTGTATCAGTAAAC
GAGCAAAGCGTGAGCGACGGACCGAAGGGCTGCGATCGTGCCAAATTGATCTACAACTGC
TTCTTGGACCAGAAGCAACATTTCGGCTACGTATATTGA

>EhipOBP2
ATGTTTAACAAATTCACATTCGTAACAAGTTTTTTTACTATTTTGTATGTAACGAACGCCG
ATCCTGCTTCATTTATAACAAAATGCAAATCGGATGATACAAAATGTACCAAAGAATCAA
CTCAAAAAGCTATCCCAATATTTGCTGATGGTATCACTGAGTTGGGAGTAGAACCATTGG
ATCCTTTCGTCTTAAAGAAAATAGATGCAAGCACACCAGTGTTAAAGTTTATGTTGAGTG
ATATGACAATAACAGGACTCAAAAATTGCCAAGCCAAGAAAATTCAGCGGAACGTAGCAA
ATTCAGAATTGATTGTGAAAACCCTATGTGACGTCGATGCAGATGGACATTACGATATGT
CCGGACAACTAATCATTTTACCCATAGAAGGCAACGGAAAAATTCATGTCAAACTAAGGA
AATTGCAGATGGTCGTGGTAGCAGAATTATTTGATAAAGAAGGTACAGATGGAAAGAAAC
ATTGGGAAATCAAGCATTGGAAACACTCTTTCGATTTAAAAGACAAGGCGGGTCTTGAAT
TCGAGAACTTGTTTAACGGCAATGAAGCGCTAGCTGGTGCGGTTCGCGAACTAATAGCAT
CCAGTGGCAATGAAATTATAACAGAAGTTGGTGCACCTGTAATTAAGGCCATTACAGAAA
AAATTGTTCAGAATGTCCAGAATTTCTTCAGAGCCGTGCCTATTGAAGACCTCGCTTTAG
ATTGA

>EhipOBP3
ATGAATATTCAAATTACATTCGTTACCATATTGCTAATGAAATTCTCT
GTCTGTGCTTATTGTGATCCATCATTATCAGACAATCATAAGAGAAAGTTAAGTCATCAT
TTTGTGACTTTTGGTGTAGGATGTTTGGCCAAACACAACGTGTCTTTTGAAGATATTCGC
GCTTTACAAAATTTTAATATGCCCAAAGGAAAAGATGCGCCTTGTTTTTTTGCTTGTATA

TTAAAAAAAAGCCAATATGATAGATTCTAAAGGTAAGGTTTCTCCAGAGGCAGTGGCTGAG
GTTGCAAAAATGTTATTT

>EhipOBP4
ATGGCGATGGAAAGTTCGACGCTGTGTATGGTACTAGTCGCGCTGGTGGCGGGCGCGGCCGCCATGGACG
AAGAGATGGCGGAGCTGGCTCGCATGCTGCGAGAAAACTGCGGCGAGGAGACCGGAGTCGACCTGGGCC
T
GGTGGACCAGGTGAACGGCGGGGCGGACTTGATGCCCGACCCGAAGCTCAAATGTTACATCAAGTGCGTC
ATGGAGACGGCGGGAATGTTTTCCGAGGGGCAAGTGGAAGTAGAAGCGGTGATCGCAATGTTGCCTGAAA
ATATGAAGAAGAGCGAGGGGTCGATCCGAGGGTGCGGCACGCAGAAGGGCGCCGACGACTGCGACACGG
C
GTTCTTGACGCAGGTCTGCTGGCAAAAAGCTAACAAGGCGGAATACTTCCTCGTGTGA

>EhipOBP5
ATGATTTCCCTGAATAATTCACTCTTTGTTTATATTATAT
TTTTTTTAATTTTAAGCAGAACTTTTGTAATGTCGATGACCAGGCAACAAATAAAGAATT
CTGGGAAACTATTAAAAAAGTCGTGTATGCCGAAAAATGACGTCACAGAAGATCAAGTGG
GCAACATAGAGCAAGGCAAATTCATCGAAAACAAGAATGTGATGTGTTACATAGCGTGTA
TTTATAGTATGGGACAAGTTGTAAAGAATAATAAAATAGTTTTTGATGCAATGATAAAAC
AAGTTGATATGATGTTTCCGCCAGAAATGAAGGAGCCTTTTAAGGAATCGATAGAAAAAT
GCAAAGGAGTTCCAAAGAAGTACAAAGACATCTGCGAAGCTTCTTATTGGACAGCTAAAT
GTCTCTACGATGCCGACCCAGCAAATTTCATCTTCCCATGA

>EhipGOBP1
ATGGCCAGTCATTCATTGTG
GTCAATCAGTTATGCATATAACGCGCCAGGGAGCGGTGGAACGATCCAGTCTCTGGACGT
GTATCAGGTCGAAACAATGGAACGGAAAATGTTGAAGTTGATAGGAATATTATTATGCTC
GCTGTTGGCGGGCTCGCTGGCAACCGTGGAAGTGATGAAGGATGTCACGCTCGGTTTTGG
AGAAGCCTTGAAACATTGCAGAGAAGAAAGTCAATTGTCAGAGGAGCAGATGGAGGAGTT
CTTCCACTTCTGGCGCGACGACTTCAAGTTTGATCTCCGGGAGATCGGCTGCGCCATCCG
CTGCATGAGCCACCACTTCAACCTTCTCACCGACTCCCACCGCATGCATCACGAGAACAC
CGACAAGTTCATCAAGTCCTTCCCCAATGGTGAGCTGTTGTCGCAAAAGATGGTGACACT
GATCCACGAGTGTGAGAAGCAATTCGAGTCAGAGGAAGACCACTGTTGGCGGATACTACG
AATAGCTGAATGCTTCAAAACGGGCTGCAAGCAGCAGGGCATCGCTCCTACGATGGAGAT
GATCATGGCCGAGTTCATCATGGAGTCTGAAAGGCGATGA

>EhipOBP6
ATGAAATGCATATTCAG
GAAAGCGGGATGGCTTGATGGCGATAAGGTGGACAAAGAGAAAGTGACGGCTCACTTCGA

CCAATTCGCCAAAGACAACCCGAGTTGGTCTCCAGCCGTGCAGTACGTGAAAGCTGCTTG
CTTGGCCACAGACCTTCCAGCCCAAGGTGTCTACATCAACTGTCCAGCTTATGACGTCGT
GCACTGTTCTTTGACTGGATTCTTTAAGAACGCGCAGGCTTCCCAATGGTCGACGTCACA
GGAGTGCGCGTACCCGCGCCAGTTCGCGCAGGCCTGCCCCGTGTGCCCCGGCGACTGCTT
CGCGCCGGCGGTGCCCTACGGCTCTTGTAACGCTTGTCGTCTACTGCCGCAGACGCCCTG

>EhipOBP7
ATGAAATGCCACAAAGAACATCCAGTGGATATGTCCGAACTGTTGCAGC
TTCAAAAATTAATTGTACCTAAGAAAACCGAGACAAAATGTCTATTGGCCTGTGCGTACA
AGATTGATGGAATGCTAAACGACAAAGGCATGTATGATATAGAACACGCATACAAAAGAG
CTGAATTATCAAAAAATGGTGATGAAAAACGATTAGTAAATGCAAAGAAATTAGCGGATA
TTTGTGTTAAAGTGAACGATGTGGAAGTAAGCGACGGTGAAAAGGGTTGTGAGAGGTCGG
CGTTGATGTTCAAATGTCTCATTCATAATGCTCCCAAGTTAGGATTCAAGTTGTGA

>EhipGOBP2
ATGGCAGCTATTTACTGTTTGTTGTTTTCCTTGGTTATTTTTGTT
TCAATCACTGATGAAGTGATGGGAACAGCTGAGGTGATGAGTCATGTTACGGCACATTTT
GGGAAATCATTGGATGAATGTCGGGAAGAGTCAGGACTCTCTTCAGAAATCATGGAGGAG
TTCAAGCACTTCTGGAGCGAAGATTTCGAGGTGGTTCACCGCGAGCTAGGATGTGCTATT
ATTTGCATGTCGAATAAATTCTCGCTGCTCAAAGACGATACTAGGATGCATCACATGAAC
ATGGATGACTATATCAAAAGCTTCCCTAAAGGCGATATCCTCTCAGCGAAGATGGTGGAA
TTGATCCACAATTGCGAGAAGCAGTATGATGATATAAAAGATGACTGCAGTCGGGTGGTG
AAGGTGGCAGCTTGCTTCAAAGTAGATGCAAAGAAAGAGGGAATAGCGCCAGAAGTAACT
ATGATAGAAGCAGTCATGGAACAGTACAATAATTAA

>EhipOBP8
ATGCCGAACTTAGAAGAGTTGTCAATCATGCACTTGTACGCTAACATGGCACGTTCACA
ACTCACCTCACCACTTCCAACTGGGTCGGTATTAACATGGGAGCAAGAATGCAGGTACTC
TTCAATTTTCTTCAGTTCGTCGGCATCTTTGATAACTTCTTTGATTAGTTCTAGTGCTGT
TTCTTTTCTTAAATTGCCGCCGTCTTCCATAATGCCCATTTTTTTCATCATGCAGGCTAA
GAAACACGGAACATTTTCACCAGAGGCAATTTTCCTGGCTCGTAGTTCAGTAATATCGGC
TTCCGAGATCTGA

>EhipOBP9
ATGAGAA
CTTTGCTGGTATTCGCTGTCTGCTTCGTAGTTGCTCAGGCTTTGACCGACGAGCAAAAAG
ACAAATTGAAGAAACACCGGTCCGAGTGTCTCTCTGAGACGAAGGTCGACCAGCAGCTGG
TCGATAAACTCAAGAGTGGAGACTTCAAGACCGAAAATGAACCTCTTAAAAAGTATTCCC
TCTGCATGCTCATGAAGTCAGAACTGATGACCAAGGACGGCAAATTCAAGAAGGACGTTG

CTCTAGCTAAAGTTCCTAATGAAGCCGACAAGCCCACAGTTGAGAAGCTAATCGACACCT
GTTTGGCGAACAAAGGCAACAACCCGCACCAAACCGCTTGGAACTACGTCAAATGTTATC
ACGAGAAGGATCCGAAACATGCCATCTTCTTGTAA

>EhipOBP10
ATGGTAACTGTGGTAGCATTTCTCG
CGCTTTTGCCTTCATTGGTTACTTCTGCCACTGAAGGTAATATCCATCTTCTAGAAGATG
AGATAGCTCTCGCTTTACGCGCCTGTTCTTATCCGGACACGAATACGAACATTGTCAAAG
AAAATGAAGATCAACGTCAAAGCCGCTACGTGAGAAGCTCGAACAGATTCTACGAAGAAC
AATCACCAAGAATAGACAGTGGAGTGAAAGAGGACACGAATCAGTATGGGCATGAGAGAA
GAAATGCGACAGATATGCGTATACCTAAACAGATCCGTGTCTTAAATACAACAGAATATA
ATTATGGTTGTAATGATACTGAAAGGGGTGGTGAAAGACAGACCAGCTCTGTGACACGTC
CGGAATTTGTAAATGAATCAGCAAATAACACTAGAGTTAAACGAACTGAACCACTGCTGA
GTAAGGACGATTCTGATCAGTGTCTAAGCCAATGCGTTTTCGCTAATCTAGAAGTGATCG
ATTCAAGAGGAATTCCTCTGGAAGCCGAGCTCTGGAACAGAGTTCAGTCTTCATTGGCAT
CACAACAATCACGAGTTGCTCTGCGGGATCAGATCCGAGCTTGCTTCCAGGAGTTAGAAA
CAGATGGTAATGATAATGGGTGTTCATTTTCCAATAAACTTGAACGCTGCTTGATGTTAA
ATTTTTCTGAGCGGAAAATAAATGGTACAATTTGA

>EhipOBP11
ATGTCGCATAATATGTCTAGAAAAATGTATAGCCGATTGA
CAATTTTATTTTTGATTATTACTTATATAGCCGTTTGTGATGGCAACACCATGAATAATT
TACAAGAAAAGAATCGTATGGTTGGGGTGGATACTATTCATGACATCAGAATAGATAAAG
ATACAATAATTTCAAGAAACTTGAAATTGGAGAATAAACATCGCGGATGGAGTGGTGAGA
TTTGA

> EhipOBP12
ATGTCTTCATACAGT
ATTGTGTTTTTCTTTATACTATTTGCCTCTTCTTTTGGACAGATTGATATTGCAAAATAT
TTTAAGATATGTAACAGAACAGTGCAGGACGTGAATGAGTGTCTGGTAGAGGCTATCCAG
GAGGGTATCATTGCTATGTCCGGAGGCATTAAAGAACTAGGAGCGCCCCCTGTAGATCCC
TACCACCAAGAGGAAATACGAGTTGAATATAAAAATAATCAGATAATGGCTACAATGACT
ATGAAAGATATATATGTAGAAGGATTGAAACTAGCTAAAGTTCATAATGCAAGGTTACGA
GCAGATGAAGATTATTTTCATCTAGAAGTCGATATAACAACCCCGAGAGTATTTGTACGG
GGCAAATATATTGGAGAAGGACGGTACAATTCTTTGCGAATCAATGCTAAGGGTGAATTC
AACACTACTATGACTGACTTGGTTTACACGTGGAAAATTGACGGTATACCAGAAAAGAAA
AATAACACAACTTACATGCGAATTAAATCATTTTACATGCGACCTGATTTAGGAAATTTG
AAGGCATATATATCTAATGATAATATAGATAGTAAATCAATAATTGACTTCGCCAACAAT
TTTGCTAATCAAAACTGGAGGCCTTTGTATCGTGAAATGTTACCGTATGCCCAAGAGAAC
TGGAATAAAATTGGAATCCGTATTGCAAATAAAATATTTTTAAAAGTTCCTTATGATCAA
CTATTCCCTGAAGAAATGTAA

> EhipOBP13
A
TGAATATGCAAAAAGTTCATTTGTTCTGGTTTTTTATTGTTTTGATAGATTTTTTGCTCT
TCAACATCGGTTTTGCGATGACAAGACAACAATTTAAAAATTCGGGGAAACTGCTGAAGA
AATCGTGCATGCCGAAAAACGACGTTACCGAAGAACAAGTTGGGGAAATAGAACAAGGAA
AATTTATAGAGGATCGTAATGTCATGTGTTACATCGCTTGTATCTACACTATGTCGCAAG
TTGTGAAAAATAACAAAATTAATTATGAAGCCGTGATGAAACAAGTGGATATGTTGTTTC
CATCTGAACTCAAAGAGCCGGCAAAAGCCGCTGCGGCGAATTGCAAAGATGTGTCTAAGA
AACACAAGGATTTATGTGAAGCCGCATATTGGACTGCGAAATGTATGTACGATTTTGATC
CGAAAAGTTTCATATTTCCATAG

> EhipOBP14
ATGGCCTCCTTTTATTGTGTATTCTTG
TGTTACGGAATTGTGGCTCTTTATTTTATAAACGTTAATGCCGTGTCCCAAGAAGAGATA
ATCAAGATCGAAGGCGCATTACTACCATTCATCACTGAATGTAGTGCTCAGAATGGCGTT
AATATGGAAGATCTGACAGCGGCAAAGAAAAATGAAAACTATGATAACTTAAACCCTTGT
CTTATCGCTTGTGTGTTTAAAAAGACCGGAACGATGGACGATAAGGGACTGTTCAATTTA
GATAAAGCGTTGGAAAAAACCAAGAAATTCTTGAAATCTGAAGAGGACATCGATAAAGCA
GCCGAAGTAGCAAAATCTTGTGCCTCAGTAAATGATCAAGAAATAAGTGATAACGATAAG
AGTTGCGGGAGAGCAAAATTATTACTCGATTGTTTTATCAAACATAAAGGACAGTTCCCC
TTATCTATATAA

> EhipOBP15
ATGACGAGA
CAACAGTTAAAGAATTCATCAAAAATGTTGAAAAAACAATGTATGGGAAAAAATCAGGTC
ACAGAAGATTTAATAGGCGATATAGAAAAAGGGAAATTTATCGAAGACAGAAATGTTATG
TGTTACATTGCTTGCATATATCAAATGTTACAAGTGGTGAAGAATAATAAGTTGAGTTAT
GAGGCATCTCTTAAACAGGTGGATATGATGTATCCTGCAGATATAAAAGAGGCAGCAAAA
GCTACCATAACGAAATGTCAAGATGTTTCAAAAAAATACAAGGACTTGTGCGAATCTTCA
TATTGGACAGCGAAGTGCATTTATGAGGATGATCCAAAGAATTTTATCTTTCCGTAA

> EhipOBP16
ATGTAT
TACTTAATCGTGTTTTCTTTTATTCTATCTCTTGCATACACTAAATCTGCTCAGCAAGTT
ATACGTTTGGAACCAGATGTTAATTCTCGATTACTACCGATAATAACGCAATGCGTCAGC
GAAACTGGTTCCGATCCAAGCATAGTTCTTGAACTATCCAGCATGAAGATCAGCAACGAA
GAACAGTTTAAGAAGTTTGTTTATTGCGGTTTTACCAAATCTGATCTTACTACAGCCGAG
GGTCATTGGAAAATTGAAAAAGCCTTACAGTTTTATCCAAAAACTGTTGAAATAGCTCCA
CTTCAAAAGGCAATGGAATCGTGCAATAAAGAAAGTGGTCAAGATCCGGCAGATACAACG
TTTAAGATATTTAAATGCTTCATTAAGAGGACACCTGTGTTGATTTCATTTTAA

> EhipOBP17

ATGAGCCTAGGTAT
GGAATGCAATAAAGATTACCCGATTACACCGGATGAGGTGCAGACAATGATGCAAAAGTA
CCAAATTCCTAATAGCAAAAACGCTAAATGCTTGATGGCGTGTGTCTACAGGAAAGTCAA
TTGGTTGGACAGTAAAGGTACGTTCGATGTGGACAGTGCGAATTCAATGATGGAAAAAGA
ACACGCGGATGATCCCGCAAAGATGGAAAAGGCTAAAAAATTATTCGAAATTTGCAAGAA
AGTTAATGACGAACCAGTAACTGACGGCAATGAAGGTTGCGACCGATCTGCTCATATGTT
CCAATGTTTGGTTGAAAATGCTTTAAAGATGGGATTCAAAATACAATAA

> EhipOBP18
ATGATCCTAATTTTGCTTGTTAAATTATTGATATTAACAACTTTT
TGCAATGCGATGACAATGAAGCAAATAAAATCTACTGGAAAGATGATGAGAAAGTCGTGT
CAACCAAGAAATAACGTCGAAGATGAAAAAATCGATCCAATACAAAATGGCGTATTCATA
GAAGAAAAAGAAGTGATGTGCTACATGGCGTGTATCATGAAAATGGCAAATACGATAAAA
AATGGTAAATTGAACTACGACGCAGCTATTAAGCAGGCTGATCTGCTGTTACCAGAAGAA
ATAAAAGAACCAGCTAAAGAAGCTATCACAGCTTGCAAAAAAGTCGCGGACTCACACAAG
GACATCTGTGAAGCGTCATTTCACGTAACCAAATGTATTTACAAATATAATCCTGACATA
TTTTATTTCCCCTAA

> EhipOBP19
ATGTATAATAATAATTTTTGTTATGTTTGTTTCTTTTTATTTATTTTTA
TAATAAAAGATGCCTTGGCAATAACTCTAATTCAAAAAACTAGGATCCAAGCAAAGTTTC
TGGCAAGTGGAATTGCATGCATAAATGATTATCCTTTAACGGCGAATGATATTCAGATCT
TGAAGAGTAAAGTGATGCCTGATAATGAAAATGCAAATTGCTTTGTAGCTTGCCTGTTCA
AGAAAATTGGCATTATGGATGATATGGGTAAAATGACGCAGGCCGGAGCCAGGGAATTTG
CTAAGCAAGTTTTTAAGGACGATGATGAACCTTTAAAGAAAACCGATGAATTAATTGGAC
AATGTTCCTCCGTTAACGCGAATAGTGTGAGTGATGGAGACAAAGGGTGTGATCGAGCTA
GACTATTGTTCGTTTGTTTAGCTGAAAATGCACCTAAGGTATACACCATTACACATACTA
AATATATCAAATTATACGGACCTACCACCCCTAGTAACACGTATAATTTCTAA

> EhipOBP20
ATGTCAATTTTTTTTTTAGATCAAGTTGGACATATAGACC
AAGGAATATTCGTAGAAAATCATAATGTTATGTGTTATATTGCTTGTATTTACAAGTTGA
CTCAAGCAGTGAAAAATAACAAACTAAACTTGGATTTATTGATAAAACAAATCGATATCC
TCTATCCTACAGACTTGAAGGAAGAAGTAAAAAAGTCAGTGTACGCTTGTATTCACGTTC
AGGACAATTATGATGATATGTGCGAGGCAATATTCTACACTACTAAATGTTTTTATGAAT
TTGATCCAAAATATTTTATATTTGCATGA

>EhipOBP21
TTGAGTCCAAACTTTTCGGAATTCTCGACAAGGCAATTGAATGCAAGTT
TGGCGCGGTCACAGCCTAATTCACCGTCAGTCACATCTTCGTCATTTACTGAAGAGCAAG

CGGCGAAGAAATCGCGTACGTTCTGTAACTCCTCTTCATCTTCGATTATTTCTTTGACAT
TTTCTATGGCGGTCTCACGAGATAAGATACCTGTTTCATCGAAAATTCCAATTTTTTTGA
AAATGCATGAAGTAAAACAACCGGCATTTTCTCCGTCAGGGAACTTTCTGATTTTGAAAG
CGGCTATGTCATCGAGGCCCAACGGGTGCTCTTTGAAACAATCAGCGCCCACTTCTACGA
TTTTGGATTGTATTTGGGTCCTGTACTCATCACTGATGGCCTTCGCGTTTCCTAAAATTA
AGACAAATAAAGCAAATGAGACTAAAACTGACTTGAACATCTTGATTACACGTATTATGT
TGTTTATAATCGATCTAATCAATCAA

> EhipOBP22
ATGAAC
CAAATGGTCATAAAAATGACAAAGTCATTTATTTTGTTATTGACCATAGTACTACTATGG
TGTTCATTGGCAAAATCGCTAAATGTACGCAAAGACGATGTCAAGATGAATAAAGATGAG
GAAATGAATATGTCAACAATTATACCTGCAATGTCAATGGAAGAACAGAATAGAATAAAT
GACGTGGATATGATGGCAATTATGAATGAATGCAACGAGACGTTCCACATCGAAATGTCG
TACTTGGAGTCATTAAACGCAAGTGGCAGTTTTTTGGACGAAACGGACAAGACGCCAAAA
TGTTACATAAAGTGTATTTTGGAAAAATCTGGAGTATTCGATGAGGAGAAAGGTGTGTTT
GACCCTGCAAAGACTGCATCCGTGTTCGAAGGAGAAAGAGGTGGTCGTCCCATGGACGAT
ATTGAAGAAATGGCTGCAACCTGTACTGATAGAAAGGAGTCCTGCAAATGCGAGAGATCC
TACAATTATATGAAATGTCTCATGGAAATGGAAATAAAAAGATATGAAATGCATTAA

> EhipOBP23
ATGTCTTACACGACGCTGCTAATTTTTCTACTATTGA
TAACATTTGGTTACAGTGCTAAAGAAAAGCCGGAATTCAGTGAGGAAATCAAAGAAATAA
TCCAACATGTTCACAACGAGTGTGTCGGTAAAACCGGGGTCGCCGAGGAGGATATAACGA
ATTGCGAAAACGGCATATTTAAGGAGGACGTGAAATTGAAGTGCTATATGTTTTGCCTGC
TAGAGGAGGCGAGTCTCGTGGACGATGATGGCATTGTAGATTACGATATGATGGTTAGTT
TAATTCCAGAACAGTATTACGATAGAGTAACTAAAATGATATATTCTTGCAAACATCTGG
ATACGGAGGATAAAGACAAATGTCAAAGAGCATTCGATGTCCACAAATGTTCGTATGAAA
AAGATCCAAGCTTTTACTTTTTATTCTAA

> EhipOBP24
ATGAAGTTTCTGTTTTTGTGTTTTATTATAGCTGCTGGAAGTTTGG
ATGCCCATAATGTTCATCTTTCCCAGTCTCAAAAGGACAAGGTGCATCAATATACATTGC
AATGTATAACTGAGTCCGGTGTCAAGCCTGAGGTCATTGCCGAAGCGAAGAAGGGACATT
TCAATGACGATGAAGCTTTGAAGAAATTTATTTTATGTTTCTTCCAAAAATCTAGTATTC
TAAATGGAGAAGGAAAATTGGACGTGGAAGCGGCATTATCTAAGTTACCTTCTGATGTGG
ACAAGACAGCCGTGAAAAAAGTGCTCGAGGATTGCAAGAATAAAACTGGAAAAAGTACGG
CTGATACTGCGTTTGAAATATTCAAATGTTATTACAAAGGAACTCCGACTCATGTTATTT
TTAGTTAA

>EhipPBP1
ATGGCAGGACAACTGCAACTCTTTGTCGCTCTAGTGCTGTTT
GCTATATGTGTAAGTGAGATAGATAGTTCAGCAGAAACAATGAAGAATATATCTTCAGGG
TTCATTAAAGTTCTGGACGAATGCAAACAAGAGCTGAACTTAGGAGAACACATTCTAAAC
GACTTCTACCACTTTTGGAAAGAGGACTACTCTCTTCTGAACCGTGAAACAGGATGTGCC
ATTATATGTATGAGCAAGAAGTTGGACCTCTTAGATCCTGATGGAAATTTACACCATGGA
AACGCAAAGGAATTCGCCATGAAACACGGCGCTGAGGAAGAAGTAGCTTCAAAGATGATT
ACGCTAGTCCACGAATGTGAAAAACAGCATACCGGTGTTGAAGACGAATGCCTAAGGAAG
CTGGAAGTTGCCAAGTGCTTCCGTTCTGGCATTCACCAACTAAACTGGGCACCGAATATG
GATGTCATCGTTACTGAAGTACTTACTGAAATGTAA

>EhipPBP2
ATGTTA
ACGCAAACTAAAATAGTGGTTCTGGTGATTGTGTATTTGGCGATAGATTCGAGAGTTGAA
TCGTCGAAGGAGATAATGAAAGAGATAACTGTCAATTTCGGAAAAGCGCTGGCAGATTGT
AAAAGGGAGATGGAATTGCCAGATTCCATAGATGTGGATTTCTACAACTTCTGGAAAGAA
GATTACGAAGTATCTAATCGCTATACAGGCTGCGCCATCATATGTCTCTCTACTAAGTTA
GATTTGGTGGATCCTGATGGAGGCCTTCATCACGGGAATGCTCACGAATTTGCCAAAAAG
CATGGCGCTGATGATGGAATGGCAAAACAACTGATAGACATTATACACCAATGTGAGAAA
TCAACTCCACGGAACGATGATGGCTGTATCATGATGTTGGGAATAGCTAAATGCTTCAAG
GCAGAAATACATAAGCTCGATTGGGCACCGAGCATGGATCTGATGGTAGGAGAAGTGTTA
GCAGAAGTTTGA

>EhipPBP3
ATGGCGGCAGTCACAAAATGGCGGGCG
TTTGTGATATGTCTTACAGTTTTGGCTTTTGATTTGCACAAAGTGAATTCGTCGCAAGAT
GTTATGAAGAACTTAAGTAGCGGTTTTGGAAAAGTGTTGGAAAAATGCAAAAATGAGCTC
AACGTTGGTGATCACATTATGAAGGATTTCTACAATTATTGGCGCGAAGATTACGAGCTG
GTGAACAAGGACATGGGCTGCGTCATCATGTGCATGGCGACCAAATTGGACCTCATCACT
GATGAGATGAAAATGCATCATGGGAAAGCGCATGAGTTCGCCAAGAGCCATGGAGCTGAT
GACACCATGGCCAAACAATTAGTGGCAATTATCCATGAATGTGAGAACACTCATGCAGAC
ATCGGTGATGACTGCTCAAGAGTGCTGGAGATCTCTAAATGCTTCCGCTCCAAGATCCAT
GATCTAAAGTGGGCACCTCCCATGGAGGTGATCATCGAAGAGATTATGACTGAAATATAA

>EhipCSP1
ATGAGGAGTTTTATCGTAATCTGCT

TGCTGGCCTTGGTCGGCATTACTCTATCTAATCCAATAGAGACATACACGGATCGCTTTG
ACCACATAGATCTAGACGAAATTTTGGATAACAGGCGTTTGTTGATACCGTACATAAAGT
GTATGTTGGACCAGGGAAAATGTTCACCGGACGGAAAGGAAATCAAAAAGAATGTAGTGG
AAGCTCTTGAACATGATTGCCATAAATGTACTCCTACACAGAAGAAAGGCACAAAGAAAA
TAATCCGTCATCTGATCAATAAGGAGAATGACTACTGGATAGAGCTCAGCCATAAATATG
ATCCAGACCGTCACTTTGTAGGTAGATACGAAAATGAACTTACCGAACATTAA

>EhipCSP2
ATGCAGCTGTGTCGCGTCTTCTTATTCTGCTGTGTGGCGGCCGCCGCGGCGGCGCA
AGCGCAACGTCCGCAAGTCACGGACACGGCGCTGGAGGACGCGCTCAACGACAAACGCTT
CATTCAGCGACAACTCAAATGTGCGCTCGGTGAGGCGCCCTGTGATCCCATCGGCAAAAG
ACTCAAAACATTGGCGCCTCTAGTGCTACGCGGCGCGTGTCCACAGTGCTCGCCACAAGA
AACCAAGCAGATTCAGCGCACGCTGTCGTATGTCCAGCGAAATTATCCCCAGGAGTGGGC
AAAGATCGTCCGCCAATATGCTGGATAA
>EhipCSP3
ATGAATTGGTTATTTT
TAACAATAGGTTTAACCCTTGTGTGTCTGTCGATTGGTGAACAATACACGGACAGATATG
ACAATATAAACGTTCAAGAAGTATTGGACAACAAACGTTTGTTCAATGCTTATATGAAAT
GTATACTGGATAAAGGGTCTTGCACGCCTGAAGGGAGAGAACTGAAATCACACATAAAAG
ATGCTATACAGTCCTCGTGTTCAAAATGTACAGATAAACAGAAGCAAGGTGCCAGATTAG
TTGTCAATCATATCAGAGATAAGGAACCTAAATATTGGGAGGAGTTGAAGATTAAATATG
ATCCAGACGACCAATACAAGGAAATTTATGAAGCATTTCTTATAGCTAAAGATTAA

>EhipCSP4
ATGAAAATCTTCATCATATTGTTCGCCGTAATGGCGATTGCCATAGCTGCCGAAGA
AACCTACAGCTCGGAATATGACAATCTGGACGTGGAAGCCGTAGTCAACAACCCTCAAAC
CCTTCAGGCTTACTTTGGATGCTTCATCGATAGAGATAATTGCGAAAAAGAACCTGGCAA
CTTCAAAAAAGATCTATCAGAAGCTATCAAGACTGCTTGTGCTAAATGTACTCCCGCGCA
GAAACACATTCTCAAACGATTCACAGAAGGACTCAAGGAGAAATTCCCTCAAGATTACGA
GACATTCAAGCAGAAGTTCGATCCAGAAGGAAAATATTTTGTAGCTTTGGAACCTGTTCT
GGCTAAAGCTTAA

>EhipCSP5
ATGCCTGTGGATTGCCAACGGTCAACATCT
GGAGGAAAGGGCAACCTCGGTATGTTCATAGAAAGAGATCCAAAAATATGTGCAGCAGGC
TTACCGACAGCGCAGCCTGACTATCGAGTGGCAGATTCATTGCAGCATTATCTCCTAAAA
GATGAAGTAGATGCTCTAATGAGCGATACTATATTACCATCGCTAAAGCCCAAACCACTT
CCACCTCTACGAAAACCAATACCTCTTGATACGCGTTATGCAGGACCTTTTGGCTATGTT
ACTCAACTCGTTAATCCACCAACCAAAACTAAATTTCAAGTCCTTGTGGATGATCTAAAA
GACACATCGTATTCTTCTTATTGGAAGAAACCTCTTGGACAAACGCATGATTCTGTGCCT
ATGCTCCCAGAAGGATTTGATGCATTTGGAACTACTTTTGGCAAAAAGACGCCATTCCAT
GGGAGATTTTACGATGTCGTAATGCCAAAAATACCATATCCTGATAAAACACCTACGTCA

AAATTTCCTGGCGTGCAACTCGAACGTAATTATTGTGCTCCCCCTTACAATGGTGATCTA
ACATACGGATATAGAACTTATGTTGATAAACGAGGTTCTTATGCCAAATGTTGTTTAACC
GATAACAGAACCATAAAAGGAACAGCACAGCGAACTATCGTAAACAGTATACAAGCTAAA
TTTGAAGATGAGAAACAGCCACGTATTGGGACGGTTTTGGCTCCTAATGACAACATTAAT
TATGTACCAGATGGTCATGCTTTCGGTAAACTTAAACCCCCGTGTTCTCTCTCTGATTGT
CTTACTACGTGTGAAATAAATCCTGGAAAACATTTCTTTAGAAAATGCATCGCCCACCTT
AATTCGTTGCGTAAATATTTGTCTAAACGATTTTTACCGACGTTTTTCTATGGATTTTAT
TTGAACCTCAAATATTTAGATAAAAATCACACACACTGGTTGCCTAAAAATATAGTAATG
GATTTTTGCGGAACCAAAATGATACGATTTGACCCAGAATTTATTATACCCTTGCTTTCT
ATGTGGGAAGCATTCGATGGCTCTAATATTAAATATAAAACATTTGTACATATTATCAAT
TACAGAGAGCCATTGCCACAAATTCCTAAAATTCCTGATTTGCCTGCAGAATGTCTAGAT
TTTCGAACCACTTACACAGAGATGGTCAAAGCAGGACAAAAAAGTGACGCTCTATTTATG
GCTGGACTACCTTCAGGACGATATTTCGATCTAGATTACCCTATTACGCCCGAACGCTGT
TCCAAAGCTGACAGGATATGTCTTCCTCAAGAATCAAATATGAAGTCCTGCTTGAACCCG
AATGTTCTAACTCTTTTGCATGTTAATCACCGAGATATGTATGCAAAACGTGAACCACAT
ACTATTAAAAAAGTATTTGAAACATCTGGTGAAACATTTACTGAAGAAACATTCAACGAG
CTTTTCGAGGAAGCTAAAAAATATCATTCGCAAGGATGGGTTTGTTATGAAACATTTAGG
CGAGTTTTAGAAGAAAAATCTAAAACTAGAGAAATGAAATAG

>EhipCSP6
ATGAAGACGAT
CATAGCTTTTTGCGCATTGGTAGTTGTGGTGGTCGCCTTCCCCGGGGACACCTACGATCC
TCGGTACGACAATTTCAATGCGCAAGAATTAGCAGACAACCTTCGTCTGCTCAAGAGTTA
CGGCAAATGTTTCCTCGACGAGGGACCCTGCACTCCAGAAGGCGCTGACTTCAAAAAATC
CATCCCTGAGGCCCTCAGAACCACTTGCGCTAAGTGCACACCTAAACAGCGCGAATTGAT
CAGAATCGTTGTCCGCGCCTTCCAAACCAAGTTGCCTGATGTTTGGGAACAACTAGCCAA
GAAGGAAGATCCTAATGGAGAATACAAAGAATCTTTCGAGGCCTTCCTGAACCGCTCTGA
CTAA

>EhipCSP7
ATGCAGATATTACTCCTGAGTGTGATATGCGCGTGCGCGTTGTCC
CTGTGGCCAGTGTGGGCTGCGCCGGCGCCGCCAGTACCTCAAATGACGGATGCGCAGTTG
GAGAGGTCTCTGGCGGACCGCGCCACCATGCAGCGACACCTTCGCTGCGCACTCGGAGAG
GGACCCTGTGACCCTGTCAGTAGACGACTCAGAATATTGGCACCCCTGGTGCTCAGGGGT
GCATGTCCACAGTGCAGCCCACAAGAAGCCAGGCACATCCGCCGCACTCTCGCATACGTC
CAAAAGAATTATCCGTGGGAGTGGGCCAAAATCGTCCGACAATACGGCTGA

>EhipCSP8
ATGAAGTCGCTGCTCTTAATCGCTTTAGCTTGCCTCGTGACCGTGGCATGGTGCAGAC
CCGGCTCCACCTACACCGATAAATGGGACCACATCAACGTAGATGAGATTCTAGAATCAC
AACGGCTATTAAAAGCCTATGTAGATTGCCTCCTCGACAAAGGTCGTTGTAGCCCAGACG
GCAAAGCTCTGAAGGATACTCTCCCTGACGCTTTAGAACATGAATGCTCTAAATGTACAC

CAAAACAAAAAGAGGGTTCAGATAAAGTTATTAGGCATCTTGTGAACAAACGTCCGGAAT
TGTGGAAAGAGCTCGCGTCCAAGTATGATCCTGAAGGTAAATATCAAGAAAAATACGAGG
ATAAAATCAAAAATGTTAAAGAAAATTAA

>EhipCSP9
ATGTATGTAAACATATTAC
ATACAAAAATAGGGTGCACTGAAAAAGCTACGATGAAGTCTACAATTGCTTTGGCCGTGC
TGGTGCTGGTTGTAGCGACGACGACTCGTGCCGGTGGGGATTTTTACGACAGCAAGTACG
ACGAATATGATGTGGATGCCATTATTTCCAATAAGCGGTTGTTGGAGAACTACATAAATT
GTTTCCTTGGAAAGGGCAAATGTACTCCCGATGGCGCTAAGTTTAAAAAGATCCTTCCTG
AGGCCCTTGAATCTACGTGTGGCAGGTGTACACCGAAGCAACGAATCTTGGTTCGCAAAG
GCATTAGGGCAATACAGAAGCAATTGCCGGAATCGTGGGCCCAATTGGTAAAGACGTACG
ACCCGGAGGGAAAGTATAGGAACTCATTTGAGAAATTCCTTGCGGAAACCGATTGA

>EhipCSP10
ATGATTTCGTTAAAACATTGCATTATAT
CTAAACGCAAAGAAATTATAGTGAATATGAAGACTCAGCTAATATATCTGTTGGTTGTTT
ACCAAATGAAACATTATAATGCTGAAGAAACACAAACGTATACAACGAAATATGACAATA
TGAATCTCGATGAAATCTTGGCTAGTGATCGTTTGTTGACTGGTTACATAAATTGTTTGC
TAGATAAAGGACCTTGCACTCCCGATGGCAGAGAATTGAAAAGAACTTTGCCTGACGCGA
TAACTAACGATTGTCAAAAATGTAATACAAGACAACGTGAAGGTGCCGATCAGGTGATGC
ACTATATTATTGACCATAGAACAGAGGACTGGAAAGAACTTGAAGAGAAATACAATTCTG
ATGGAAGTTACAAAAGGAAATATTTAGAAAGTAAAACAGCAGAAAATAATACGTCTTCCG
AGCTCAAATCTACACATAATGCTGAAAAGGGTATTAATGATAATAAAGAATCAGATGAAA
AAGAAGAATAA

>EhipCSP11
ATGAAACTCTTTCTGATCGCTGTAGCTCTTTCCATGTACTGCATATTGGTTAATGCACAA
ACATATACTGATAAATATGACACTATCAACCTGGACGAAGTGCTTTCCAACAAGAGACTG
CTGGGCGCCTACATCAAATGTGTGCTGGAACAAGGACGTTGCACTCCCGAAGGGAAAGAA
TTGAAATCTCACATAACAGACGCTCTTCAGACAGGCTGTTCGAAATGCACGTCCAAGCAA
CGCCAGGGCATGAAACGAGTCATCAAACACCTCATTACTTACGAAGATGATTCATGGCAA
CTGCTGGTGGAGAAATACGACCCTCAAAGGATTTACTCCCATAAATACGAAAAGGAACTT
AATTCTTTGTAG

>EhipCSP12
ATGATTTTG
TGTCCAATCTCGTCAATGATGGTCATGGCTGTAGAGTACGAAGGAGGAATTTCGTTGGCT
GGACGTACTGGAACCTGTGTCGGTATGGTGTCTGATGCTGCGCCGACCCGCGTAGCCGGT
GTCGCCCTAGTTTGGATGACAGATCTAATAGTGTTAGATGCTCGTGGCGTCACTGTAGAC

GGTATGTTCGATGGAGATGAGCTGCTAACTAACAACACGCCGTCGTCGCCGAACCTATTA
AGTATAATAGGTGGTGCTGACGTAGACCCTGCTATTGGAACTTGTGGACTCGTCGGTTGA

>EhipCSP13
ATGAAGGTCCTGATTGTATTGGCTTG
TTTGGGCGTCGCTGCATATGCTGCTGAAAAATATCACTCGAAATATGATAATTTCGATGT
GGACACATTGATCTCCAATGATAGGCTCCTCAAGTCTTACATCAATTGCTTCCTTGACAA
AGGGCGGTGTACACCCGAAGGTTCCGATTTCAAAAAAACACTACCAGAAGCTGTGGAGAC
CGTTTGTGGTAAATGTACCGAAAAGCAAAAGGTGAACATAAAGAAGGTAATAAAGGCCAT
TCAGCAACGGCATCCGAAACAATGGGAAGAGCTTGTACAGAAAAATGATCCTTCAGGCAA
GCATCGCGCTGATTTCGACAAATTCATCCAATCGAGTTAA

>EhipCSP14
ATGATCATGAAGTGTGTTATTGCGCTGATTTGTGTGTTAGGGATGGTGATAGCAGATGAGAAATATACTG
ATAAATACGATAATATAAATTTGGATGAAATATTGGGAAACAAGCGCCTCCTGCAAGCGTATGTCAATTG
TGTGTTAGATAAAGGAAAATGTAGTCCTGAAGGAAAAGAATTAAGAGATAATGTCGAAGAAGCATTGCAG
ACAGGCTGTGCAAAATGCACAGAAGCACAAGACAAAGGAGCATCCAGAGTAATCGAACATTTGATTAAGA
ATGAAAAGGAAATATGGGGAGAATTAACAGCGAAGTACGATCCCGAGGGCAAGTACAGGAAGAAATATGA
AGATCTCGCAAAGTCAAAAGGCATAGAAATCCCAGAAAATTAA

>EhipCSP15
ATGAGGAATTGGCTGT
TGTGTTTATGCGCGCTGACGGTGGTGGTCTCCTGTTCCGGCCAACAGCAGCATTATAACC
GTTATGACAACTTCAATACTGACTCTATCATACAAAACGAACGCATCCTTCTAGCCTACT
ACAAGTGCGTCATGGACAAAGGACCCTGTACAAAAGACGGGAAGATATTTAAACGCGTAT
TACCAGAGACGTTGACAACCGCATGCAGTCGTTGCTCTTCAAAACAGAAGTTGGTAGTCC
GCAAAATGTTGCTAGGCATCAGGGCAAAAAGCGAACCGCGATTCCTCGAGCTGCTAGACA
AATACGATCCAGATCGCTCTAATAGGGATGCCTTATACAATTTCTTAGTCACTGGCAATT
AA

>EhipCSP16
ATGAAAACCTTCATTGTTGTCTGTTTGTTCGCTGTTGTTACTATCGCA
TCGGCTCGTCCTGATGACAAATATACTGACAAATATGATAAAATCAATCTGGATGAAATC
CTCGAAAACCGTCGTCTACTGATTCCTTACCTTAAGTGTATACTAGAACAGGGCAAGTGC
TCACCTGAAGGCAAGGAGCTCAAATCTCACATCCAAGAAGCTTTGGAAAACTATTGTGCT
AAGTGTACCGAAACTCAACGTAACGGCACACGTCGCGTGATCGGTCACCTGATCAACAAT
GAGGAAGAATACTGGGGCCAACTTAAAGCCAAATATGACCCAACCAATAAGTACGTGACC
AAGTATGAGGACGATCTCAGATCTGTTAAAGCTTAA

>EhipCSP17
ATGAA
GACCTTCATTGTCGTTTGTTTCTTCGCGCTGGTAGCTATTGCCACTGCTCTCCCTGGCGC
ACGCTACACCGATAGATATGACAACATAGACCTCGACGAAATCCTTGGCAACCCTCGTCT
GCTCAACCCTTATATTCTATGCCTTCTTGAGCAGGGAAAGTGCACCGCTGAAGGCAAAGA
ACTCAAATCGCATATCCATGAAAGTCTCGAGAACTATTGTGAAAATTGTACTGAAGCACA
GAGGAAAGGCGCGCGCCGTGTGATTGGTCATCTTATTAACAATGAACCTGAATCCTGGGA
AAAACTCAACGCCAAATATGATCCTGAACATAAATACACCAAGAAGTACGAGGACGAACT
TCGCACTATCAAGCAATAA

> EhipCSP18
ATGAAATCCATTTGCACAGTAATCTTATTTGTATTAAGTTTAGTGTATGCAGATTT
CTATAATTCTCGTTATGATGACTTTGATATACGACCCTTGCTGGAGAACGATAGAATTCT
TCTCAGCTACACCAAATGTTTCCTCGACCAGGGACCTTGTACGCCAGATGCTAAAGATTT
CAAGAAAGTTATTCCAGAAGCGTTGGAGACGTCATGTGGAAAATGTACAGTAAAGCAGAA
ACAACTAATTAGAAAAGTAATCAAGGCAGTAATGAAGCAACACTCTGACGCTTGGAATCA
ACTTGTCGAGAAATACGACAAAGAGAGAAAATATAGGGATTCCTTTAATAAATTCTTAGA
AGAAGATTAG

>EhipPR1
ATGTTTTTCTTTAAGGAGAATGATATGAACACAATAAAGAGGCCT
CAAGATCTCCGATATATGAAGCAACTACAGTTCTCTCTAAATATTGTGAGTGCTTGGCCT
CATAAGGAAACCGGCGATGCTGGGTCCAAATTTGTGTTTTGGTGGAGATTATATTATGCA
TTTGTGGAAGGATTTTTTTGGTTTCTTGGTGCAGCTTATTTAAAAAACAACTACGGGAAA
ATAAGTTTTTTCGAATTCGGCCACACCTTAATAACTCATTTCATGAACACCATTGCTTGT
CAAAGATTGACTTTGCCGTTTATGAAGAAGTATCGTGATTTCATTGGTCTGTTTGTAAAG
CAATTTCATCTTTTCCATTACAAAGATAAATCTGATTATGCCATGAAGATTTATCTACGA
GTGTATAAATTATCAGATTTCTTCTCTATGTACCTTCACATATTAATGTATATAGGCATA
GTCTTATTTAATGGTACACCTATTTACAAAAATATATTATCCAATGCATACAGCTCGAAT
AAACCAGAGAATGTAACATTCCAACATTCAACATATTTCGAATTACCCATCGACTATAAA
CACAGTTTGACTGGATATGTTCCTCTGTTCTGTTTTAATTGGTACATAACTTTCATATGC
GCCTCTTTCTTCTGCATGTTTGATTTGTTGTTGACCGTGATAGTGTTAAATGTTTATGGT
CATCTGAAGATTCTTGTATACCATTTAGAACATTTCATGACACCGTCAACGAATTCGACA
TCTCATAAACAGAAAAATGTGTTCGATATAATGCAATTCTCTGAAGAAGAAATGAAAACA
GTAACCATTAAATTAAAAGAGGTTATATCCCATCAGCGTCTTATAACAGATTTCATACAG
AAAATGTCGGATATTTTTGGGCCGATGGTATGCTTAAACCTCATGTATCAACAAGTCAGC
GCATGTATTTTACTGTTGGAATGCTCGCAAATGGATTTACTAGCCTTGTTGTCCTATGGC
CCGCTTACATTTTTTGTATTTCAAGAGTTGATTCAGTTATCCGTTGTGTTTGAACTTATT
GGAGCGACGAGTGACGACTTAATAGACGCCGTATATAGCGTGCCCTGGGAATGTATGGAT
ACTAAGAATAGAAAAATATTGTACACTATAATGATAAAGTCACAAATGACGACAAAGTTT
AAAGCTATGGGTATGGTGGATGTTGGCGTTAAAACTATGGCGGCAATATTGAAGACGATA
ATTTCCTACTTCGTTATGCTTCGAACAGTAGCTTTACAGAATTAA

>EhipOR1
ATGTTCTATTTTACTGAAATAGTGGTCACCACTAAAGTTTTTATGGTTCTGGGTT
TCCGAAGCAAACTACTTGATATTATGAATCATCTGGATTGTGAGGAATTCAAAGCAACAG
ATGAGAACAGTAGAAAAATAATCGACAAACATGTGTATTATTATAAAACTTATTGGAAAA
TATTTTCGACGTTATCTCACTTGTCCTATTTTTTCTTAGTTTTACTACCCATAATTATAG
CTAAATTAATGGGAACAAATCTAGAATTGCCTATTTGTAAATATTATTTTTTTGATGATA
AGTTGCGAAATCGTTATTTTTATTACTTATTTATATACCAATCTATTGGAATGTATGCAC
AAATGACATACAATGTTAATGCTGATACACTAATGTCTGGCTTGATATTAATGGCTGTAA
CACAATTAAAGGTTCTGAATTATAAATTAAGTAATTTAAAAGTAACAGCAGAACATTCCA
AATTAAGCCTAGAAATTCAAGATAATATACAATATCAAAAGCTCAATGACTGGTTGAAAC
ATCATTATTTAATAACAACATTTTGTACGAAAATTCAAAATTTGATAAACGTAACTATGC
TCATTCAGTTTGGAATGTCAGCGGCCACTATATGTGTTTCCTTATGTGGATTTCTTATGA
TGTCATCCACTGGGACGCTGATGTTTGTATCTTCGTATTTGTTCGTTATGATAGTAGAAA
TTTTCGTACCAGCATGGATGGGGACTCAACTCAGCTATGAGAGTCGAGAATCAGTATTTG
CTATTTATGACAGCGAATGGATACCAAGGTCGGAAAAATTTAAAAGAAACATGAGATTAT
TTGTGGAATGCACAAATGTTCCTATAATATTGAGAGGCGTCAAAATGTTTCCACTGTCTC
TTGAAACTTTCACTTCGATAATGAAAACGGCGTATTCATTTTTTACATTGATTCGTAATG
TTCAAGATCGTGAAAATGGAAGAATCTCTTAA

>EhipOR2
ATGTCGCCGATAAAACAGTCAGAAAGTTT
TAAGTTTAATTTTGTCTTGTTAAAATTTTTTGGAATATGGCCAGGATGTACAACAAACAA
ATACTATAAATATTATTCTATCGTATATTTTGTCACCATGCTTTTGATTTACAATATCCT
CTTAACTGTCAACTTATTTTACACGCCTCAAAAAGTAGAGCTGCTAATTCGAGAAGTAAT
ATTTATTTTTACCGAAGTGGTGGTCACGACTAAAGTTTTGATGATTCTCTTGATGAGAGA
GAAACTTGTCGTCCTATTTGATATGTTAGATGATGACATTTTTAACGGAGAAGATGCTAT
CGGTAGAGACATTGTACTAAAATATAACTCGTATTACAAAAGCTATTGGAAAATGTTTAC
AGTGTTATCGTTATTTGCTTATTTTTCACAAGTGTTTTTACCAATTATAATTTATTTTAT
ATTTAACGTAAGTAATCCAGAATTGCCTATTTGCAAATATTATTTTTTGAGTAATCAAGT
CCGCGAGAAATATTTCTGGATTTTCCTTCTGTATCAATCTTTTGGAATGTTTGGACATTT
GATATATAATGTCAATATAGATACGTTGATGTCTGGATTGATTTTGATGGCGGTGGCACA
ACTGAAGTTACTTAACCATAATTTGAGAAACGTTAAATTCCAAAAAAATAAATCAAAATT
GAAAGTACAAATTCAAGAAAACATACAAATGACAAGGCTGAACCAGTGTCTGAAGCATTA
TGATGTATTATTGAAATATTTTTATAAAGTTCAAGAATTTATCAGCGTAACTATGTTCAT
TCAATTCGGAGTGGCTTCAGCAATCATTTGTGTTGTAATGTGTGGACTACTTTTGTCTTC
GACAACTGAAACTATGATGTTTATGGTGTCGTATTTATTTGCTATGACATTGGAAATATT
TGTGCCGACGTGGTTAGGAACGCAGCTAAGTTACGAAAGCCAGGAGTTAGTGTTTGCCGC
TTACAATAGCGAATGGATACCGAGGTCGGAAAGTTTTAAACGAAGCCTAAGGCTTTTTAT
GGAACATGCTAATGCCCCGCTCACCCTAACAGGCCTGAAATTATTCCCTTTGTGTCTGGG
GACTTTTATTTCGATTATGAAGACGGCTTATTCATTCTTTACTCTCGTACGAAATGTTCA
AGATAGTCAAGGTGAAGCAATTTAG

>EhipOR3

ATGGACGATATGGAATTGAAAAAAACACATCCTCAAAAATATTACTTGAAAT
TCATTTGTAATTCTTTATACGTTTTAGGATACGGAAGTTGTTGGTACGAGGAGACTCCTC
GAACCAATTTCCACAAAATCTTCTACAAAATATGGGCTGGAATTGCAAATTTCTTTGTAG
TTATCATAGTAATAAATGAGATAATGGCAAATTTCAGACCAAATTTGACAGCGAAAGAGC
AGAATGATTTGGTACAATTCACATTTGGGCATAGTCTTATTATAGCGAAGATAGTAACTA
TGTATTATCAAAGGGATCGCATCAAGGCTGTGCTAAAAAAGCTTCTAGAAGAGAACAGGA
CAATATTCATTTCAGCAGACATAGATAAATCATCGGTAAAGAAAGTGAAGATATACTGTA
TAGTTTTGGTTACTACAGTATATTTAACTATGCTCTCTGCATATATTGACGGTTTCAGAG
TTCATTTTAATGAAGGCATACCTATTCGAGGTGAGATAAGTTACTACCCAACGCCTTTGG
ATTCTGGCATACTCGTGAACATACTGCGATTCATTATGGAGTTTCACTGGTTGTACATCG
TGACTGTCATGAATTTGATCGATTGCATGTCATACTGCACACTCATCTTCTTATCGTCCC
AGTTTAAATTAACACAAACTTATTACAATCTTTTGAGAAAGAAATATACAAAGAATTCTA
ATAAGAATACTTGTAGTGTTCTAGGAGAAGAATATAAAAAGGATTTTCTAATTGGCATCA
GGCTGCATGAAAATGCTTTGTGGTGTGCCCATCATGTCCAGGCCTCCTTGGGATATATGT
ACAGTAGTCAAATATGTCAGAGCATAATTCTCATTGTCATGTGTCTTGTTAAATTTGTTA
CGTCAGCTCGTAATATGACGGTATTGCTGGCTAATATGACGTACTTATCGGCTATGACAG
TAATGACTGGAGCATATATGACGGCTGGAGGAGATATTACTTATGAGGCATCGCTAGTAT
CGACGTCAATGTTCCTCAGCGGTTGGGATCTGGTTGTGTTTGACAAGGAGCTGCGAACGC
TGGCGGTGGTGGCCATCCAACGGAGCCAAGCACCCGTATACATGACCGCTTTTGGAGTCA
TCATTTTATCCTACGACAATTTAATTATGGTGTTGAGATCATCATATTCGTTCTTTGCCG
TTATGTATTAA

>EhipOR4
ATGATTGATGTTGAGAATCTCTATCTAAACAGAGCTAAATTCGTAATGAAAATTTTAGG
AGTATGGATACCATCTGAAAGCGAATTAATGATAAGCAAAATGTACAGAATTTTCATGAT
GAGTTTGCAATATTTATTTCTTTTATTTCAAACTATTTACATAGTGCAAATCTGGGGTGA
CCTCGAGGCTGTGTCTCAGGCATCATACCTGTTTTTCACTGAAGCCTGCCTATGTTTTAA
AGTGACAATCTTTCACATAAATATGGATATGCTTAAAGAATTATTAAAAGAAATGAATTC
TGACGTCTTCAAGCCGTTATCTCTTAAACACGAGAAAATTTTAAAATTACAGGCAAAAAG
GATTAAGAGGCTATTGCTAGGTTTCATGATTAGTTCCCAAGTTACATGCGGATTGTGGGC
TTTGAAACCGTTATTTGACAATGCTGGTACTCGAGCATTTCCATTTGACATGTGGATGCC
AATGAGTCCGAAGTTCTCACCGCAATATGAGATAGGCTACGCGTACCAGTTCCTCACCGT
CTGTATGAGCGCCTATATGTACTTCGGAGTCGACAGTGTAGCCCTGTCCATGGTCATATT
CGGGTGTGCCCAAATCGACATCGTTAAGGATAAAATTTTGAGTATCAAACCTGTATCAAA
CGCGCGTGGTGACGAAAGAAAGAAAATACTATCAGAAAACAATAAAAAACTAATCGAATG
CGTCGTGCAGCACCAGGCTATAGTGACATTCACTGACCTAGTGGAGAATGCGTACCATTC
ATACCTGTTGTTTCAACTCAGCGGTAGCGTTGGACTCATCTGCATGTCAGCCCTACTTAT
TTTGATTGTGGACTGGTATAGCATCCAGTTTCTGTCAATAGTAACATACTTGTCGGTCAT
GATAAGTCAGCTATTCGTGTGCTGCTGGTGTGGACATGAACTCACTGCTTCTAGTGAAGA
TCTACATGCCGTGTTGTTCCAAAGTATGTGGTATGAGCAGGACGTGAAATTTAAACGAGC
GCTTTGTTTCGTAATGATACGCACGAGTCGACCAATGGTATTGCGCGCGGGACACTACAT
CAGTCTATCTAGACAGACTTTCGTGTCGATTCTGCGTATGTCATATTCCTACTTTGCAGT
GCTGAATCAAACACAATCTACATAA

>EhipOR5
ATGTCTAAGAGCACAAAGTCTTTACTGAGGGCTTTTTGTAAGTATG
TTTATTACGCCGGAGCCGGAAATTGTTGGTACGAGGATACATACCGTGAAACATATCTCT
ACAAAGCATACGCCCTTATTTCTTTTTCTATTTACACCACAATGATTTTTCTTGAAAATC
TCGCTGCTTGGTTCGGTTCTTTCCCAGAAGTAGAAAAAAATTCAGCTGTCATGTTCGCCG
CCATCCACAATATCGTTCTCCCTAAAATGTTTCTCCTGTTATATCATAAGAAGTCTATAA
GGAAATTGAACTACGAAATGGCTACTGTAGGGGAAAAAATCGAAGAGAAATATGTCATGG
AGAGACAGGCCAGAAAAGCGAAAATTGGGATTATACTATACGTGATATCGGTTTACCTTT
CGCTAGGCGCGTATGGCGTGGAAAGTACCCGGAAAGTGATAGTCGAAGGTGCACCCTTTT
ACACTGTGGTGACATATTTGCCTCAATATGACGACAGTACCATAGTGGCATCAATATTCC
GCGTAGTTTTTTACATCACTTGGTTATATATGATGTTACCGATGATGTCAGCTGATTGTA
TGCCGATTACACATCTGATTACTATGACGTATAAGTTCATTACACTGCGTCATCATTATC
GTAGAATCAGAGAAGAGTTTGATAAAGATCTCCTAACGATGGATAAGAGACGGGCCGCCG
AGAAGCTAAGAGCCGGTTGCTTAGAAGGTATATTGATGCATCAGAAACTAATGTTTTTGG
CTGACGAAATAAATCGTATTTTTGGAATAATAATGTCGCTTCAAGTTTGCGAAAGTTCCG
CAGTAGCAGTTTTGCTTTTATTGCGTTTAGCGCTTTCACCACATTTGGATTTAACGAATG
CTTTTATGACGTACACTTTTGTGGGATCTCTGTTCTTATTGCTGGCATTAAATCTTTGGA
ATGCAGGAGAAATTACTTATCAGGCATCACTGCTTTCACATGCCATGTTCTATTGCGGCT
GGAATCTGTGTGACATGGACAAACAATCTCATCACGACATCCGACGGCTCGTGTTAATTG
GTTGTGCGCAAGCGCAGAAACCTCTCATACTTAAAGCTTTCGGCATACAGGACTTGTCCT
ATGAGACTTTTGTTTCGGTGGCGAGAATGACATACTCAATTTTTGCAGTATTTTATCAGA
GGGGAGAGCAAAATTAA

>EhipOR6
ATGGATAAAAAAG
AAAATATTCGTATTTTTACTGGAAATCTTTTAAATTTCCTTGAAATTCCAAATCATCCAT
GCATAGGTCCCCATCTCAAACTTTTAGGTCTTACGGGTCTCTGGCACCCAAATAAAAACT
CACCCGTCACAAAGTTCAAGCGAATCCTTTTTTATGTCACTGTAACGTTCTTCTGCAGTC
AATACATTAAATGTCTTTTCAACATTGATGTTATCTCTCTCGCCCTTATTCTGCAATATG
CTCCATTTCATATGGGCATAGTAAAGTCATGCTACTTTCAAAGACATTACAAAAAGTGGG
AAAGACTCATCATTTACATGTCATCAGTAGAATGTGCTCAGTTAGCTGACAGACGAACAG
ACCTTATCCCGATAATGAACGGATACATAAAGAAAAGTCGACGCATCACCTATTTCTTCT
GGGCCTTAGCTTTTTTCTCGAATTTCGCTATTTTCTCAGAACCTTACCAGAAGAATCATG
TTATGGAGAATAGCACTGCTGTCTATACGAAGATATTCGATGGTATCGCGCCTTTTAATC
AAGAAAAACCACCAGGTTACTACGTATCCATGGTTTTGCAAACCATTTTTGGTCATATAG
TGAGTGCTTACGTGGTTGCTTGGGACACTTTAGTTGTTTCCATCATGGTATTCTTCAGTG
GACAGCTGAAAGTGTCTCGTTTGTACTTCGTTCAAATTATCGATGTGAACAGTATTGAGA
AGAGCCATCAAAATATTGTAAAATGTCATTATTTCTACACTACTTTGGTTAGATACCAAA
AGATGTTCAATTCGTTGATATCGCCTGTGATGTTTATATATTTAATAATTATCTCTGTCA
ATCTTGGTGTTTGCATCATTGAAATCGCTCAGTTACAAGACGACATAGTCACGCTCGTCT
CGAGTTGTTTATTCGTATTGGCTTGCGTTATCCAATTATTAATATTCTATTGGTACGCCA
ATGAGGTCACTGAAGTGAATAATCTAGTAGGTTACGGGATATTTGAAAGCGAGTGGATGC
AGTTAGATAAAAGCCTGCAGAAAGAAATAGCTTTACTGGGCCTAATAACAACAAAAAAAA
TAGTTTTCAAAGCCGGGCCTTTGAATGAAATGTCGCTATCAACTTTTGTGGGGATTTTAC

GAACAAGTTACAGTTTTTACACTTTGTTAAGTAAAACTAAAAATTAG

>EhipOR7
ATGCAAACGCAGACCTGCCTTCATGTT
TGGGCTCCTTTCGACTACAGATACGATTTTCATAAATGGCTAATTGTTCATATTATTATA
AACGTATACGTAGTTGCACACGGATGTGGAATATTAGGTATTTTCGATGTCGTATTTTAC
ATCATTGTGTTCCATTTGATTGGTCATATTAAAGTTTTGAAATATAAAATCAAAACTCAA
TTTGAGGGTGATCTGGATGATGAGGAGGTGAAAAAACGACTCGTAAACGTGATAAAGTAT
CATGCGTTTATTATAAAGTTTTTCAAAGATGTGGAAGCTGCTTTTGGTATTAACGTTTCG
GGGAATTATCTTAATAATTTAATTGCGGACAGCCTTATGTTATACAATTTAATGATTATA
GCTCAGGATAAAGGTACGGTAATCATATTTGTCGTGATGACAACAGTTTGTATTACCGAA
CTAATTTTGATGTCATTTATATTGGAAGAAGTCCGTATACAGAGCGATGATCTACCAGAA
TTAATATATTTCATGCCATGGGAAAACTGGTCACTGAATAATAAAAAAATGTTAGTGCTC
ATTCTTCTACGAATACAACCAGAGCTGGCCTTTGTGGCTGCCGGTGGCCTCAGAGCTGGA
GTACGGCCTATGACATCAATAATAAAATCCACCTTTTCTTACTACGTCATGTTGAAATCA
AGCATGAGGGAGTGA

>EhipOR8
ATGATTTTTATA
AATTTACTCACAATTACTATCACTATATGCTTCTCCGGATTCCTTGTCAAATTCACTCGC
CGCCCTTTAGATATGGTAAATTTTTTCGTCGTTGCTGTGGCATGCATACTTTGCATATTT
CAGTTATGCTATTACGGCGAAATGCTTTCGAGAGCTAGTGTAGAAATAGCAGATTCAGCT
TATGAAAGTTTGTGGTATAAATGTAATACAAGCCATCAGAAGGCATTGATGTTTATTATT
TCAAGAGCTCAAAAACCTTGTAGCCTGACCTCACTGAAATATGCACCGATTACTCTAAAT
ACGTTTAGTAAGGTAATGGGTACAACTTGGTCGTACTATTCACTCGTTAGGACTGTTTAT
GAAAGGGAATAA

>EhipOR9
A
TGATTGGTAAACATTTCAAAAGATCATTTCAAACGACTAATCTGTTTTTCAAATTAATGG
GCATTCGATTTAGGTGTAATAAAAATTTTTTTGATCACATTCAATGCTATTGGCTTTTCT
ATTTCAACATTTTATGGCTGAACAGGGATTTGTTAGGTGAAATATTGTTTGTGGTTTTCG
GTGTGATGAGTGGAGAAAGTTTCGTGGACCTCACGTATATGTTACCTTGTATCGCTATGT
GTCTGTTGGGTAACATGAAAACATGTCTTTTAATCATATATTCCAAGGAAGTCAAAGATC
TGATTGATACGTTGAAGAATATGAGTTTTCATGACGATGATATGTCTGATGAAAGAAGCA
GTTATGTTGATGATAGCAATGATTATGTTTATGACAGAGTTGATTATGTTGATGGAACAT
GCGCTGCTAATGTTGATGAAACTAGAGATTGTGTTGATGAAATATTTAAAAAGACATTGC
CTTTTATGTATTCGGCGATAAAAGTTCAGAAAATATGCAATGTTTTAGTTGTAACCAATT
TCGGATTGAATCCTATGTTTTTGATGGTTTTGAACTATCATAGCACTGGTCAATTTGGAC
TGTTCATGCCTTTTCATATTTGGTATCCGTTCAATGCATTCAATTATTGGATTTTCCCTT
TCGTATACATCCATCAGGTATATTCAGCATATTTAGCAGCATTTACTGTATACGGACCAG

ACACACTATTCTACGTCTGCAGCACTTTCATAGCCATACAGTTCCGTCTCCTGCAGAATA
ATTTGGAAGCTATTATACCTTTGAATTATACATCTAAAATTGAAGAAGTTAATGAATTTA
ATATGACAATTAAAAGAATAGTCAAATGGCATCTCGAGCTGATACGTTGTGTTCAATTAT
TAGAAAAAATATTTTCGAAATCAACTTTGTTCAACGCTATTACGAGCTCATTTCTTATAT
GTCTTACTGGATTTAATGTCACGGCTATCGACAATGTGCCCTTTATGCTATCTTTCATTT
CTTTCCTTCTAGTGACTTTCTTGCAAATATATTTCTTCTGTTGCTTTGGAGATATGATAA
TGCAATCTAGCGTCGAAGTAGCCGAAGCTGTATATAATAGCCGCTGGTATATGGTAGAAA
CGTCTCTTGCTAGAGAATTGATGATTATTTTAATCAAAGCACAAATCCCTTGTAAAATAA
CAGCATTTGGATTCGCAGACATTAATCTAAGGGCTTTTATGAGGATACTGAGCACATCTT
GGTCATATTTTACTCTTCTGAAAACTATGTACAGTACATAG

>EhipOR10
ATGGAACATATGAAAGATTTCCCCTTTGAATACGTGAAACCATTTCTCCCATGCTTCGAT
CTATTAAAGAGATCTAACGTTAGAATTTTCACATATAACAATAGCTCATTTGTAAGTGTG
TATTGGCATACGTTCTTTCTTGTACCGTGCGCAATTGGTTTCTACGTTTCTTTATCGGCA
AATATGTACAAAGTGTTTTCGGGAGATTTAGATATAGTTGAATTAGCATATATAGTGCCG
GTATATTTAGTTTCTACTCAAGGAATACTCAAAACAATGACTCTGACGATGAACAAATCT
GAGATAAACGCTTTAATTAATGATCTCGGCACTATATGGAGAAGAACGGGCCTGTCTCAA
GAACAAATTACTAAAAAGGATTCACTTCTAAAAAGATTGAATTTCTGCAATTCAGTTTTT
TGCCAGTTGAACATATTCGGTTCTTGGCAATATATTTTGGCCCCATTACTAGAGACTATA
TTTCGCATATTTATTTTGAAGCAAGAGACGGATTTTCTGTTACCATTCGCTTGCTTTTAC
CCATTTGATCCTACCGACAATTGGATTATATATATCGTAATATACTTATTTGAAGGTTAT
ACTATGTTCCACATTGTATATGTATATTTGGGTGCTGAAATCTTAATGATTACACTGTGC
GGCCACCTTAGTATCGAACTTGTGCTGTTACGAGAGAACTTGCTTCGCGTCAAAACTACG
TCAAACAAAAATGGGACACGTGATGTCACATCCGGCTTCAATAATAAAGCGCCCCTTGAT
GAGGGCGCGGAATACGGAATAGATGATATTGTTAGAAGACATCAGAAGTTGATAAGATTA
TGTCAACGTTTAGATAATATATTTAACACAATGATCTTCAGAAATCTACTTACTGCTACA
ATTACTATTTGCTTCTTTGGATTTGTTGCTAAATTCTCTCGGGGTCCCACGGACATGATA
AACAATTTCATTGGCGTCGTGGCATCCATAATACCAATATTTAATTTGTGTTACTATGGC
GAAATGCTTGCAGAAGCTAGTGTTAGCATAGCAAGTGCTTCATATGAAAGTTTATGGTAC
GAATGCAGTAGTAGTCATCAGAAAGCATTAGTGATTATTATGTTAAGGGCTCAGAGATCA
TGCTGTTTAACATCACTAAAGTATGCACCGATTACTTTGAATACTTTTAGTGCGGTATTA
AGTACTACATGGTCATACTTTTCAATTGCAAAGAGTATGTACGAAGAAGATTAG

>EhipOR11
ATGATGTCACTGTTGTCTTTGGG
CGAACAGACCTGGTGGGGTTACAAACCCTACGGCACTATAGTTTATATCAACAGCTCGCT
TGTACCAATTTTCGGACCACTTAGCTTAACGTGCCAGATAATATACTTATATCAGAATTT
CAGAAATCTACCATTAAATGTGCTTGGTACAATTTTTTCAATGCTACCTATGACAGCTTT
GGTTAATATAAAAATACGAGTGCACAAAACGAAAAAAATATGAAAATCTTATGAAGTCATT
CATAACCAATATTCACTTACACAATTATAAGGAAGAAGATAGTGTTGTTAAAGCAACCAT
CATTAAAATAGAACGTTACAGTCGTTCGATGGCTTATTGCGTCATATTCCTTGTCAACTT
CAGCTGGATGTTATGGTCCGTGATACCATTTGTCAACAATTTGAACAACAAAGAAGCTAT
TCATAACAAAACTATGCTTATGCAATCGTGCCTTTACATGTGGATGCCTATCGAATACGA

ATATGATTTCAAGAAATGGCTTATCACCCATTTTATTAACATATATTTAATGGGTGTTGG
GTGCGCTCTCTTAGCTATACCCGATATCATCAACTATACAATGGCAATGCATTTAATTGG
ACACGTCATACTTCTGAAACACAAAATAGTATCAAGTTTCCCGACTGAACTAAATGACAA
AGAAGTGAAACAGAAATTAAAAGATCTGGTCGAGTATCACTGTTTTATTCTCAAATTATT
TAAAGATGTCGAATCGGTATTTGGTATAAATATTTCTACAAATTATTTTATCAATTTACT
AATAGATAGTCTTCTACTATATCAATTAATGAACCAGGAAAAAGGAGACAAAACTTCACT
GTTATTTGGTATAACCATTGTAATTTGCATGGGAGGACTGATAATAATGTCTTTCATCTT
AGAGGAAATACGTAAGCAGAGTGACGATCTACCCGAATCTCTATATGGCATATCGTGGGA
AAACTGGAATGTTTCTAATCAAAAATCACTTCTTATAATCTTGGCGCAGCTGCAGCCCGA
GTTGGCGTTTGTGGGTGCAGGAGGACTGCGCACTGGAGTAACACCCATGGTGGCTATAAT
AAAATCTACTATTTCTTATTACCTCATGTTGAAGTCAACGATTTAA

>EhipOR12
ATGAAAAATCACAATATTCTAAAAAATCTTTG
CAATGTAGTATTCGTGGCTGGTTCCGGGAATTTCTGGCTCAAAGAAAATCACATAGGAGA
CGATAATAGTTTTCTTTACAGATTTTATCGTTTCGTACTGTTTTCTATATATGGTTTCAT
GACTATTTTGGAGATATTAGCTGCTGTAATCGGGGATTTTCCAGACGATGAGCAAAGTGA
TGCTGTTACTTTCGCTGTAAGTCACACCATAGTGATGATAAAAATATTCTCAGTTATTTA
TCGTAAGGAACTAGTGAAAACGCTGAACAGAAATATGGTGAGGGTATGTGAAATATATGA
GACGAAGACGTTGATGGAAGAACAGTACCGCATCATGAAGATAAATGTGATTGCTTATTT
TGTGACGGTGTACGGGTCAGCCGCTTTTTTTATCATCGCAGGTGCGCGGAAGATGCGCGA
AGGTTCCCATTTCATCACCATTGTTACATATTGGCCAGGACACGACGATGACACAGGCAT
AGCTACCGTTTTTCGAATTTTTACTACTGTGGTGCTCTGTGTGATGATGGTCACGATGGT
ATCCATAGATTCATTTGCTATGGTGTATCTAATTATGTACAAGTACAAGTTCATTACACT
GAGGCATTATTTTGAAGAGTTAAGAAAAGAATTTGATGAAGCAAGTAAGTCGAGCTTGGA
ATTGGCGTCGGACAAATTGACACAAGGCTTAGTTGATGGAATTGAAATGCATAAAAAACT
TTTAAGATTGTCGAGAGATATCGACCGATCATTTGGAACAGTAATGGCGTTACAAGTCTG
TCTGAGTTCTGGATCAGCCGTCTCGTTGCTACTTCATCTTGCGCTCTCAAAAGAATTGAC
GTTTGTTGTCACCATGAAAATACTATTTTTCGGAGTTGCTCTATTCTTTCTATTAGCTCT
TTTTGTTTGCAACGCTGGTGAAATTACTTATCAGGCATCATTACTAGCGGATTCTATATT
TTACTGTGGTTGGTACGCATGCCCGTCACAGTCAGCACCACGTCGCAATATTCGACAGTT
GGTGTTATTGGCTGTAGCGCAAGCGCAGCGACCTATCGTTATGAAGGCATTTAACATGCT
CGAGCTTACATATGGTACATTTATATCGGTCGTGAGAGGAACATATTCCGTATTTACATT
GATATATGCACAGAACACCTAG

>EhipOR13
ATGTTTGCTCC
CAGAAAAGAACGAAGTATCTTTTTAAACGAGATCCATTTATTGAGTTTTTATCTATCTAA
ATTATTTCTGTTTCCATTTTTTGGTAAAACGAAATTTAAACAATTCGGTTACTATTCGAC
TTATTTCTTAATAGTTTTTACATCGTTTCAGTTATGCTTTACGCTTCTTTTAAGTGGCTT
GAATGATTTTATTGAAATAATTAACATTTCCCCTAACCTAGGTGTCTGTGTGATGTGTGC
TATAAAATACGCCAAGGTAAATTCTAATAGAGCACTTTACTACGATATATTCGAGCATTT
CCGTGAAGATTTGTGGAAAACTATATCAGAATATTCAGCTGAAGATCTGAAAACAATTAC

AAAATATACGAAAATAATTAAATTAATAAACAGAACATTGATATTGTATATTTCTTTGCC
GTTAATTGCAATCGTCAATATATGTCCATGGATTCTCATGGTTTACGAAAACAAAGTCGG
TAAAGAACAAAAATTATTATTGCCTTTCGACGGCTGGTATCCTTTTGACAAAGTGAATTG
GTATTTCGTGGCTTATATTTGGGAGAGCTTAATGACTGGCCTCATTATCTTCGTGTATGC
TATTACAGACGCGCTTAACATATCATTTGTTGCTTGCATATGTATGGAATTAAAATTATT
AGGAAACAGTCTCGAAAATCTTATTAATCCTGAAGATATCGAAAATATTACAAGACTTAA
AAACATTAGCAGAACGCATGAAAATATTAAAAGAAAATTGAATGTTACCATAAAAAGACA
TACATTTTTAGCGAAGATATCCTCAGAGTTAAATATAACTTTAGGTGATCTAATGCTTGT
GAATTACACCTTCGGCTCTTTATTCATCTGTTTAACAGCTTTCACGTTTACGGTAGTTGA
TGATTTATACAAATCTTTGCGTTATTTCTTCTTTTTCGTTGCACTAATTGTAGCGATATT
GGATCAATGTATAATGGGGCAGTCTTTGAGCGATAATAGTGAACAATTAGCTGAAGCAAT
ACATGCCTCGAATTGGTTGTATGCTGATCAACAAACGAAACGCACGTTATTAATGTTATT
AATGCGAACACAAAAACCATTTCAACTAACAGCAAATGGATATTTGGTTATGAATCTTGA
TACTTTTACTCGGATTTGCAGTTCCTCATACCAATTCTTCAACTTGCTTCGGACAATTTA
TCAACCTTAA

>EhipOR14
ATGCCACATTTAAAATCAAGTGTAGAAGAAGAATATATCAAGACACCACCGAA
AGAGCAGTTATTTTACCAGTTTCTGGGTAAAATGATGTCCCTATGGTCTTTGGGTAATCA
ATCCTGGTGGGGTTACAAACCACATGGCCTCTATGTAAAGATCAATAGTGCGTTCGTGCC
AGTCATAGGACCCCCTTGTTTAATCTCCCAATTTGTATACCTATATCAAAATTTCAGTAA
ATTATCAATGAGCACACTTGGTGTAATATTTACAATGATACCTATGACATTTTTAGTTAA
TGTAAAAGTACGGGTGCATAAAACGAAAAAGTATAAAAAGCTTATGAAAGTATTTTTGTC
TGAGATTCACCTGTACAACTTTATAGAAGACGACAATAATGTTAAACAGATCGTTATCAG
AATAGAGCGTTACACACGTTGGATGGCTTACTGCGTGGTGATGCTTGTGACAGTCAATTG
GTTGTCATGGGCGGTGATACCAATTGTGAATAACCTGAAATTCAAAGATGACGTACAAAA
CAAAACTATGCAGTTGCAAACTAGCCTATATATGTGGATGCCTTTTGATTATGAACACGA
TTATCAGAATTGGATTATCATCCATTCCTTCAATATTTATTTAATTGCCATTGGATGCGC
ACTTTTATCTATCCCCGATGTGATTAACTATGTATTTTTATTCCATTTAGTAGGACATGT
TACACTTTTGAATCATAGAATAGTGTCACGATTATCATTTGAACTAACTGATAAGGAAGT
GGAAGAGAGATTAAAAGATGTAGTTGAATATCACACTTTTATTAACAAATTATTCAAAGA
TGTGGAATCAGTGTTCGGTGTGAACATCTCTATTAATTACTTAAACAATTTAATTGTGGA
TAGCCTTTTGCTTTTTCAATCGATTAATCAGGAAAAAGGCGATACAACCATGTTTATATA
TGGTGTGGCATTTGTGATTTGCATGGGAGGACTGATTTTAATGTCTTTTATTCTAGAGGA
AATACGTAACCAAAGCGATGTTCTACCGGATTCTTTGTACGGCGTACCATGGGAAAATTG
GAGTATTCCGAATAAAAAATCATTTCTAACTATCCTGACGCGGTTGCAACCTGAATTATC
ATTTGTGGCTGCAGGAGGACTCCGCACTGGAGTGACACCGATGATATCTATAATAAAATC
CACGTTTTCTTACTACGTCATGCTGAAGTCAACCATTTAA

>EhipOR15
ATGTCCGTAGCAGAAGAATTTGATAAAGCATCAGAAATAATAAAAATTTT
CTTTAAATTAATGGGTATTCATTTAAATGAAAATAAAACAATTATTGATCACTTTAAAAG
TTACTGGTTCTACTATTTTAATTTCATTTGGTTAAATATAGATGTTCTGGGAGAAATACT
GTTCGTGATTACTGGAGCCATCAACGGAGAAAGATTTATTGACCTCACTTACATGTTACC

ATGTATCGCCGAGTGCTTATTGGGTGATTTCAAAACTTACCATCTCATTAAATATTCACA
CCACGTCAGAGATTTGACTCACACTTTAAGAAATATGAATTATCACGAAATGTTTACCAA
CCAAGAGGTTGAGAAAAAAATATTTCAAGAGTCATTTCCATTTATGGATTTAGGTGTGAA
TACACTTAAACGGTGTAATATTGTCGCTCTTATAAATTTTGGTATGAATCCTATGTTCGT
AATGGCCAGTAAATATTATACGACTCGTAAATTTGAACTCTACTTACCTTTTCATATTTG
GTATCCTTTTGATGCTTATAATCATTATCTGTATCCCTTTGTGTATGTTCATCAGGTATA
TTCAGCATACGTAGCATTGTTTACCGTCTATGGACCAGATAGTCTTTTTTACACTTACTC
AACTTTTGTTGGTGTTCAATTTCGATTGCTTAAGCACTCTATCGAGCATATTGTACCACC
AACATTTACTTCTACGGAACAAGAATTAGCTGATTTTCATATTAAAATTGAGAAGATTGT
ATTATGGCACCTTGAACTAGTACGATGTGTAAAATTATTGGAAAACATTTTCACAAAATC
TACTTTGTTCAATGCAATCACGAGTTCATTTCTAATATGTCTTACGGGATTCAACATAAC
AGCTATTGACCACTTGCCATTCGTAATGACTTTTGTGGCGTTCCTTTCTGTGACTTTTTT
GCAAATATTTTTCTTTTGTTCTTATGGTGATATGATCATGAGACTGAGTGTCGAAGTCGG
TGATTCCGTTTATAATTGTCAATGGTATTTAGTGTCACCTTCAACAGCGAAACAGTTGGT
GATTATATTAACTAGAGCTCAAATGCCTTGTAAAATGACTGCACTTGGATTTGCCGATAT
CAATCTAAAAGCTTTCACGAGAATATTAAGCACGTCTTGGTCTTATTTCACACTGTTAAA
AACAATGTATAGTTCACCGGGCGCTGAAAATGAATAG

>EhipPR2
ATGAGTAAAACTGAGACCGTAATGGATTTAATATACATAAAGCAGTTGAGAT
GTTGTTTAAATGCAGTGGGGTGTTGGCCCAGAAAGGAGACAGGCCAGAAACCTATAAAAT
ATTTGTCAGTTTACAGCGTCATCTTAGTTCTCTTTGCGATATTTATGATTATTGATGGTT
TTTGGTATATAAAAAATAATATTCAAAATATGGATATTTTTGAAATTGGTCACACCTATA
TGACGACTTTCATGTCGTGCAGTGCCGGATATCGCCTCACGCTTCCTTTTCAAAAGAAAT
ATAAAAGGATGACTGAAACTTTCATAAAGCAATTTCATCTCTTACACTTCAAAGATAAAT
CAGATTATTCAATGAAGATTTACAAAAAAATCGATAAATTGTCACGGTACATAACAATGT
ATTTCAATTTTCTTCCATGGCTAGCCGTTCTGGGCTTTCAATTGCAACCATTGTATTATA
ATTACGTCAACGGCTTATACTCTTCGAACAGACCAGAAAACACAACATTTAAACACACAG
TATATTATGTTTTGCCATTCGATTATGAAACCAACGTATATGGGTATATATGTATATATT
TATTCAATTTGTATGCTTCGTGTGTAAGCGCTGTATGCTTTGCTTATGTCGATCTATATA
TGTTGTTACTTATTTTCAATATATTGGGACATTTGAAAATACTGTTGCATAATTTGCAAC
AATTTCCGAAACCGCAAGAAAGTGATTCGGTTGTGGATACCATAATGTTTTCAAATGAAG
AAATGGAAAACATTTTTGAACTTTTGAAGGAATCCATTAATCATCATAGAATTATAATGG
ATTTTGTGTCGCTAACTTCGGAAGCGTTAAGTGCATTGCTTTGCATATATTATGGGTTCT
ATCAAATCATACTTTTCATGGTACTACTATTGTGTTCACAGTTGGATATAAATGCAATCG
TCAATTACAGTATCGTTGCTTTGTTTTTCTTTCAAGAATTGATTTTAACGTCCATGATTT
TCGAACTTTTGGGGACTACGAGCGACAAAGTCAAAGCGGCCGTATATGAGTTGCCGTGGG
AGTGCATGGACACAAAGAATAGAAAAATAGTTCTGTTCTTCTTGAAAAAAGCGCAGGAGC
CGATCGAATTGAAGGCGCTGGGCATTCTGCCTGTTGGCGTTAACACCATGGCCGCTATAA
TCAAGAATTCAATATCGTATTTCCTTATGCTTCGTGCTACAGTATAA

>EhipOR16
ATGTTACAAAAAATCAAAAACTTTTGCAATAAAAAAGGTTTTGACTAT

TTAAATGGAGAAGTCAACGCGTTTGAGTTTCACAACACGTTTTATTTTTACATGAAAGCT
TTCGGAATAGTAAAGGATAAATCGTTAAAAAAATATGTTACCATTATGTTTATAATACTA
CTGATCTTTGCATCGACTCACATGACTTTGGTTATAATAGCTACCGTGCACGGAGTACAG
ACGTTTAATTTAGCCCTGGTAACCGAAGCTGGAACTTATATCATGCTTATGATCTATCAT
TTCATCATATTGGGATCTACTAAATTCAATTTAGTACATTATTTACATCTATTAGATCAC
CTTAAAGAAGATTTTCGATATATATGCAACGAGGGTTCTAGATATAGGGAAAGATACTTC
AAAATTCAAGTTGAATCGTTTCAAATATGTTTGGCATGTCAATTTGTCACAGCGGTTGCT
GTTGTATGGATGTGCACGTTTGCAATACTCAACAGCTCATGGTATCTGATTACCTATAAG
CCGGGTGTAAGAAGAACAAAATTCCACTTGCATCCGTATTGGATCTTCGATTATGATTTA
GAGGTTGAACCGGCTTACAGTTTAGTACAGACTTTTAGCGTTATAGCGACTTTGTTTTTC
GGATACGTTTATGTCTTCATGCTACAAACCCACATATTATGGGTGAGACAATTAGCAGCA
AAAATAGATTTGGTGGTGTGGAACCTTGAAGACCTGGTGGATGATTTGAAACAACCGACC
AACGATTTGGAGAGAAAGCAGTTTGATAGTGTAATTCAAGACAGAATGAAGAAAATATTA
ATAATTCACCAGTCAATGTACAGCTTATTTATACATTACTGCAAAGTTTTTAGGAAAATG
TTATTGTTGGAGCAGAAAATCTTCAGTCCTATTACTTGTATGACTGCTTACTGTTTTGTC
CAGAAACTAAATGGTGGAGAATTCGACGCAGCATTATTACAAGTGTGCGTTACTGTAATT
TTTGAGGCATTCATTCCATCGTACCTTGTCACATTTCTTGCTACAAAGATCCGATCGGTA
TGCGACGCCTGTTTCGCGGTTAAGTTTTTGGAGACCGGTCCCATAATACGGCCTTATCTA
GTGTTGATGATGCAGCGTTCGGTACGCCCTCTACCCCTAAAAGCGGCTGGTTTCGAAGAA
GTGTCACTTAAGACCTATTCAAGCAATTTGGCATCCGCTTATTCCATGTTCAACATGTTG
CGACAGATTAATTTTTAA

>EhipOR17
ATGTTTAAAAAAATCATAAGGTATTGTAATAA
AGAAAATTTTGATTTTTCCACGGGAAATATCGATGCATTTGACTTTCACGAAGTGTTTTG
TATGTTTATGAAAAGTTTTGGATTTATAAAAAGCGAACTGAAAGGAATATGGATTACTAT
ATTTATTTTAACAATAACAACTTCGGATACACTTCATTTCATTCTAATGAACATCACTTT
GTATTACGCTATACGTATTTTGGATATAGCATTGATAACTGAAGCCAGTTTATTCGTCCT
TCTAATGGCGTATTTGCTTATTATATTAGCTTCGCTTAAATTGAATTTCTGCCAATATAA
TCGACTATTAGAAAGTCTAAAGCATGATTTTCACTACATTTGTAACGAGGGTGCTAAATA
TAGAGAGAGATATTTTAAAAATGAACTTGTAACTTGGAAAATGTGTTTGGGCTGTGTTAT
ATTCACGCTGGCCATGGGTTTAAGCATGTGCATATTTCAAATTATTAGCAGCGTCGTCTA
TTGTGCTACTCACAAAGCAGGAGAGAGAGTCCAGAAACCTCTACTGTATCCCTATTGGCT
CTTTGGATACGATTATAACGTTGAACCTATATATAGCATATTACTATTTTTGAACGTTAC
ACTTATTTTGTTTTATTCTTACACATATGTATTTATGGTGCAAACCCAAATACTTTGGAT
TAGACATTTAGCAGCTAAAATAGATATAGTAGTGTGGAATCTTGAGGACTTGCTCGAAGA
TACTTATCAACCAACTAATGAATTTGAGATGAAACAGTTCTTAGACCTAATTAAGAAGAG
AATGAAACAAATAATAATATTTCATCAGTCCATGTATAGCTTATTAGATGCCTACGCAGC
GGTATATAAGAAAATGTTAATGTTCGAACAAACAGTGACTGCGCCGCTTACTTGTATGAC
TGCTTATTCCTTTGTTCTGAAGTGGGATGATGGCGAATTTAACGCATCGTTTATTATACT
ATGTTTTTCAATAATTGTACAAGTGTTCATTCCTAACTATCTTTGTACTTTTCTTTCTAT
GAAGGTTCAATCAGTATGTGACGCCTGTTTCTCGGTTCCGTTTTGGATGATCGGTCCCAC
AATACGCCCTTACCTGGTGTTAATGATGCAGCGTTCTTTGCGACCTTTGCCGCTTCGAGC
GGTAGGCTTCGAAGAAATATCACTTAAAACATTTTCAAGCAAAATGGCATCTGCTTACTC

GATGTTTAATATGTTGAGACAGATTAATCTTTAA

>EhipOR18
ATGAACCAAGA
TGTCATGTACAAGGTTTATGACATTGACGGCATAGGAAATGACGATCTCAATGAAGATGA
TAAACAAAACAGTCCCAAGAGTTGCAAATCGAATGATGCTAATAGTTCACTATACAGAGG
ATCCAATGATGACAAGAGAAATGTGGTGAAGTCTTCAAAACAGATCCTGTTGAATGCTGG
ACAGAAATCAGAATTCGATTCGGGGATGAACAATTGTCAAATCAGTCAAAATCATTTAAA
GGAAAACAGATGCGGAACTATCAATGCGGACACAACATATTGCACACCGGTTTATGGTCA
AATAGTCTCAACAAGAGAAAATCACAATCAAATCAATGATATACAACAAAAGTTTGCTTC
AAACGAAATGAAACCTTCGACTTCGTTTAGGAAAGACTGGAACACGGTACATGGGAAAAC
CACAGAAGGCTGGGACGAGTTCTCAAAAAGAAATATATCCACAATAAAAAACGGTGATGG
CGATCTTAAAAGACAGAGATGTTTCAAGACTGAAAAGACGAAAGATGTAACTAATGGTAA
GTCTCATTACGACTTTTTCCCAGTTTTCGACAATAAA

>EhipOR19
ATGGGAATTAATTTTCAAAATATTATAAATAAATAT
TCAAAAACTGAAGGCAACAGAACGGAAGTGGATGAAATAATGACCGTAGCTTTGATTTTG
CAAAGAATTTTCGGTCACCAAGTTTTAGACCCCGATTGGACTTGGAAAAGATACTTTTTG
CATCAATTTCTTACTCTTCTACTATTTATTTATGTATTTTTTGGTACTTTAGAAGTCGTC
AGCAGCACATCCGATCCAGAATTAATAGCAGAAGCTGGCTACACGCTAGTACTAATCATA
ATATGTCCAATAAAACTTATAGTTTTCATAAATAACCGGTTTATATTCAAAAAACTTTAT
GTGATAGCAAAAACTACACTTTACGAGGCTATAAAAGTGGATTCGAAGGCGAAAATCGAA
CAAGTCTTGAATAAAGGAAAGAAAGTGACGTATACATTGTTTGGGATGGTCGTAATCCCG
GTTTTTGTATATGAAGTTACAACAATTTGGAATTATTTCAGAGGACGAAAGATTCTTTTA
TCAAGATCGACATCGACTCTCATGCCTATGACCACGCCATGTTATGAAATAGGAATATTT
TTTCATAGCGTTTTTTTGACTGAAGTATCTTCTATTACGATAGTAATGGATATGTGGTTC
GCTTTTTTAATGTTTTTCTTTTGCGTAGCGAGCGATAGTTTGGTGAATATTCTGGAAGTC
AAGTCTAAGGTAAAATATGAAAGCGGGCTAACTTACGCGAAGCGTCTGAATGAGAGTTTG
CGGAAATTTCACAACGCACACGTACAGCAAGTCGAATATTTGAATACCGTGAATGCGATG
TACAAATGGTTAGGAGTGATGCCTCTCTGTCACGCGGCTATGTCTATCTGCATTGTTTTA
CTGGTTATAAGTAACGACATAAATTGGAGGTTCGCTGTTCATATGTTTCCATTGTTTGCT
GAGATTTTTGTCTACAATTGGTTTGGAGAACAGATTAAAATCAAGGCGTTCGATTTAAAA
ACTGCCCTCCTCAATTTCGACTGGATGGGCTTGGAAATTAAAGATAGAACCCGTTACTAT
ATCATAGTAATGTATATGTCAAAAGAATTTGGAATCAAAACTGCCGTTGGGAATTATTTG
TCCATGATCACTATGAGCAATGTACTCAAAAGCAGCTATCAGGCGTTTACGGTTATTCAA
TCTGTCAGCTATTAA

>EhipOR20
ATGGAAATTCCTACTTTCGAAGAATCTTTTAAATTAATAAAAAGGAACTTCTGGCTTTC
TGGAATCCCTTTAGAAAATACAAAAACGACAATCAGATTTTTCCTTTTATATATATCCCT
GCTATTAATGATCATAGAAGAAACAGCGTTCTTAGTATCGAAAATCTCCACAGAAGATTT
ATTGAAACTCACTCAATTGGCCCCTTGCACCTGCATCGGTGTTCTGTCGTTTCTAAAATT
CTCAGCGATATGTTTTAAAAGGCAGAAAATTCGTCAGTTAGCAGATATGCTCGAACAACT

TCACGCTGACATATCAAGCGACGTTAATAAGAAAACTCTAGTTAAACGGAACATTATATT
TGTAAATAAATTGATAAAATATTACACAATACTAAATCTGATACTTATTAATGTGTATAA
TTTTTCAACACTAGTGTTCATTGGCATTCATTATGCCAACACGAAAGAGATGTTATACAG
ACTGCCATATGCACTGATCGTGCCGTTTTCGACAGATATGTGGCCAACTTGGCTGCTTAT
TTACATTCACTCCATTACAAATGGATTCATCTGTGTCCTATACTTCACAACGATTGATTC
ACTATACTGCATTATGACTTCTTACATTTTTTGCAATTTTATTATCATAAATAATGAACT
TCGATATTTGGAAACCATAAACCCGAAAACTTTGACGAATATCGTTAAAAAACATCAATA
TACTATCAAACTCTCAGAAGATTTAGAAGATATCTTCACTGCTTGTAACTTATTTAATGT
TTTAATAGGATCACTGGAAATTTGTGCGCTAGGTTTTAATTTAACGACTGGAGACTGGGC
CCAAATACCGGGATGTGTACTGTTTCTTTTATCAGTGCTTCTACAAATATTGATGATGAG
CTTTTTCGGGGAAAACATTCTCAGAGAGAGCACGAATGTCGGCGAATCGGCATTTTTTTC
CAAATGGTATGAAGCGGACGAAAGATCCAAGAAAATCATTTTATACATAATGACGAGATC
GCATGCACCTCAAAAACTAACTGCTTATAAATTCTCCGTTATTGGGTACGGAAGTTTCAT
CAAGATAATAAGCACTTCATGGTCTTATTTCACTATTCTCAAAACTGTTTATACGCCTCC
AGCTGAAGTTCAGATTGAATAA

>EhipOR21
ATG
GAAGAGAAATTAGCTTATTACAGTTTAGTGCCTCATTTCAAACAATTACGCCAAACTGGT
TTTTATCCGTTAGATCCTGAAGCTCCAAAATTACAACAATTTCTGCATAAATTATATAAT
CGCTTCAGTGTATCTCTTCTACTTTTGTATACTATGCAACAAATAATAAACCTTTACCAG
TGGCGTCGTAATATAAATAAAATAATGGATTCAATGTTTTTATTCTTGACCTATTCCGAT
TGTCTTATCAAAGAGGCTGCATTCTGGAATAAAGCTGATGAAATTGAAGGGTTGATGAAT
ATAATGAAAGGTCCCGTTTATAACCAGAAAGAACACAAACAAATTTTAATACGAACCGTC
CGACAAGCGAAACTTGTACTTCGCATCTTCAACATCATGTGTCTAATAACTTGCCTTCTG
TGGGCCATATTCCCAGTGATATTTCACTTAAGAGGAAAGGCTGTAGAATTTGGAATATGG
CTGCCTTTTGATTCCAATGCGAATCCGCAATTTTACTTCGTTGTTATCTATGTATCTTTC
CAAACATCATGGCTTGCTTATAACAATTCTACAATGGATGTTACCATGGCGTTCTTTCTG
GCTCAATGTACAACTCAATTTTTTATTCTCAGACAAAATCTCGAGAACATTGTTAAGAAA
AGCATTGATGACTCTACCACCCTGCGGATCTCTTTTGCAAAGGCTTTTGAGAAACGTTTT
ATCGGTGTTATGCAACATTACAATGAGATTGTTCAATCGAGTCAGAAAATTGAAGAAATA
TTTGGAAACGCCGTGTTTGTTCAGTTTATTTTTACCGGATGGATTATTTGCACCACGGCA
TACAGAATGATCAATATGAGTCCAGGTTCAATAGAGTTTCTGTCTATGATATTGTATATA
CTTTGTGTAATGACGCAACCATATTTATATTGCTTCTATGGTAATGAACTAATGTATGAG
AGTGACAAACTTATGAACTCTGCATACGCAATGGACTGGCTTATGATTGCGCCTAAACAA
CGACGATACTTGATTATTTTTATGGAGAGAATTAAACGCCCAATTCAACCTAAGGCTGGA
TTTGTCATACCACTTTCAGCTAATACTTTTGTTACGATCCTGCGTACATCGTATACATTC
TACGCATTTTTAAAAAATTCATATTAA

>EhipOR22
ATGAAAATTTTAACCGAACATGTA
AAGAAAAAAATTGTCTATACTATTACCTATACTTCCTTATGGAGTTTTGGAGTCTTGGGAA
AACCTCGATCCGAGACTGTATCACGCCGTACACATTTACTGGCTCAAGTTCTACGGGATG
TGGTACAACAATTTTTCACCAAAAAGCACCCTCTTCTGGCTTCAGTTGATATATACGATG

ATCGTGTTATGGCTAGTGTGTTTCTTGCCCGGCATCGGAGAAGTCGTTTATTTACTGAGG
CGGAGAGATAATATTGGTGACATCGCGGAAGGGTTATATTTATTTCTGAGTGAAATGTAC
ACGTATTTCAAAATAGGCGTATTCTGGTTGAATAAAGATAAAATTGTAAGTCTATTACAA
TATTTGTATTGCGAAGAATTCAAACCAAAAGAGACGGAACACAAAGAAATTTTACGGAAG
AGCATTAAAAGCGCGCGCTTCGTGATGACATATTATTCGACTATATGCGTCGGAGCTGTA
TCTGTTGGCTGCATTATGCCGATAAGTGAAAATTTTCAAGTGTTACCAACAAATGTGGAA
TATCCATTTTTCGATGTGTACAAATCTCCAGTCTACGAAACTATGTATATTCATCATATT
TACTACAAGCCAGCCACTTGTATAATCGATGGTGTAATGGATACCATATTGGCTGCCTTT
GTCGCCTCTGCAATCGGACAATTAGAAATATTAGCGTACAATCTACGAAATTTCGACGTG
ATTGCAGAAAGAAGACGCATTAGAGATAATAATCGCATGAATACGAAAGTTTATTCTAAA
GAAAATTATATGAAAACAGTGCTCAAGGATTGTATTAAACATCACAATAGCATTATTCGA
TACGTGTCGATGATCGAAAGCGCTTTCAGTTTGGCATCAGCATTGCAATTTATGCTGAGC
GTTATGGTCCTTTGTTTGGTTGGAATACAGTTTCTGTCGATAGAGAATCCATCCAGTCAT
CCCATGCAGATTGTTTGGATGGCAATTTATTTAACTTGTATGCTGATTGAAGTATTCATA
CTGTGTTGGTTTGGCGATGTATTGATTTGGAAGAGCATGGAACTCCGACAAGCGGCATTT
GAAGGGCCCTGGCTAAATGTCAACCCAAAGACAGCGATGTTCATCATTATATTCTTAGAG
CGTTGCAAACGTCCGCTCAGAGTCACCGCTGGCAAGATATTTACGCTATCCCTCGACACC
TACACTGATCTTATCAATTGGGCTTACAAAGCGTTTGCAGTAATGAGAAATATGAAGAAA
TAA
>EhipOR23
ATGAAGATTTTAGTGGACAATGCTAATTTATCCATTAGTTTGTCACTCACAGCTTTGA
AATTGGTCGGATTTTGGGCACCCGACGGCTTGAACGGCATCAACAAGTTCTTATATAACT
GTTATGCACTATTTTCCTTTATGTTTTTGTTGGGTACATATTTAATAATACAAGTAGTGG
ATTTGTATTTCGTATGGGGCAACCTTCCCCTGATGACGGGTACCGCATTCCTCCTCTTCA
CGAACCTAGCGCAAGCCGCGAAGATCATCAACATATTGTCGAGGAAACGACTCATACAAA
AAATCATAACGGAAGCGGATTTGGTTCTCAAAGGACAGCAGACTGAGGAGGGGAGAATTA
TTGTGAAAAACTGCAACCGCGAGACGAGTCTGCAGCAACTGCTGTACTTCTGCTTGACCA
CCATCACCGTGTCCGGCTGGGCTGGCAGCGCTGAGAAGAACCAGTTGCCTTTGCGAGCTT
GGTATCCCTACGACACATCAAAGTCTCCAGCGTACGAGCTAACGTACATGCATCAGGTCG
GAGCTTTATTCATTGCTGCGTATCTGAATGTGGGCAAGGATACCCTAGTGACGGCACTTA
TAGCGCAGTGCCGTTGCCGGCTGCGGCTCCTGGGTCTGGCGCTGAGGACGTTGTGCCGAG
ATGTAGAAGTCACTCACAACCATCTGCTAACAGCTAACGAAGAGCAAGTGGTGAGCTCCA
GGCTGCGTAACTGCGTGTGTCAACACCAGGCGGCGCTGGCAGCGGCGTCGGAGCTGCAGA
CTTGCTTCTCAGCACCCACCTTCGCTCAGTTCGCCGTTTCTCTCGTTATCATATGCGTTA
CTGCTTTTCAGCTGGCTTCTGTGTCGCACACTGGAAACCTCGTCCGCTTGTTCTCCATGG
GCACGTACCTTCTGAACATGTCGTTCCAAGTGTTTCTCTATTGTTATCAAGGCAACCAGC
TTTCGGAGGAGAGCACCGAGATAGCGGGCGCGGCGTACTTTGCTCCATGGTACGCGTTCT
CAGCGCGACTCCGGCGCGCCATGCTGGTGCTGATGACGCGCTCACGCCGCGTGGCACGGC
TCACTGCCGGCGGGTTCACCACGCTCTCCCTTACATCCTTCATGGCTATAATTAAAGCGT
CATACTCCTTCTTCACTGTTCTGCAGCAAGTTGAGGAAAAAGCATAG

>EhipOR24
ATGAAAGTCTTAAAATGTTTAAAGAA

TACTTATTTGGATATAAAGAATCATCTCCAAGATGATAGTTTTGATAGCCTATTATGGCT
TGTAAATATAATGCCTAGTTTAGCTGGTTTTTCTCTACGCCAAGATAAAATAGCTGCACC
ATTTTGGATATTGCACTTATCTCTGCTGTTTTATGTTTACGGTGTTGGTAGTGTAGTGTA
CCAAGTGAAATACGCCCAAAGTCCCACTGAGTTCATTAAGAGTTACGTCAATATATCAAT
ATTTATCATTGTAGTGAACGGTAGCTATTGGTTTTTGACGAAGAGGTCATTAGTAAAAAC
CGTGTTAAAGAAAGTAAACAAAAGCGACGAATTAGCGAGACGTTCTCTTTTATTAAGAAC
TAAACACAAAAAATCACTTTTTGCTATAAAGAAAATCGTCATGATGTTTTACGGTGTTAA
TCTTTTCCAAGAATTCATTATTTACATGCCTCATCGTGTTGATGTGTTTAACGATAATTA
TTCTATGACACCGTGTGTTGGATTGGAACCTTTAACGGTGAGTCCTAATCAAGAAATATG
TCGCTTAATACTATGTGTCCAAGAACTAACAATAATGACTGTTGTATTGGACTATCAAGC
TCTTTTGTTTCTTCTCATAGCTCACACGACGTTGATGTACCAAATGTTGGCGGAAGAAAT
AATGACTCTTGCTAATAGCGATGAGAACATTCAAGACGTTAAAGAAACCCTACCTAAGCT
AATAAATCGTCATATAATGATATTGGATACAATTGATAAACTAAAGACTTTGTATAGCGT
TCCAACAGGTGTAGACTTTGGCTCAAATGCCCTTTGCATGAGTTTATTCTGTTTTTTAAG
TTTACAAGAATACGTCACATTCATGCCAATTATACTATATGTTTTTGCTGTGTTCTTTCT
CTATTGCTATCTATGCCAGTGTCTTATAAATGCTTCGGTAGATTTCGAAAGGGCAGTATA
TAGTTGTGGCTGGGAAAATCTGGAATGGAGAGATAAGAAGACAATATATGTGATGTTATT
GCAAGCCCAAAAGCCGATTGAATTGTTAGCGGCAAATATAATACCAATTAATATTGCAAC
ATTTGCCAGCACTATCCAGGCGATATATAAATTTGTAACTGTTGTGAAATTTTAA

>EhipOR25
ATGGGCCTCGTAGAC
TCTCTGTGGAGGAAATTAACTCAAACTAAAGCTTTAGAGGAATCGAGTGGGAAACTGGAA
ACTTTGTTCTTTGAATCCGTCTATCGAGTAACCTACTTAGCAGGTCTGTCCTCCAGCGAC
ACACATCCGGTCTACCGCGCTTACAGTATCGTCGTAAAGTTGTCGATAATTCTGTTCGTG
AGCAGCGAACTGTGGTACTTGGTCAGCGAGACATCCAGCATGGACAAGATTATAGATAAC
ATCAACGTTACACTCATACATTTCATAGCCATATATAGATACAAGAAACTGATGGACCAC
AAAGATAAGTACAAGGAGCTAGCAAAATCTATGGAGTCGCCTCACTTTGATATCTCTACA
CCTAAGAGGAAAAAAATTGGTACAGTTTTGGGTGATAAGGAACGAAAGATATTTGAAGTTA
CTGCTTGGATTGGGAACTTGCACGCTTGCTGCTTGGTATGTCTATCCTCTTGTGGACGAT
TTGGAATACAACCTCTCGGTAGCTGTGCGTCTACCGATCGAGTACCGCACCCCCTCCCGC
TACCCCCTCGCCTACATTGTGGTCCTGATAACATTCCATTACATATCCTATTTCGTCATC
GTGAACGATCTCGTCATGCAGGCACATCTGATGCATTTGCTCTGTCAGTTTGCAGTACTT
GCTGATTGCTTTGAAAATATTATAAACGACTGTGACGTCGAAACCCAAGGTATCAGCCAG
AATAAATTGTTTTTGAGCGAAAAATTTAAGGACAAATACCTTTGCAGATTAAACGACCTG
GTGGATCAGCATAAGTTTATATTACATCATGCAACGACATTAAAGAAGATATTAAGCACA
CCAATGTTGGGTCAGCTGGCCGCTAGCAGTATGCTTATTTGTTTTGCTGGTTACCAAGTT
GCAACGACGGTGACGATAAATCTTACAAAGTTTGTCATGAGCCTTCTATATTTGGGATAT
AGCATGTTTGAACTATTTATATTCTGTCGTTGGTGCGATGAGATCAAAATACAGAGTGAA
AACATACGACTGGCGGTATACTGCTCGGGTTGGGAGCGCGGAATAGCAGCGGTGCCCGGC
ATAAAAACGAGACTCATGCTGATAGTCGCTCGCGCCAACAAACCCATGATCTTAACTGCT
GGAGGATTATACGACCTGTCACTCAACTCTTATACCACTGTGGTGAAGACATCGTACAGC
GCGTTGACCGTTTTACTTCGGCTCCGTCAGGAATAG

>EhipOrco
ATGATGGCCAAAGTGAAA
GCCCAGGGCCTCGTGTCCGACTTGATGCCCAACATCAAGTTGATGCAGATGGCCGGGCAC
TTTTTATTTAATTACCATTCAGATAACAGTGGCATGTCAACTTTGCTTCGCAAGATATAT
GCTAGCGTCCATGCTGTCTTCATCGTGACCCAATATTTTGCGATGGTAGCCAACATGGCC
ATGTATTCTGACGAAGTTAACGAGCTAACAGCCAATACCATAACAGTACTATTTTTTGCT
CATTCCATTATAAAGTTGATATTCTTCGCACTCAATTCCAAAAGCTTTTACAGGACTTTG
GCAATATGGAACCAGTCAAACAGCCATCCACTTTTCACTGAATCTGATGCAAGGTATCAC
CAGCTCGCTCTCACCAAGATGAGGAGACTTTTGTACTTTATCTGTGGCGTAACAATACTC
TCGGTTTTCAGTTGGGTGACGATAACATTCTTTGGAGAATCAGTCTATATGTTGGTCAAT
AAAGAGACGAATGAAACTCTCACGGAACCTGCTCCGAGGTTACCTGTGAAAGCTTGGTAC
CCTTTCAACGCAATGAGTGGCACGATGTACATCGTAGCATTTGTTTTACAGGTGTATTGG
TTACTCATCGCGATGGCAATAGCAAACTTGATGGATGTGATGTTCTGTTCCTGGTTGATC
TTTGCTTGTGAACAGCTTCAGCACCTGAAGGCCATTATGAAACCTCTGATGGAATTGAGT
GCCTCTCTTGATACATATAGACCCAACACTGCTGAGTTGTTCAAAGTTTCATCTACAGAA
AAATCGGAGAAAGTGCCCGATCCGGTCGATCTTGATATCCGGGGTATATATTCTACTCAG
CAAGACTTTGGCATGACATTGCGTGGTGCTGGAGGCAGACTACAGACATTTGGACAGAAT
TTAAACAATCCAAATGGATTAACTCAAAAGCAAGAAATGTTAGCCAGATCTGCAATCAAA
TATTGGGTGGAACGTCACAAGCATATCGTCAGACTAGTCGCTTCTATCGGAGATACTTAT
GGTACAGATCTTTTGTTTCACATGTTGGTGTCAACTATCACTCTCACTTTACTGGCTTAT
CAAGCAACGAAGATTAACGGATTAAATGTATACGCATTCAGTACGCTTGGCTATTTGGGT
TACACGCTCGGTCAAGTTTTCCACTTTTGCATTTTCGGAAATCGACTCATTGAAGAGAGT
TCTTCTGTGATGGAAGCAGCATACTCGTGTCAGTGGTACGACGGTTCTGAAGAAGCGAAG
ACGTTCGTCCAGATCGTGTGTCAGCAATGTCAAAAGGCGATGAGTATCTCAGGAGCCAAA
TTTTTCACCGTTTCTTTGGATTTGTTCGCTTCGGTACTGGGAGCGGTAGTTACATATTTC
ATGGTGTTAGTGCAACTCAAATAG

>EhipOR26
ATGGATGTAAC
ATTGAGCGTGTACCACAAAACTCTTTCAGTGATTGGCATTTCGATATTTGCTAGAGAAAA
ATGGGATTCAATACCTTGGCTAATAATACAAACATTTATGTTTATATACGCACTTTTGAT
CCTTATTTTCTGCACAGTATTTATGATTCAAAACTATTCAGATTTATTGATATGTATTCA
AGCCGCCTGCGTTTGGAATACTGGAATTTTAATGTTCATATGCATCACAATTTTTTTTGT
TTATCGAAAGAAATTTAGAACGTTTTTAACAGAGATTGTATTCCAAGATCCAATGTTGAA
TATGCCTTTTATTGAACAAATTTTGAAGTTATTTCTCGGTGGGAAATTGAACGAATTAAA
ACAATTAGTTATTTATTCTCAAGACAAATTGTTTAAGTTATCGGATATATTGATAAGAAC
TTATTTAGGAAGCTTTACTTTATGCTACGTTTTGTATACATGCAAACCCATTTATCGTAC
ATTTATTGGAAGAGAAAATAAGGATGTCCGTATAATGGCATTCGAAATGTGGTTTCCATG
GGGTTTGAAAAATGACTATGTATACGTGGCTTCGTTTTTATTTAATCTATATGGCGGTTA
CATATGTTGCTTGTCATGTTGCGGTCTACAATCAACTATATTTCTTTTGTTTGGTCAGAT
GATACGCCAACTCAAAGTACTAATCTTCATACTTCAGAATCTAGACGAGTTAGTATTAGA
ATTGACGGGAAAAAGAGATGAAAGATGGCAAAAGCACTGTACTCTTATACTGTCGCAGTG
CGTCGACCATTATATAAAATTGAAAAGGTTTAACAATCGTCTAAATGTCATATGTCAACC
GTTTTATTTGGCTCTTATATTGGATGCGATTATGTTAGTTTGTGTTTGTTTTGTGAAAAT

CGCTATATCAGATAGATTTTCCGAAGATACATTAAAATATTATATTCACGCATTTTGTAT
TGGTTTCATAGTAATGCTGTTTTGTTTTCTTGGCCAGCAGTTGAAAAATGAATGTGACAT
TTTAGAAAATGTTGTTCTTGAAAAATGGTATATTTTCGATAAAAAACACAAGGTTTGTGT
TCAAATTTTCAAGATGGCTGTCAGTCATGGCATGCCCGTTTATATTTTTGGATCTGTTAC
GTTGACTTTGCCCACTTTTACTTGGTTCATACGAAGCGTCATGTCATTCTTCATGTTGGT
GATGACTGTTTTAGAAGACGACAATAATAATTGA

>EhipOR27
ATGGAAGATAAAGCAATATTTTCCACCTT
TGAAACTTTCAGACCCCTCTTCGATGGGTTGGCAAGAGTGGCTTACTATAAAATTGTAAT
GAAACCTGTGTCCCGAATGAAATATTGGGCACATTTTTCATATCGTTTTATTGTATGGAC
TTTAGTGGTCATGTATAATTTGCAACATGTCATACGTGTTATAAAGAGCCGTCACAGCAC
GGAGGAGGTGGTGAACACTCTCTTCGTTTTGCTGACAACGCTCAACACCCTGGGCAAGCA
GATCGCGTTCAACTCGCGCAGCCGTCGCATCGATCGCCTCATCACTGTTATAAACGGACC
TTACTTTGCACCGACCAACTCCTACCATGTGCGCGTATTAAAGGAGAATGCTGTTACGAT
GTCTCGATTACTGTTACTTTATCATATAGCCATCTTCATCTGTGGTACGCTTTGGACGAT
ATTTCCATTGGTGAACAGAGCTACAAGCGAAAATGTCGAGTTCACCGGTTACTTTCCTTT
TGACACCACGCCTTCACCAGTGTTCGAACTAGCGCTCGCTTACATGTCCTTCCTAATAAC
ATTTCAAGCATATGGTAATGTTACCATGGATTGTACAATTGTAGCATTCTACGCACAAGG
CAAAATACAGCTGCATATGTTAAGACACGATTTGGAACATCTCGTCGACGTCAAGGATGG
ATTTAAAGAAATAGACTCAAAGTTACATGAAGCAGATAATCAATATTTCACCTATGTAGA
TATAGAAAATGTAAGATTTAAACAGAAGATCCAGGAGAGATTGGTGCGTTGCGTGAAACA
TTATCAGCAAATTGTGTGGTTTATTAATGAAGTTGAATCCATATTTGGCGAAGCGTTGGT
TATCCAATTTGCTGTTATGGCTTGGGTGATTTGTATGACTATGTATAAGATTGTTGGTCT
AAGTTTAATGTCAGCAGAGTTTATATCGATGGCCATGTACCTCGGATGTATGCTAGCACA
ACTCTTCATTTATTGTTATTACGGAACGCAGTTGAAATTCGAGAGCGAATTTGTGAATCA
GTCAATATTTTTCGGCAACTGGCTGGCATTGTCGCCCGAGTTCCGTCGCCAGCTCCTGCT
GATGATGATGCGGTATTCACGTGCGATCACTCCGCGCATCGCGTACGTTATACCGATGTC
TCTGGAAACTTACATACAGGTTTTGCGGTCTTCTTATACACTGTTTACATTTTTAGATCG
CAAATAA

>EhipOR28
ATGCCTTTTTATCAAATA
ATTAAGAATTTTATACTAAAAGCATATTTCGATTTCGACAATTCATACATTAGCTTATAC
AATTTTCATCCTCAGCTTCGAATTTTTATTGCGATCAATGGAATTTTCTTTAATAATCGT
GATTCGAAAATAAGATACATATGGCCGATTTTTGGATTACTCTTGACATTTGTAGGGTTT
ACGTTCGAATTAATATTTATTTACCACGGCTTAACTATACAAGATTACTCTTTCGCCACG
GAAAGTTTTTGTTACACCATAATACTTGGGGTCATTCCTATTGTCTACACAACTGTGCTA
CTTAACAAAGAGAAAATTATACAGCTTATAGAAGACATGAATGGAGATTTTATATATATT
TGTCGATTGGGATCAGAATACAAAAAACGTTTTTTAAAAGGTCAACTCTACATATGGATA
TTATGTTTCATGTGGCTAATGTTTCTTAGTTTCGTAGCCGTTGTATTTATAATGACAATG
GTCCTCACGCTCATGTACCAAAGCCTTTTCGCTACCCAGGACGAGTACACGATCAGACCG
TTAATATTTCCAATGTGGCTACCAGAAGACGATCCTTATAGAACACCTAATTATGAAATA
TTTTTATTTTTGCAAATAATATTAATTTTCATCTGCAATTTGACTTTTGGTGTGTACGTC

TACATATTGTTTCACATTCTGTTGCACTATTATTATTTGATGGACATGATAATGCTGGCG
TTTGGAGTATTATTCGATGGCCTCGATGAATCCGTGATGAACCTGCCTCGTTCTGATGTA
AAAAGAATACAAATTCAACGAGTTCTCAACAACAGAATGAAACAAATCGTACGATGGCAC
ACCTCTGTTTTCAAGTCTGTGGACACAGTATCATCTGTTTATGGATCAGCTTTGGTTTAT
CAAGTAATGTTCAGTTCTCTCGCTATATGCTTGATGGCGTATCAAGTTGCTGTCCAATTG
GACGAAGGGAAAGTTAATTTTTTATTTATAATTCTGAGCATTGCAGCCTGTCTTCAACTT
TGGATACCATGTTACTTGGGTACCATAATTAGAAACAAGGCATTCGCTGTAGGCGACGCA
TGCTGGAATAGCGGATGGCACGAAACGTCATTGGGTCGTTTGCTGCGCCAAGACATTATC
ATTTTCATACTGCGCGCGCAGCACCCAGTCACCATCAAGTTCACCGGCCTGCCACCAATA
CAACTCGAAACATTCTCTTCGATAATGAGCACATCGTACTCTTACTTCAATATGTTGCGT
CAATACAAATAA

>EhipOR29
ATGGAGTTAAATTTTGATGAGATATTTAAAATATCGGTG
TGCTGTTTAAAATTAAATCGCTCTTACCCAACAATAAAAAGGGACACGAAATGGTTCTTA
CAATTTATCCTCATGTATAGTATAATATTTTTCGTATTTTCCTATTTAGCGTACTGTATT
ATATTTTATGATTTGAAAAACAATGATTTCACTCAAGCTTGCACAAATGGAATCCTGTCT
GTCGTTTTCACTGTAGTCACTTTTAAATATTTTATCATGTTGTGGTATCAGAAAATATTG
ATGCGGTTAATCGAAAAGATGAAGCGAGATTTTGAATTTGCTAAAAATCTATCGTATGAG
GAGCAATGTTTAGTATTGGAGTACGCGAAAAGAGGACGATGGGTGTCAAAATTGTGGCTC
CTTACTGCTGGAAGTGTTGGTGTCGTATTTCCAGCAAAAGCTTTCATCCTCATGGGATAT
TACGTTATCATAGGTGAATTTAGACTTATACAAATATACAATTTGACATATCCACCTTTT
ATAGAAAGCATTAAAGATAATTACATAGGTTTCACTATTATCTTTTCAATTTTGATGTTC
TACGACTTTTTTTCTGTCATAATGTATATCGGTTTTGCACCTATGGGACCCATTTTCATG
CTTCACACTTGTGGACAATTAGAAATTCTAAAGAGACGTATCCAAAATCTATTTGCTGAT
AATGGAGATGACCTTAGTGAAACACGGACACAACTTAAGATTATTGTTAAACATTTACAA
GAAATATATCATTTTGTAGATGACATCAAATACAGTTTTGAAGTTTTATATGAAATAATT
CTAAAGACAACTGCATTTGTAATACCAATAACGTTTTATGAGATTATAGACTCAATCCAA
CAAGGTCGATTTAGTTTAGAATACTGTACGTTTATTTTTGGTGGCATAACTTTATGTTAT
GTGCCTTGTTATTACGGCGATCTATTGATGGAAAAGGGCGAGAGTCTGCGACAGGCGGTA
TACATGAGTGGCTGGGAACGTTACCGGGACCGCAGCACGCGCTCCACTCTCGTGCTGATG
TTGTCACGAGCTGCTAAACCTCTAGCAATACGAACAGTGTTCCGTATTGTTTGTTTAGAT
GCTTTCACTGAGCTCTGTCATCAATCTTACGCAATATTTAACGTTTTAAACGCAGTATGG
GATTGA

>EhipOR30
ATGTTAA
AAAGGCTTCTTCACAAACTAGAAGACCCAAAAAGACCACTATTGGGACCCAATTTGAAAG
CGTTAAAATTTTGGGGTTTACTATTGCCAGAGAATTTCATAATGAGACAAATATACATTT
TATTACATTTATCTGTGATTCTGTTTACTGCTTCAGAATGCGTCGACGTATGGTTCGTAA
AATCCAACATTACTTTGCTTTTGAACAATTTGAAGATCACAATGCTTGCAACTGTCAGTG
TCTGCAAAATCAGCACTTTCCTCTTCTGGCAGAATGATTGGAAGAAGATCATTGGATATG
TTATCGAAGCAGACATTGTGCAAAGGAAAACAAATGATATCATCAAACAAACCATTATTA

AAAAATTCACAAAGTATTGCAGGAAAATCACTTATATGTATTGGTGTCTTATGTACACGA
CCGTTATAATAGTTATGGTACAACCTATAATGAAGTATTTCTCATCACCAGTCTATAGAG
AAAATGTTAGGAATGGCAACGAAACTTATTTGCAAGTCGTAAGCTCATGGGTACCATTCG
ATAAGAATACAATAACTGGATATTTGATAGCTTCAGTGATTCAAAGTTATGGAGCTATAT
ATGGTGGTGGATGGATCACGTCATATGATACCAATGCTATGGTCATCATGGTATTCTTTA
GGGGAGAATTGGAGCTGCTAAGAAGAGATTGCGCTAATATGTTCGGAACAGAATCTTCTC
CAGTAACTGAGCAGGTTTCAAAGGAACGGTTAAAAGATTGCCATAGAAGATATGTCGCAT
TAGTTAAGCACGCTCGCTTGTTTGATTCCTGCCTGTCGCCTATCATGCTTCTCTACATGT
TTGTATGCTCCGTAATGCTTTGTGTCACCGCATATCAAATTACAATCGAAACGAATCCTA
TGCAACGCTTCCTTTCAGCGGAATATCTCGTATTCGGTGTTGCACAATTATTTATATATT
GTTGGCATAGCAATGATGTTTTATATGCTAGTCAAGACCTCATGTTGGGACCATATGAAA
GTACATGGTGGATGCATAGGATTTCGTATCGCAAAAATATATTCATCCTGATAGCTCAGT
TTAGAAAAAGGATCGTTTTTACCGCGGGACCCTTTACGGAACTTACCGTGCCGACATTTA
TTAATATACTGAAGGGAGCGTACAGTTATTATACTTTGTTAAGTCAGTCACAATCATAA

>EhipOR31
ATGGTAAAAACTT
TAACTGAACGATTGGAAGATCCGGAACGACCATTCCTAGGTCCTCATTATTGGTTAACTA
AAAAAATTGGACTGTTCCTACCGAAAAGTAAACTAGGAATTATCACTAGCTATGTGATTC
ATGAAATAGTGACATATTTTGTCGTAACGCAATATATTGAATTATATAAACTAATCGTGT
TAAAGGCCGATGTAGATCTTTTACTCTTCAATGTAAGATCTTCGATGCTGAGTGTTGTTT
GCATTGTAAAGTCGAATACTTTTCTTTATTGGCAGAGTAAATGGCACGACCTCTTCGAAT
ATGTCACTGAGACGGATAAATTCGAAAGAGAAACTCAAGATGAAGTAAGAGCGAGTATAA
TTAACAATTATACCAAATACTGCCGACGTATTACAAATGTTTACTGGTCTTTTGTGATCT
TTACCAATTTTACTGTGATTTTTACGCCATTGATGAAATATATGACACTCTCAGATGAAC
ATCTTAAAGCTATTGAGAATGGTACTGAGATGTTTCCTCACGTATTTAGTGCCTGGACGC
CATTTATTGATAAAGAACACTCGCCTGGCTGTTGGATCACAATTCTATATCATGCGATAA
TATGTAGCTTAGGCACTTTAATGGTAATCAGTTATGACATGAATGTGGTTGTCATCATGG
TGTTTTTTGGGGGAAAACTCTTACTGTTTCGTGAGAGATGTAAGCAACTGTTTGATAGCG
ACGAAGCTGGTCTTAGTGATGAAGAAGTTCGGAATAGGATACGCGATCTGCATTTAACGT
ACGTGCGCCTTGTTAAGTATTTCAACTTGTTCAATTCTTTGTTGTCGCCCGTAATGTTTT
TGTATGTGGTGATGTGTTCACTTGTGCTCTGTGCCAGCATATACCAGTTAACTTCTAGCA
AGGACACGATGATGACCAAATTCATAATGGCACAGTATTTTATATTCGCATCTTCACAAT
TATTTCTGTTTTGTTGGCACAGTAACGACGTCCTGGCTATAAGTGAAATTATTATGTACG
GACCGTATGAGAGCGAGTGGTGGGCAGCGAGTGTTCGTCAAAGAAAGTGTGTGTTGCTTT
TAATAGGGCAGCTAAGAAAAGACTTTATTTTCACCGCTGGGCCGTTTACAGACCTGACTT
TGTCTACGTTCATAGCGATATTGAAAGGAGCCTACAGTTACTATACTCTACTGAGAGATT
AA

>EhipOR32
CTGGACCACTACACCACTAGTCTACAAAAAGCCAGAAATTGTAGGTTCATT

TCACTATTTGAATTTCCAGTTAAAAATCTAAAATTTTCACAACGAAAACTAATAAAGGCA
AAATTAAACATGGAAAAGGAAAAAATTCAAAATTACCACGATTATGCAGACATAATACCA
TTTAAACTCTCTGCTTTTCTGCCATGGTATATCAAACCCAGGAATGAATATGAAATGATA
TTCAATAATGTTTATTTAGGTTTCGTATTATTCATTTTAATCAATTTGTTGGTCACACTT
TTGGTCAATTTGTACGTGGATTGGATTGATTTCATGTCATGTTTGAATCAAATAGCTGAT
GGGCTACCGTTGGTCGTGTCGATTGTTGTGGTCGTGTACTTCGCTTATTATGAAAAAGAA
ATGGCGCAACTTCAGAGTTTCATGAGAGATAATTTTAAATATCATTCGGCTAGAGGAATC
ACTAATACAACGATGCTGAATAGTTATAAAGTGGCGAGGAACTTTGCCCGCTTTTACACT
GCCTGCTGTCTCTTCAGCGTAACGATGTACACTTTCATACCCATGATTGTGCACTTATGG
ACTAAGTTGCCACCTCAACAGTGGGTGTATGTGGAAGTCACGAGGTTTCCTTACATTCAG
ATCATATTCTTGAGACAATGTCTAGTGCAAGCATTAGTGGGACTGATCATTGGACAATTA
GGTGTATTCTTCGCCACAAACTCCATTCTGCTCTGCGGCCAGCTAGACTTGGTCTGTTGC
AGCGTGCGCAACGCGCGCTACACCGCGCTGCTGCAGCATGGCGTACTGCATGCCGCGCTC
GTCGCGGCGTACGCAGACATACGCGACGACGAACGACACAATTATACTTATAATATAGCA
GAGATGAAGGATTCCGTATATCACTATGATAAGAAAATGGTCAATTTCATCGACACGAAA
ACGGAGTTCGATATATACAGCCGAGACTTCGACCAACAGACTATCGAGGCAATCCGTGAG
TGCGCCCGCTTGTGTCAAGCGGTTGCCAAATACAAAGACATGTTTGAGCATTTCATCTCA
CCCGTTCTCGCCTTGAGAGTGATCCATGTTACGCTTTATCTTTGTATGCTCATGTATTCC
GCTAGTGTGAAATTTGATATGGTCACCGTTGAATACGTCGCTGCGGTCGCCTTCGATATC
TTCATTTACTGTTACTACGGCAATCAAATTATCATTCAGGCGGATCGAGTATCGACGGCA
GTGTATCAAAGCGCATGGCACACCATGGGTCCCTCGCCGCGCAAGTATTTACTCCACATC
ATGCTGTCATATAAGAGACCAGCTAATCTACGCGCCGGAAGATTCCTTACCATGCACCTC
GAGACTTTCGTATCTATCATGAGAACTTCATTTTCATATTATATGCTGTTGGTCAATGTA
AATGATAAATAA

>EhipOR33
ATGAAACGAACGTCGCTAGCGGGCAGC
AGTGTGGCGCCCCACCTGCGCGTGCTGCGTCGCTGTGGGTTCTGCCGACAGGCGTCGACG
GCTTCGGCCCGTTTGCGCTACACGCACACCTACCAACTGTTCGTACTCACCGTTACAACG
GTTTATCTTATTCAGGAGATCATCTATGCTTACCAGGCACGGAATAACATGGAGATGTTG
GCGCGCGTGATGTTCCTTCTGTTGTGTCACATCACCTCTCTCGCGAAACAGATCGTGTTT
TACGTTGATGCCGATCGAATCGACCACCTCATTATTCAATTCGATGACCCGATGTACAAC
CCAGAGGAGGCGTCGCGGCGGCGTCTGCTGGCGGCGACGGCGACGAGCGCGCGCCGCCTG
CAGCGAGTCTACTCCAACACCGCCATCTTGACGTGCATCCTATGGATCGTGTTCCCCATC
ATGCAAAGGCTCACAGGCCGCACCGTGTATTTTGCTTTCTGGACAACATTCGATTATAAT
AGTTCAACTATAGTATTCGTGGCAACGTTGTTGTACTCATTCTACGTAACGACCCTAGTA
GGCGTCGCCAACACCACCATGGACGCGTTCATGGGCACCATTCTCTATCAGTGCAAGACA
CAACTCAGAATCCTACGAATGGACTTAGAGATTCTTCCCGAGAGAGCGTATGATTTAAAG
AAACATACAAATGAACCATACGAGAAGGCACTGATGAGGTTATTTGTAAACAGTATAGAG
CATTATGAAAAGATAAGTGAGACGGCACAGTTGCTTCAGGATATATTTGGTGGTGCAATA
CTTGTCCAATTCGGTGTAGGCGGCTGGATTCTCTGCATGACAGCTTATAAGATAGTAGAT
CTGAGTTTGCTGAGCATCGAATTCGCTTCAATGATCCTTTTTACCATCTGCATCTTAACC
GAATTATTCCTCTACTGTTTTTATGGGAACGAGGTCACAGTTGAGAGCGACCGGCTGATG
TCGTCGCTGTACGCGATGCAGTGGCTGTGCACGCCGGTCGCGTTCCGCCGCGCGCTGGTG

CTGGCCATGGAGCGCGCCAAGCGCCCGCTGCGTCCTGTCGCCGGACTCATCATACCGCTC
TCTCTCGAAACATTCGTCACTATATTAAAATCATCTTATACTTTCTACGCTGTACTTAGA
CAGACAAAGTAG

>EhipOR34
ATGGAGCTGTCGGTGCGGATG
TCCTCGCGGATGTCGCAGGTGGTGTCCACGTGCGACGTGCTGGTGGTGGTAGTGACCGCC
GGCGCCGGGGTCTACGGTGCGCCTCGCCGGATGCGGGACATGCTCAAGTTCATGGAGAAC
GTCGCTTCTGTGGACAATAGTATTGGCGCTCAATATTCAGTGGTGTATGAACGCAAGTTG
TGCGCTGTCGTGCTAGCTATTTTAATCTTCTTCTCGATTCTGATTACCGACGACTTCTGT
TTCTACGCTCTACAGGCGAAGCGAGTGGACAGACAATTGGACGTGGTTACGAACTACATC
GGGTTCTATTTACTGTGGTACATCGTGATGATCTTGGAACTGCAGTTCGCGTTCACCGCG
ATCTCGATGCGCACGCGCTTCCAAGCCGTCAATGATGCGCTCACGCTCACCGCAAAACAT
GTCTCCGTGCCCTTGGACAAAACGAGCGAGCCTAATCAGCTGAACGTGTTCGCGATCAAC
GTGTCACCAGTGGAGGCACAACGCGACAATGGCAGCGTGAACCTGCTGTTGGATTCTTCG
TCTAGACATGATAATGCTGTCATTGTTAGAAAAGCTGTGAACAGCGAGCCTCGTCTCATA
GTGCCTCCATGCGAGGCAATCCGGCGTCTGGCCGCGCTACACGGGACTCTTTGTGAAGTG
GTGCAGCGTATCGACGCCAGCTACGGCCTGCCGCTCATTGTCATACTCATATCCACGCTG
TTACATCTCATCGTTACTCCTTACTTTCTTATCATGGAGATTATTGTGTCGACGAATCGC
GTACATTTCTTAGTACTGCAATTCCTGTGGTGCGCTACGCATCTGCTACGGATGTTTGTG
GTAGTTGAACCTTGTCATTACACTATCATGGAGGGTAAAAGAACCGAGGAGTTGGTGTGT
CGGTTGATGACATGCGCGCCGTCTACAGGCGCGTTGCCGTCGAGGCTCGAGCTGTTCTCG
CGCCAGCTGATGCTGCGCTCGGTCACTTATTCTCCGATGGGCATGTGCACGCTAGACCGA
CCGCTGGTGGCTTCTGATGTTATGTAA

>EhipOR35
ATGCTGCTCGGACGCTCCCTCAGGAACCTGTGCCGTGGCAT
CCCAGTCAGAGATATGCAACTAATGTCCCCAAATGAAGACGAAACTATCGGCGTCCTACT
TAGGAAATGCGTGTTACGTCACCAGGTAATTCTGGAGACAGTCCGATTATTGGAGGAGTA
CTTCTCTGCTCCGATCCTGGCACAGTTCACTGTTTCTACTGTTATCATATGTGTTACGGC
TTATCAGTTAGCCTTTGAAACAGTGAAGATGGTTCGTGTGATATCCATGCTTGCGTATTT
ATTGGATATGATGCTGCAAGTTTTCATCTACTGTTATCAAGGAAACCAACTGTCTGAAGA
GAGCACCGAAGTCGCTGGCGCCGCGTACTCCTGCCCGTGGTATTCGTGCTCCGTACGTAT
CAGAGGCGCCATCCTGGTCCTCATGACACGAACCACGAGGATCGCCAAGCTTACCGCCGG
GGGATTCACCAACCTCTCTCTATCCACCTTCATGGCTGTACGTATCACTTCGATCATAAT
CAAAGCGTCTTACACTTTCTTCACGGTACTGCAGCAAGTTGAAGGCAGGAAGCCATAA

>EhipOR36
ATGAAACCAAAAAA

TATTGTATTGAAACCTCCAGAAAAGAAATTTAACACATTTAGCGAAACATTTCGTTTCTG
CGCTTTCGCATTAGCAATTGCCTTGATATATCCCAACAAAAATAATATTATACGAAGATG
GATTACAGTTGTTTCAGTAATTTTCTTCAACTCTGTCATATTATTCTGGTTTGTGTCATA
CTTGATCAAATGTCTGATCAGTGTGGACATATACAACTTTGCAAGGGCCGTTACAGTAGG
AGTCGTTGTCCTACTTTTCTTATTCAAATCTCTTTATGTGAATTGGAAGAATGAAGAATT
TGAACAATTGTTAAATAAAATATCCAAGGATTTGCTTAAAGGAAACCACATGGATGAGGA
CTATCAAAGAATATACGAGTACCATATAAAACAGGCCAAAATTGCACAAATATGCTGGCT
AATCATACCTACAATATTAAGTCTTCAATTCCCTCTTTACGCCAGTACAGGTTTGATCTA
TGAGACTCTAAATAGTGATGTTGGTAAGAAGTATATGGTCTTTGAAATGCAACTTAAGTA
TATTGAAGACAAACAATATGTGTCACCTTATTATGAAATTATATTTGCATACAGTTTGGT
ACCATGCTTAATCCTAGTGCCTAATTTTGCTGGTTTCGATAGTTCCTTTTGTATAGCGAC
GACGCATATGCGTCTCAAACTTAAATTAATGACGCACAAAGTTCATAGAGCCTTCAAAGA
TGCAAGAAATCGTTCAGATCTACAGATGAGGGTGAAAGAAGCAATAAAAGATCACCAAGA
AGCTTTAGGATTCCATAAAGACATACAACAAGTGTACGGTGGTTGGCTACTGGCTGTTTT
TCTTTTAACATCTTTCTTAATTTCATTCAACATATATCAAATATATCTAAACAAGCGTAT
CGATCCGAAATATGCGATTTTTACTTTGAACGGTGTACTGCATATGTATATGCCTTGTTA
TTTTGCCAGCAGTCTAATTAAGGTGAACGAGGAGTTGTCGACAGATCTATACAATGCATC
GTGGGAAGATTGGGCCGATCCAGCCGTCACCAAATTACTTGTTTTTATGATGGCCAAATC
TCAACAACGTCTCGTGATCACGGGAAAAGGAATAGTTATATATAACATGGATCTTTTTAT
TTCAATATTGCATACGTCATACTCTTTTTTCACTTTGATTACTGCAAAATAG

>EhipOR37
ATGTCCGCAGGAGTTGCGTTCACATGCTACGTCCCAGCGAATGTCACAGCCCTGC
TCATTGTCATAGCTGGCTACACAGAAGCACAGATGCTAGCTTTGAGCGAAGAGCTTTGTC
ATCTTTGGGACGATGCCCAACAAAACTACTTTAAAGAAATCACTCAAAACACAAATAACG
GTAGGCATTTTGATCCTGCGGTTGAAGTAACTGACAAAAACAAAACCATTAACGAATACA
TAAAACTACATTTAATTGATATAATAAAAAGACATGCAACTAATCTAAATCTTTTAAGAC
AAGTGGAAGATGTCTTCAGAGGTGCTATCGCTGCAGAATTTGTGTTACTAATATGTGGAT
TGACAGCAGAACTTCTCGGAGGTTTGGAAAACACTTATATTGAAATGCCTTTCGCAATAA
TGCAGGTAGGAATGGATTGCTTGACCGGCCAGGGACTAATAGATGCAAATGTCAAGTTTG
AAAATGCCCTGTACGATTGTAAGTGGGAGAACTTCGATGTGAAGAATATGAAAATAGTTC
TTCTGATGTTGCAAAATTCTCAAAAGACAATGACACTTTCTGCCGGAGGTATAACTATTC
TTAGCTTTAGTTGTTTCATGTCTATTATCAGATTGATTTATTCAGCTTACACCACTTTAC
GATCTACTTTGGATTTAATGTAG

>EhipOR38
ATGAA
GCCGTCTCTGAAAATTGATTTAATTCAGTTCATCACATTACCAATAAAATCCCTTAAGTT
CTTCGGTATATGGGTATTTATACCGTATCCAATAACATCTATCGGATTTTGGATCGGCGT
TTTAGTTCGTTTCGTTGTAGGAATTTCGATTTTCATTATACCAACTGCTACGCAGTTCTT
GTATTTGGTTACTTTGATAATGTCAGGAAATGCTGAAATAAGTGAAATTGCCGGAATTAT
AAATTTAGTTCTCACCGAACTGCTGACATCGGTGAGGCTCTTGGATTTACGCTTACGCCG

GAAGTCACTTACGCAGCTGATGGATCAGCTCGTTAACATAGAATCTCATTGTTTTGTTGA
TGCCCATAAAGAGATATTGGAAACAGCCATGAAGAAATCAAGGAAACTGTACCTGTGGAT
GCTTTTCTTGACGATTTTTGATATTACGGTGCATGTCATCGTAGTGCCTGCTCTGCAAGG
TTTTCAAACATTGCCTCTGAAGATGGATTTTATAATTTTTGATGTAAATGATGAAAATTA
TTTTAAATACATTTGCGCTTATCAGATTCTCTATAAGCCAGCAATGCTAACCACATTCGT
TGCTTTACTATCATTATTATGGTCGTTTATGATGAGCATAATCGGCCAATTGGATGTTTT
AATTTACAACTTTGAGAACATGAATGGGCTTGTTGAGGCGATGAAGGCAGAGAGGCTCTG
TGATGGAAACGAAGCTTTTAAGGAAATTTTTAAAAGATGCGTCCTTCATCATCACGAGAT
TATAAGATACTTAACAACGTTTCAAAATGCTTTCGGAGGGCAAATGTTAACCACTTTGAT
TTTGAGTGCGACCATTATAGGTACTACTGCCCTGCAAATTTTATCTATAGAATCACCTAC
AAAAAACATTACCGTAATAATTTGGGTTTTACTTTTTCTGTTCATTACTGTGTTTATACT
TTTCGGAGATTGTTATTATGGAGATATCATTAGGGTTAAAAGCTCACAGTTAGCAACGGC
GGCTTTTGCATGTCCCTGGCTAAATAAGAGGAATCAGTTGAAAAAGAATCTCTTAATTTT
TATAGCCAAATGTCAGCAACCATTAATTGTGATGACACCAATATTAGTGCCAGTGACTAT
ACAAACTTTTACATTGGTAATGAATTGGACATACAAAGGTTGCGCGGTGTTGAATCAGAT
GAAGAAATAA

>EhipOR39
ATGGTCGCTTACAACTATGTCTTGAAAACAATTTTCTTTATCATTAATCGA
GAAAAATTGAAGATTCTAATCACAGAAATAAGCTATTCTGACGATAAAATCGATGAAAAA
CGTATTAAACAAATGATTATATACGTCGTGGTTATGGCTACATTGACTACTACTCTAAGT
GGAATTTTTACTTGTTTTTCTCTATATAATGGAGAAATGACAGTTGAAGCTTGGATGCCA
TTTGATCCGATGAAGAGTACATTGAATTTGTTCTTAGCAACACAAATAATAATGATAGAT
TTTCTCGTGCCGTGTTTGTACAGGGGCATTGCGATGCAAGGGATCGTGTGCAGTATAATA
ATGTATATATGCGACCAGTTGGTTGAGTTGCAGGACAGGATTAAAAAAATAAAGTATTCG
CCGGAATATGAAAGCGCTACGCGAGCCGAGTTTAAAGAGATTTTGAAGAAGCATGTGCGT
ATGATAAGATATTCCAATTTAATGACGAAATTTTTCAAAGAATATTTCTTGATACAAAAC
TTGGCAGTGACAATGGAATTGTGTGTAAACGCGCTAATGATATCATTGGTCGACTTAAAA
GAAATAACACTTTTATCGAGTTTTATGGCTTTCTTATTTATTGCATTGTTCAATGCGTAT
ATTTATTCTTATTTGGGTACCGAGTTAATTATACAAAGCCATGGTATAGCTTTGGCTGCA
TACGAATCGCCTTGGACTTCTTGGCCTGTGGACCTTCAGAAAGACTTGCTGATTGTAATC
ACGGTAGCGCAGAAACCGCTTGAATTGAACGCCGCCGGCATCACTACCATGTCTTTACAA
ACATATAGTCAGATGGTGTACAATGGATATTCAGTCTTTGCAGTCCTCAGCGATGTCGTT
AATTA

>EhipOR40
ATGGAAAATACGAAGAAAGTCCAACCATTT
GATGCATTTTCCGTGTCATATAAGATATTGATATTATGTGGATTTTTCAAACTTATGCGC
CCTACAACACGTTTCAGATATATTTGCTTTCAAATATATAGGCCACTGTCTTTCTTGATT
GTTCTGGTATTCATCGTGCAGCATTCAATTTATGCTGTTATTAAAGTGATGGATAATAAA
CTTGATAAAGCGTTAGATGCGTTTATTTTAATTCCGCCTGAATTAAATCTGTTAAGTAAG

TTACTTGCTTTGAATTTGCACAGTTCTACAGTGGACAAACTTAATGATGTTATGCGTGAT
TCTATATTTGATGCAAGGAACCAAGAAGATGAAATAATTCTTACTAAGTTTGTGTCTGAT
ATGCACCAACTAACCAAAAACGTGGAGATCGGTATGGGCGTCGCTGAAATTCTTTACATA
CTGTCGCCCATTTTCAAAAGAATATACGATCCAGCTAGTACAATAGCTTCATACTATCCT
TTTAAATTGGATAATTGGGGAAGGTATACAATTGCTTTATTAATGGATTGTTCATTTTTA
CCATGGATAGGAAACGGACACGTATCGTTAGACTGTCTCATTGGTATATATTATTCTCAA
GCAACTATTCAATTGAAACTTATAAAATATAATTTAGAACATCTTTTCGATTCAGACGAA
AGAAATAAAGATAACAGCCGGAATATGGAACATCAATATATCGATGTTGTAGATAACACT
ATACAGGAACGTTTCGAGCATTATGTTGAACGTTATTCTAAAGTCAAATGGTATTTACAA
GAACTGCATAAAGTGTTCAGCGGTGCAATTATTTACCAATTCGCTTCAACTATTATTATT
GCGTGCCCAGTAATCTACAAAATTAGCTTTATGGATTTTTTTTCCGTTCAATGTATATAT
CTAACAGTATATTTAATAATGTTAGAAATTCAAATCGTACTATATTGTTATTATGGAGGC
CTAGTCGAATACGAGAGCATCTCGCTTAATGATTCTCTTTACATGAGTGATTGGATGTCG
GCATCTCCTAAATTCCGTCGTCAGATGTTGATCGCCATGGCGCAGTGGTCGCGTCCGCTC
ACACCACGCGTCGCTGCCATAGTACCACTCTCTCTTAACACTGTTATTGCGATGCTTAAA
TTTATTTACTCTTTATACACAGCACTAATAAGTACCAACAATATAAATTAG

>EhipOR41
ATGTTGAAAAATTTTGTGTCT
AAATTAGAAAACCCAGATCATCCCTCACTAGGTCCCACTTTGTGGGGTCTCCAAGCCTTC
GGATTGTGGCAGCCGACCAAAGGAGTGGCCAATATTATTTACAATTTAAAACACATATTT
TTAGCATTATTCATTATGAGGCAATATGTAGAACTTTGGATCGTCAGATCGGATCTCGAT
TTGGTGCTTACAAATCTTTCTAAAACGGTGGTCACTACCTTCTGTGTTATAAAAGCGGGA
ACGTTCGTGTTTTGGCAAAAACGCTGGAGAGATGTCATTGAATACGTATCAACTTTGGAG
AGGCGTCAGTTGTCCGAGAAAGACGATGTAACAAAAACCTTTATTGGAGATTACATAAAA
TATTCGCGAAATATCACATACATGTATTGGTTTTTGGCCATAATGACCGTTTTTAGTCTT
ACTGTTGCACCAATACTCGTTTTCTTTTTATCGTCTAAACATCATAAACATATAAAAAAT
GAGACAATGCCTTATCCACAAATAATGGATTCCTGGGTTCCGTTCGACAAATCATCCGGT
TATGGATATTGGTTCACAGTGTTAGAGGCTACATTCGTTTGTTACCATGGAGGCGGCATC
GTTGCGACATACGACTCTAATGCTCTTGTTATAATGTCGTTTTTCGCCGGGCAATTGAAG
TTATTGAAAGCGAATTGTGCAAGATTATTCGGCGATGGCGTCGAGACTGTCACTCATAAA
GATGCGATGAAAAGAATTCGCGATTGTCATAGTCACCACCTGCTGTTGATCAAGTGTTCA
AAGATTTTGAACTCTCTTTTATCACCTGTTATGTTCTTGTACGTTATCATCTGTTCGTTG
ATGATATGTGGCAGTGCAATTCAATTAACTACAGAGGGTACGACGGGTATGCAGCAACTT
TATATAGCGGAGTTTGTTGTGGCACTAATAGCACAATTATTCTTATATTGCTGGCATAGC
AACGACGTATTATATGTGAGTGAGACAGTCCAAGAGGATGTATATGCAAGCAACTGGTGG
TCGACAAATGTGCGCACGCGCCGTTCCTTAGTGTTACTCGGTGGTCAGCTACGAAGAAAG
ATAGTATTCGAAGCTGGACCATTCACGGAGCTTACTACATCCACTTTTATTACTATTTTG
AAAGGCGCTTATAGTTATTACACTTTGTTGAGTAACAAAGGAAATTGA

>EhipOR42
ATG
TCAGGTGGTACTACTGGTCATCCTCGACGCTATTTTGCTTTACATTTTCTTCTTTTAAGA

TTTCTTGGCCTAGGTTGGTGGCACCATCCCGATGAAGGAGACACCAGCAACTTCCCCGGC
TGGTATCTATATTATTCTATCATTACAGAAGTCATATGGGTCGCAGGTTTTGTCGGCCTA
GAGTCCATAGATCCTTTCATTGGTCAAAAAGACATAGACAGATTCATGTTCAGTCTATCG
TTCGTGATAACTCATGACTTGACTTTGATAAAACTCTACATATTCTTTTTCAAAAACGGC
GACATCCAAGACATTGTACGTACCCTTGAGATAGACCTGCACCAGTTTTATCAGAATGAT
AAAATAAATCGCGCGACGATTAGAATAACGAAAATACTGACTGGAGCCTTTCTATTCTTC
GGATGGATCACTATTGGTAACACTAACGTGTACGGAATTATCCAAGACATTCGTTGGAAA
GGTGAAGTTGCCACTTTAAACGATTCTGACCTTCGACCGCCACGAACTCTTCCACAGCCG
ATTTATATACCCTGGAATTACCAATCGGACGTGTCCTATATATCCACATTCGTTCTGGAA
ACCGTAGGTTTGCTATGGACGGGTCACATCGTTATGACAATAGACACGTTTATCGGATCT
GTTATACTTCACATGAGTTCTCAATTTTCAATATTACAAGAAGCAATAACGACTGCATAC
GATCGCACAATGTCACAACTTTGTGGTAATTTACGACGTGACATCCGCGAACACAATCGT
GATCCGTTAACGACAATCGACGGCTCCCATGATATGGATGAGAACGAAAACAGAGAAAGA
TTTGTAAGAGCACGTTATAGTGAGAAAGAAATTGAATCGGCATTAGAAGAAACGTTAAAG
AATTGCTTCCGTCAACATCAAGTTTTGATAAATTGTGTGGAAAAAATTTGCCAAAACATAT
TCGTATGGTTTCATGACTCAACTTCTGTCCAGCATGGCGGCAATTTGCGTCGTAATGGTC
CAAGTTTCGCAAGACGCATCCAGCTTTAAGTCGATAAGATTGGTGACGTCGTTGGCATTT
TTCATGGCAATGATCATACAATTGGCTATACAATGTTTCACGGGTAACGAACTCACTCTT
CAAGCAGGACTGATGTCAGACGCGGTGATGGCTTGCAATTGGGAGCGGATGCCGGCACGC
CTTCGTCGAGATCTGGTTTTGGTTATGGTCAGAGCGCAACGCCCCTTGCATCTCACAGCC
GCTGGATTTGCCTACATGGATAACCGCTGTTTTCTAGCTATCATGAAAGCTGCCTATTCG
TATTATGCAGTACTAAGTCAGAAACAAGTTAGGTAA

>EhipOR43
ATGAGCGCCGTC
ACTACGTGTACGCTTTGGGCAATAATGCCGCTGTTCGACAACACTGGAACCAGGTCTTTC
CCGTTCAAAATTTGGATGCCTGTGAAACCACAGGGGTCACCAGAATACGAGTTCGGCTAC
GTGTACCAGATAGTGACCATCTACATCAGTGCCTTCCTCTTCTTTAGCATTGATAGCGTT
ACACTTTCCATGATCATGTTTGGAAGCGCACAGCTCGAAATTATTATGGATAAAGTTAAA
AAGTTGCAAGTAGTACCAATGTCAGCGAAACTCAAACTTGTAGAAAGGGACGCGCGCATC
AAAAGTAACAACGAACTGTTCACAGAATGCATTCGTCAACATCAAGCCGTAATAAAATTC
ATACAAACAGTGGAGGATACATATCACGCCAACATATTCTTTCAACTGAGCGGTTCTGTC
GCAATTATATGTATCATTGGACTGCGGATATCGATTGAAGAACGCAATAGTGTCCGATTT
TTTTCCATGGTGAACTATATGGTGACCATGCTGTCACAGCTGTTTCTCTACTGCTGGTGC
GGTAACGAGCTCACGATACGAAGCCAAGAACTTCGTGAAGTGATGTATCAGAGTCCATGG
TACGAGCAAGATATTAAATTCAAGAGAGCCCTGTGGATCGCTATGGAGCGGATGAAGAGA
CCCATTATTTTCAAAGCCGGGCGTTACATACCGCTGTCAAGGCCAACTTTTGTATCAATT
CTACGATCATCATATTCATATTTCGCTGTTTTGAATCAGACTAACAAGAAATAA

>EhipOR44
ATGAAGTACGTGGGATGTTTTCGAATACA
TTTTACAATATTAGGCATAGCCGGTATTTGGATGCCGAGATCTTTTGAAAATAACGTCAG
ATTGAAATTTTATTATAGTGTTTACAGAATATTTTTTTTGACCTTATTTTTGGTCGGCAT

TATTTATACACAATTCAGGTTTTTCATCCTGGTGATCGGTGACATCGAGAAAACAGTCGA
TTCAAGTGTGTTGTTTTTCACAATTTTACCACATATAATCAAAATCTATACGCTCATATC
CTGTCGGGAGAGAATAATTCGTCTTTTGGACATAATAGAAAGAATTCCAGATAAAAATAA
TATATTACAAATGTTCTCCAAAAATGTAGCCCTAATATCAAGTGCATACTTCTGCACCTG
TATAGGTACAGCTATATTATGGTGCGTTTATCCTTTCATGAAGCCAGTTTTGACATTACC
TTTTTACTATCCTTACATCTCTCAGGAATCTTTCTTGTTTCCAGTTTTATATGTTTATCA
AACGTTTGGAATTATTATCAATGCTCTTACGATAGCTAGCGAGGATTTTCTAGCTGGAGG
GCTAATGGCGTTAGCTGCAGCACAATTGGAATTGCTGTGTTGTGATCTATCCACCATTGG
AGAAAACAAAGAGAATATAAATGGGAACGTTGATCAATATTATGAAAAAATTGTAACATG
TATCAAATTTCACGTCAAAATCATAAGTTTTGTAGAAGAATTGTCAACCATATATGGACT
TTCTGTGTTTGGGCAGTTTCTATTTAGTGGCATACTATTGTGTGAATCAGCTTTTATAAT
TATAACGAGCGATGTATTTACTGAATCTGTAACGATGTTTCTATATTTACTATGTCTTTT
GGGACAGCTACTGCTATATTGTTTTTGTGGAAATATGATAAAAACTAACAGTGAAAAGGT
AGCAGCGGCGGCTTATAGCAGCAGATGGGAGAGAACCTCTTTGGCGACTCAGAAGGCGTT
GTTGTTTCTCATCATGAGAAGCCAAATGACACTCACCGTTACCCCAGGAGGAATATTTGA
CTTGTCTCTAGTTACATTTTCTGCTGTTTTAAAATCTTCATATTCGTTTTTGGCAGTACT
TAACCAGAAGCACGATTCTTAA

>EhipOR45
ATGTCTGAGTCTCCAAGTGAACTCGCAAAACGTGAGA
TAGATGAATCTCTAATACTTTGTAAGTTCTGTATGCGATACATAGGTTTATCATTTGAAG
AACCGAAAAGCACACGGGGATATCTTACTCAAAAACTAATGTTCTTACTATCTGTGTGCG
CTATTTTCTACCACGTCTTCAGTGAAATCGCTTACATAGGTCTCACGTTGTCCAATTCGC
CACGAGTCGAGGATGTTGTTCCGCTTTTTCATACTTTTGGCTACGGCGCTCTGAGTATTG
CAAAAGTGTCTGTATTATGGTACAAGAAAGAGAAATTTGGAGAACTCCTGCAAGAGCTGG
CAGGAATCTGGCCGATGCCTCCACTCGACGAAGCTGCTCAAACCACCAAAAGCAAGAGTC
TTTCCGCTTTAAGAATGGCACACCGATGGTATTTTACAACTAATATGGCGGGTGTGTGGT
TCTATAATTTAACACCAATTGGTATCTACTTCTATCAGTCGTTACAAGGACAGAATACTG
AAGTCGGCTATGTTTGGATGTCCTGGTATCCATTCGATAAACACGAGATGATAGCACACG
TTGCAGTTTATATCTTCGAAGTATTCGCCGGTCAAACGTGTGTATGGATTATGGTTGGCA
CTGACCTACTGTTCTCCGCTATGGCGAGCCACATTGGACTCTTACTGAGACTACTACAAC
GTCGTCTAGAGTCACTAGCGACGACCCATCAGACAGATCAGGAATATTACCAGGAAATCG
TATCCAATATCAGGCTGCACCAAAGGCTTATCATGTATTGCAATGATTTAGAAGACGCTT
TTTCTCTATCAAATCTTATAAATATAGTGATGAGCTCTGTTAATATATGCTGTGTCGTAT
TTGTAATAGTGCTCTTGGAACCGTTCATGGCTGTTAGCAATAAACTGTTTTTAGGTTCAG
CTTTGATTCAAATCGGTATGCTGTGTTGGTATGCGGACGACATTTTTCATGCGAACTTAG
GCGTGTCCATGGCCGCATATAACAGTGCATGGTATAAAACCAGCCCTCGTTGTCGTCGAG
CGCTACTTTTCCTTATCAAAAGATCTCAAAAACCTATTGCGTTTACTGCAATGAAGTTTA
CTAATATTTCACTCGTTACTTATTCTGCGATACTAACCAGATCTTATTCATATTTTGCTC
TTCTCTATACTATGTATAGCGAACACTAA

>EhipOR46
ATGTCCGAGTTAGAA

AAGTTAAATCCGGAATTTGTTAAATCCTATCGAATGTTATTATTGTTTCTAAGATTTAAC
TTCATCGACCTAATGAATCAAATGAGTGATATGCCGTATTTTAAATACTTGTATTTATTT
ATGTTTTCAATATATACTATTGCGTTGGTATGTCATACAAGTGGCATATTAGAAAAGATC
TCTGAAGGGGCTAACTTACTTCAGCTATCAGGGGATTTGTCAGCTGCTGTGGTTATTTTA
CAAGGGGCAATATTGTTTGTGAACATTTATTTTAATAAAAATAAGATTAAAGAATTTATA
CTGAGACTTGGGTTGGAATGGCGCGACGACAACAATTTGCGTCCAGAACTTGTGATGCTA
AAGCGTAAACAACTAAGTGCTGTTTACCTGTTCACTTTTGCATTTTACACTGGTTTAAAT
ATCTTTGTGCATTCCTATATGATGATACCATTGATTTATATAATGATTAAAAAATTTATA
TTAAAGCTCGAAGTTTCGCACACAGTGCCTTTTTATGCAAAAGTGCCTTTTAATTACGAG
AACAACTTTCTTTTGTACCTCATTGTTTATATGTACGATTATGGAATTATGTATAACTTG
GGATATTTAATATCTGCAGATATTCTACTGATAACAGTCAGTATGAACAATTTACGAATA
CTGTTTCAATTGTTACGAGATGATTTTAAAAGTATTTTTAATTCGCTTAAGAATGGAAAC
ACTCCACGTAGCCACGGTGTGATAGAGAAACATTTAATTACAACTATAAGAAGACACATA
ACTTTATTAGAACTGATGCAAGAACTCAGTACTGCATTCAACGGCATCTTCGCAATCCAT
CTTATATTCGTCTCAGGGACGATTTGTTTCTTTGGATTTGCAGCTGTGTTTGGCGGCACT
CTGGAGGATTATAAAAATTTCGTAGCTTCAGTAATCGTCATTGTTTATATATTTTTCTGC
TGTTTACACGGACAACGCTTAGCAGATTCGGGCATGGATATTGCAACTTCCGTTTATGAC
AGTGCGTGGTACCAGTTACCAACTAAATACAAAAAGATTATATTAATTATTTTATTGAGG
TCACAGAAAACTTATTACATTAAGTCGACTCGCTTTACGGAGATTTCTTTGAAAACATTT
ACAAGGATGATGAATATGACATGGACATTTTTCTCTGTGATGAAAACCATCTATAAAAGA
TCATAA

>EhipOR47
ATGATTTTTCAAAGAGTAATTTCGT
TCGCTAATGGACTCGAAGACCCTAAACATCCATTATTGGGACCAAATTTGAAAGCCCTTT
ATGTTTACGGCCTTTGGCAATCTGGAAGCAAATTTCGCATCGCTTGTGCTAATTTCATAC
ATTTTCTAGCGTTCATTTTTGTCTGCACTCAATTACTCGAACTTTGGATTGTGAAAGACG
ATTTTATGAAAGTACTCCATAATTTGTCCGTAACTATTCTCAGTATTATTTGTCTCGTAA
AGAGTTCCTCTTATATATTATGGCAATCGCGATGGAAGGAACTTGTAAATGCTATATCAA
CGGAAGAGATATCGCAAATGACGCAAGAAGATAGTGTTACGCAAAAACTAAGAAAGAACT
ACACGACATACGCAAGGGTTGTGACATACTTATATTGGTATCTGGTTGTGATGACGAATA
TAACAATGATATCTTCACCGCTGTTAAAATACGTAACCACTGCAACCTATCGTGAAGAAA
TAAGCAATGGAACAGAGCCATATCCATTAATCATGAGCTCCTGGTTTCCTTTTGATAAAA
CTAAGATGCCTTGGTATTGGATTAGTGTTGGAGTGCATATATTGATGAATATTCACGGTG
GTGGTATCGTCGCAGTATATGATTCGAATGCTGTTGTTATCATGATCTTCTTGAAAGGTC
AGATGAGGATATTGCGAGAGAAATGTAAAACATTGTTTAATGATAGCGAGAATATTCAGC
GAAAGGATATTTTGGATAGAATCAAGGAATGTCATAGGCATCATGGCTTTCTCTTGACAC
AAAGTGGTTTGTTTAATTCCGTTCTATCACCGGTTATGTTTCTGTATGTTTTGGTGTGTT
CTATAATGATATGCTGTAGTGTAGTGCAATTCCCATCGGAGCAAGCGACTACTTCTCAAA
AATTGTGGGTATTGGAATACACGACGGCGCTCGTCTCCCAACTTTTCTTATACTGCTGGC
ATAGTAATGAGGTCCTTGCTGAGTCAAATGAGATAGACCGTGGTGTATTTGAAAGTGATT
GGTGGAAAGCAGATGTGCGCATACGCAAACAAGTAATATTGCTTGCAGGGAAGATGGGTC
AACCCTTTTTGTTGTCTGCGGGGCCGTTCACAACTCTATCTGTGCCTACATTTATAAGTG
TCATCAAAGGCTCTTACAGTTTCTACACTTTATTCACACAAATGCATGAAAATAAATAA

>EhipOR48
ATGAGATTCTTAGATTCAAGAAGTAGGCCTTTTAAACGAGTAGACAGACGTAAG
AATATTTTGAAAATGCGTAACACGAGTTGTCTTAGATTATGTCTAACCATAATGACAGTA
ACCGGAGTATGGCTGCCGACGGCCTTAAAATGTAGCCATTTAAGATATATTTATTATATC
TTCGCATTTATGTATCACCTGCTCTTCGTGTTACCTATTATTTATATGGAGTTCGGTGTC
TTATATCAAGTATTGGGAAATATAGAACAAATGGCAAATACCAGCTTACTTTTAGTAACG
CATATCGCACAGTGTGTTAAAATATGCGTGATGTGGTACAAACAAGACGAAATACATAGC
CTTCTCGTGACATTAGATAGTTCTACTTTTACCAGAGAAGACTCCAACAAAAAACAAATA
TTAAAGGACGTCATACAAACTACATACACCGTCAGCAAAGTATTTATTTCTCTCGTTGTA
ATCACGGGAATATTCTGGGGCATTTACCCTATGCTTAAGCCTACTTTAGATTTACCCATC
CAATACCCTAATATACCACCTGAATATAAAATATTCCCATTTGTTTATTTATATCAAATT
GTAAATTTAACAATTACGGCCATCGCTATAGCTTCAATAGATTTTCTTGTTGGTGCATCG
ATGGCTTTAGTTTCCGCTGAAATGGACATCCTTAGCTACGAATTATCACTTGTGGGAAAA
ATTAATAAAAAGGACGATAAGGGAAACACAGATATTAAACGAGACTATGTAATCATTATT
TCATGCGTGGAATTCCATGAAAATATAATAAAGTTTGTAAAAGACTTGGAAAAAAATATTT
GGCTTTCCAGTGTTTTTTCAATTCTTTACAAGTGCTATTATTATTTGCGAAACAGCCTTC
AGAATCACTAATTCTACTGAAACAATTGAGTTACTTGCGATGGTGCTTTACTTCATGTGT
ATTATTACTGAACTTTTAATGTATTGCTACTATGGAGACCTTTTAAAACGTAAGAGCGAA
CGAGTGGTCGAAATGGCGTACTGCTGCAACTGGGAGAACACGGACGTGCGCACCCAAAGG
GCGTTGCTGCTGCTCATGCAGCGCGCTCAGAGGACTCTCGTCCTCAGGGCGGGCAACATG
TTCGAGTTATCCATGATGACCTTCTCTGCTATATTGAAGACATCCTACACATATTTTACT
GTGCTGAATGAAAGACGTAAAGCTTAA

>EhipPR3
ATGAATACTTTAATATCTGCAAGTGAAATCCTTGCA
ATTAAACATTTAAAGATAATGAGATTCATCTTATCATCAAATGGCTCATGGCCTGGCGAA
GTGTTAGGAGAGAAAACACCTATTATTTTGAAATGTTATAGATTTTATATTCCTCTCCAA
GCGCTTGGTCTCCTTATAGCACAAGTGTGTTATTTAGTTAAATACTTTAAGGTGTTGAAT
ATATTCTCAATGGGACACATGTATATCACGACCTTCATAACTGTGTTGATATGTATTAGA
GGAATAACAGTATGTTTAAAAAAATACCGCGATATATCACGACAGTTTTTGATGGCATTT
CATTTGATTCATTTCAGACACAAAAGCGATTATCATGAGAAGATTTACGGAATTGTCGAC
AAGGTATCATATTATTTCACAATATACTTATTGTTTATAACATTGTGCGCCGCGTCCTTA
TTTAATGGGTTGCCTGTTTACAACAATTATAAGATGGGCGCATTTACTGGCGAGAAACGT
GAAAATATTACTCTTGAATTTTCCGTATATTATTACTTATATCCTGGATTTAACGCCGAA
GATTATTTTATAGTGTGCACTATTTACAACTTCTATTTATCACTTACTACTGCAATATGC
ATCTGTTTCTTGGACTTATATCTGTCTATAATGATATTTCAAATAATTGGACACATACAA
ATATTAAAAAATAATATCGAAAATATAACAAAACCGAGAACGGTTACTGGAAAAACTAAT
AGAATCGATGATACCGAGCCGTCTTATAGTATGCCATTCAGCGCTGAAGAAAATGATGCC
ATTCATACCAAATTAGTAGATATCGTTCATCATCACAGACTTATAGTCAATTTTACAGAT
GATATATCGAACTTTTTTGGTCCGGTGTTAGCTTCCTACAATCTGTTTCACCTTGTCAGC
GGCTGTCTATTGCTTTTTGAATGTTCGCGAGGTGGCGACGCCTTAGCTCGCTATGGACCT
CTCACTGTCATACTTTTCGGACAACTTATGCAAATATCGATAATCTTTGAGATAGTTGGT

TATATGAGTGAAAAGTTGATATATGCCGTATATTGCACGCCTTGGGAAAGCATGAATATC
AGTAACCAGCGATCAGTATGCATCCTACTCCATAGAGTACAAACTCCCATACAAGTTACA
GCTATGGGAATGACACCAGTCGGTGTACAATCCATGGCAGCGATAATTAAGACTTCGCTG
TCTTATTTTGCTTTTCTTCGTTCGATGGATAATTAA

>EhipOR49
ATGGAA
CAGTTTCGCCAAATTGACTGTTTTAATATAAACATGAAGTTTTGGAAATTGCTTGCAATC
TGGCCAGACGGTGACACTTGTCGTTATTACGGCTTTTACTCAAAGACTTTCGTCTCATTT
TTTGTCATATTGTACTATATATTACTCACAATAAATTTTTATTTCTTGCCAAGACATTTG
GACAATTTCATAGAAGAAATGATATTTTATTTCACCGAATTGGTGGTTGCGGCTAAAGTT
CTCACGTTTCTTTTCATGCGTAATAAAATTATAGAGATACTTAAGACATTGGAAAGTGAC
ATGTTCCAGCCTAATATTCCCTATGGGTTCGATATAATACTTAAAGCTAAGAAATTTAAC
GTAACATACTGGAAAATTGTAGCGATAGTGTCTTTTGTATCTAATGTGACTCATTTGTTA
TCCCCATTAATTATTCATTTAATTTTCTCAGCGAATCTCCAATTACCTATATGTAGTTAT
TCTTTTCTATCTAAAGAG

>EhipOR50
ATGGATACAGAAGTAGTTCAAAAATTGGGATGCAAAGATAGTTA
TTACTTGTTAGAATTAAAGAAATCTATCACACATTTCGATGAAGTGGGCAAATTCTGTGC
GCTAATACAACAAGTGTTTAGTGTTGCACTGTTTTTACAATTTAGTATGTCCTCCTGTAT
CATATGCGTATGTCTTTTCAGATGTACATTGCGCGCGCCCTTGCAATATTATATATTTTT
AAGTACTTATATGTTCATCATGGTTATCCAAATAATGGTTCCATGTTGGTTTGGCACACG
AGTCATAGAGAAGAGTTACCTGTTGACTCGCGCGATCTACGACTGTGATTGGATATCTCG
ATCGCCTCAATTCAAGAGCAATCTACGATTCTTCATAGTGAGAGCTAACCGACCGCTCTC
AATCACTGGCGGAAAAAATGTTTCTTCTTTCAGTAGCTACATTTACTTCTATAATGAATAC
TGCTTATTCCTTCTTTACATTGCTTCGTCATATGCAGTCTCGATAA

>EhipOR51
ATGATGAAGAAGTTTGTGT
CTAAATTAGAAAATCCCGATCATCCTTTACTAGGTCCCACTTTATGGGGTCTCCAAGCCT
GGGGATTGTGGCAGCCGAACAAAGGAGTGGCCAAAATCGTTTACAATTTAAGACACATAC
TCTTATCATTATTCACTCTGAGCCAATATATAGAACTTTGGATGGTCAAATCGGATCTCG
CTATGGTGATCATAAACCTCTCTAAAACGATGCACACTACCATTTGTGTCGTTAAAGCGG
GAACGTTCGTGTTTTGG

>EhipOR52
ATGG
CTTTGAGATCACAGCGCTCACTCGGAATCACCGCTGGCGGAGTGTTTCGAGTCTGTCTGC
CAACTGCCGCCACTGTATGTACTTGCAAACTGTATATTACGCTTAATCAAAAGTTTTTGC
AGTACAATTACAGTTCTGCCCGAAGAACCTTACTGGAACGAGATAAAAATAATATGTAG

>EhipOR53
TTGTCGCTTTGTAAA

TTCAGAACAGATATCAAGAATGTAAGAATACCTATCTATACATATTATAATACATTACTT
TTTTTAAAGGATCACACTTATTGCTCTAGTCCCATACACGGCTACCTGTACTATTATGGG
AACCAAAAACAACGGATAACTAAACATGGACGGCAAATATTTGTCTTTCAAACA

>EhipOR54
ATGAGACAAATGTACATTTTATTAAATTTATCTGTTATTCTGTTTGTTGCTTCAGAATGTGTTGACATAA
TATGGTTCGTTAAATCCAACATCACTTTACTTTTGCACAATTTGAAGATCACATCACTTGAAACTGTTAG
TGTATGCAAACTCAGCACATTCCTCGTTTGGAATAAGATCATTATATACGTCACCGAATCGGACGTGATG
CAGCGAAAAACGGATGATACCTTCAAGAAAACCATCATTAAGAAATTCACATAG

>EhipOR55
ATGTGTCTATCGCAAATATT
TCTGTTGTGTTACTTTGGAGATTTGCTTTTGAGATCGAGTACGGAAATAAGCGGCGCCAT
TTACAATTGTCAATGGTATAACACCGATATACAAATGAAGAAAGATCTGCTTTTAGTTCT
TAAGAGGTCTCAGAAGCCTTGCAAGCTAACAGCCGCCAATTTTGCAGATCTTAACTTGAG
AGCATTCACTACGATATTGAGCAGATCGTGGTCCGTTTTTGCTCTCCTGAAAAGTATATA
TAAATAA

>EhipOR56
TTGTTGGGTTCCGTTCGACAAATGGTCGAAT
ATGGATACGCTGCCAAAGCGTTGGAGCATTCGTTAATTTGTTTCTATTGGGCCGGCTTCG
TTGTGACGACGACGTACGACTCTAACGCTGTTGTTTTAATGTCGTATTTCGCCGGACGAT
TGAAAATGTTAGAAGCGAATTGGGCAAGATTATTCGGCGATGGC

>EhipOR57
ATGTTATTAACTAGTTTT
ATCAAAATTATAATATATATTTCTCTATTTTTTTCAAAATCTATATATATTAAAATTTTA
TCCAGCTATTTCGGTTTTCTGATCTGGCGATTCCTAAACATGATACGTAAATTCGGCTTG
GAGCACTGTGACCTGCCAACCATGCTGTGGAATGTATCGTTCCTTCTAAGAGCTCTCACG
TTGAACATTGATAGCCGTTATAAAAAACGTAAGCATTACCATCTACTTAATAAAACGAAC
ACTAACTAG

>EhipOR58
TTGCACATTTTTATTCTGTACAAAATGAAGAAAATTCTCAAAAATGAA
ACTACTGAGGTCCAATCACCAAAAGACCAATTTTTTTATCGGGCACTGGCCGCGCTAATG
ACATTCATGTGTTTGGGAAATCAAGTCTGGTGGGGTTACGAGCCTTACGGAAAAATTTTC
AAAATCAACAGATTTATAGTAACATTTACCGGTCCAATCATGGCCATCTCGCAGTTTATC
TATCTATATGTATATTTCAACCAATTAACTGCGGATGCGCTTTCTATCGTTTATTGTATG
TTACCTGTGACATTATTAGCGAATATAAAGATTAGACTAGCCAAAAGAGATATTTATAAA
AACCTCATGCTGGACTTTATGACGAAAATTCATCTCTACAATTACAAGGGCGAAGAATTC
ATAAATAAAACTATAAAAAAAGTGGAGCGTTATAGTCACCAGATGGGGTATTGCTTGATA

GGAATTGTCGCTTTCGACTCCTTGCTGTGGTGTATAGTTCCAATTATCACTAATTTGATT
CACGAGGAAGCTATCAAAAACAGGACCATG

>EhipOR59
AATGATATCTCTCTAGCATTTTTGCCAAAAACGAACGTTCCCACTTTTACCACGCAGAC
GGCATTGAGCATCGTTATAGAAAGATTTCTAGGCGCTGAAGCAAGGTTTGATTTGATAAT
CCAAAGTTTAACACATTGGTGTAGAACGAATGTATGTATTCCATTGAATATTATATTGGT
CACTCATTTGTTCCATTGCCATATTCCCCAGGCTTGGAGACATGATAACCGACCGAGTAA
GGGATGAT

>EhipIR75q2a
ATGAAG
AATATTTGTTTAACTGTTTTTCTTTTAATCGTTATAACTAGTTGTCACGCGGAAACTGAT
TTAGAAGTAATGATGATTGCCAACGTCATCCACGCTATGGAAAGGCCATCGGCCGTAATC
GCCACGCTCTGCTGGCCTCTTCACAAAAAAGTTCAACTGTACTCAATTCTCGCTGGAGAG
AATGTTGTCCAAATCAACATGATGCAAATTCTCAAACCGGGCCATATCCCACAGCGTCAT
TCTCAAGACCAGCATATAGTATTCTTAGTAGACCTTGGCTGTCCAGATATTTATAAATAT
TTCGTAAGGAGTAAAATAGAAAATCATTTTCGTTCGCCCTTCCGATGGGTTATCATTGAT
GGTTTAAACAATGATACCCACAAATCTATAATACCACAATCTTTAAGTAACATCGATGTA
CTGCTTGATGCTGAAGTTCTAATTGCTCGTCCTATTGATAATAGTACTTATAACTTACAC
CTCGTTTATAAAATAAGTCATACAAATAATTGGAAAATAGAATTTTACGGAAATTGGAGC
ATAGAGTACGGATTTCAAAAGGCATACCAATTGATTGATTCAGCTGCTTTACGGAGACTC
AATTTAAATGGATATGAAATTAAAATTTGTTATGTTCTTACCGACAATGACAGCATTAAC
CATTTAACCGATGGAGTAAACGATCACATTGATACTATTACCAAAGTGAATTTCCCCACG
ACAAATCACTTACTCGATTTCCTCAATGCGAAGAGGAAATATATTTTTGTGGAGACGTGG
GGTTATCGTGTCAATGGTACCTGGAATGGTATGACGGGGTACTTGGTTAGAGAAGAAGTC
GAAATTGGAGGTTCGCCAATGTTTTTTACAAGTGAACGTATAGCAATAGTCGATTACATA
TCAAGCCCGACTCCTACACGTTCAAAATTCGTTTTTCAGCAACCAAAATTATCTTATGAA
AACAATTTGTTCCTTCTTTCATTC

>EhipIR75q2b
ATGTTTTTTACAAGTGAACGTATAGC
AATAGTCGATTACATATCAAGCCCGACTCCTACACGTTCAAAATTCGTTTTTCAGCAACC
AAAATTATCTTATGAAAACAATTTGTTCCTTCTTTCATTCCGTTCTACGGTTTGGTACAG
CACTTTAGGCCTAGTCATCTTACTGTTTCTTGCATTGTTTGTCGTCGCGATGTGGGAACG
TAAAAGAGATCAATTGAAAGAGAAAGACGCTGATATACTAAGACCAAGTCTTGCAGATGT
AGCACTTTTAATTTTCGGAGCAGCATGTCAGCAGGGAAGTCCAGTTGAACTTAAAAGTTC
GTTAGGTCGTGTGGTAATGCTGATCCTCTTCCTGACGCTCATGTTCCTGTATACTTCGTA
TTCGGCCAACATTGTCGCTCTGCTACAATCTAGCTCATCACAAATAAGAACTCTCGAAGA
TTTACTTCATTCTAGAATTAAATTTGGAGTGCACGATACTGTTTTTAACAGATATTACTT
CTCGACTGCAACTGAACCAATTAGAAAAGCTATTTATCAAACAAAAAATAGCACCTCCTGG

CACAAAACCTCAATTTATGACAATGCAAGAAGGAGTTAAAAAAATGCAACAGGTCAATTA
TTTATACATATCATTATGTCCTACTTACATATATGTATGA

>EhipIR8a
ATGGAGATGTTCAGTTTATTTCTATTAATTTTACTTCTC
AATTTGGGTTGTGTCGCGTCGGAGTTGAGTTTACGATTTGTATTCATAATAGAAGTTCAT
GAAACAGACGTTGCTCAACAAATTGGTAGAGCGCTGAAGGCCGCCGAGGAGAAGATCCCA
GAACTCAGGGTTGACGACTCCATAGTTCAGTTGGACAGGGAGAACGAAGATCAGAGTTAC
AGAATACTATGTTCAGCTCTATCCAAAGGAGTGTCGATGGTAATCGACCTGTCCTGGTCT
CCATGGGAAATGGCTGAACAGTTGACTTCAGACACAGGCTTGCCTCTGGTGCGGACGCTG
TTAGGATCGCAGCAGTTGTTGGTGGCATTGGACGAGTATTTGGAGACGAGGAACGCTACT
GACGCAGCGCTACTGCTGGAGAGTGAGGGTGATGTGGACAGAACTCTGTACCAACTGCTC
GGTGAGTCCAATATCAGGGTGTGGGTGCACGCAGGCCTCACCAGAGACTCGGCGCGAGCT
CTGAAGACCATGAGACCTGATCCCAGCTTCTATGCTATCATTGGTAGTGCTGCATTCGTC
ACCGACACTTACAGACGGGCGGTAAAGGAAAAATTGGTGCGTCGTGATTACCGCTGGAAT
CTTGTTTTGACGGATTATTCTGGAGCTAACTTAGATGTATCCGAGCTGGTGTTGCCTACG
ATGATATTGCACGTGGATCCAAGCGAATGCTGTAAGCTGATGGGACGCAGAGATGGATGT
ATGTGTCCCGTGGAACTTGAGAGGAAACAATATATACTCAATTCTCTCATACAATACATA
TCGGAGACTTACACCAAACTGGAGAAGGATCTCTCCCTGATCTCCATCAGGGTTGACTGT
GATAATGTCCAGGGTGATATGAACGGAACGAGAGATAGACTGTTCAGGCATTTCGCTGAA
GACGCCGATGTAAATAATGAAACATTGTTTTATTGGGACTCCGATAGATCAGGACTATTC
CTTCGCTCTCGGTTTGTCCTCTCCACTTACAAGCCAGACGAAGGTCTTCAGACAGCTGCG
ACCTGGTCTGCAGATGAGGAATACCGCCTACTGCCAGGTGTAATCCTGGAACCGCTCAGG
TTGTTCTTCAGAATTGGTACTACGGCGGCAGTACCATGGACTATGGCCAAGTTAGACCCG
GAGTCAGGGGAACCCATGTATAGTGAAGATGGCCAGCCATTGTACGTGGGTTACTGTGTT
GATCTGATTGAGAAGTTGGCGGAGCAAATGGGGTTCGACTTTGAAATAGTAACGCCAAAA
TATGGTACTTTCGGTAGAAAACTACCGAATGGTACATGGAATGGTGTGGTCGGAGATTTG
ATGAGAGGTGAAACGGATATGGCGGTAGCTGCATTAACAATGACGGCAGAGAGAGAGGAA
GTGATCGATTTCGTCGCACCATATTTTGAGCAGACCGGGATTCTTATAGTGATCCGCAAA
CCCACTCGTAAAACGTCACTTTTCAAATTCATGACGGTACTGCGTACCGAAGTGTGGCTG
AGCATTGTCGCGGCGTTGGTGTTGACCGGAGTAATGATCTGGCTGTTGGATAAATACTCT
CCTTACTCAGCCAGGAACAATCCTACGGCGTATCCGTATCCGTGCAGAAAGTTTACCCTC
AAAGAGAGTTTCTGGTTTGCCCTAACCTCATTCACTCCCCAGGGCGGGGGCGAAGCGCCC
AAGGCTTTATCAGGAAGGACTCTAGTCGCAGCTTATTGGCTGTTCGTTGTGCTGATGCTG
GCCACCTTCACTGCCAACTTGGCTGCTTTTCTAACTGTGGAAAGGATGCAGACGCCAGTG
TCGTCATTGGAGCAGTTGGCGAGGCAGTCTCGTATAAACTATACAGTCGTCGAAGCATCC
ACCATACATCAATATTTCATCAATATGAAATTCGCTGAAGATACTCTTTACAGAGTCTGG
AAAGAGATAACTCTGAATGCCACATCAGATCAGGCGCAGTACAGGGTGTGGGACTACCCC
ATAAGAGAACAGTATGGACACATATTATTAGCCATTAATGCTTCTGGTCCAGTTCCAGAT
GCAAAGACGGGATTCCAGCAGGTCAACGAGCACACGGACGCGGATTTCGCTTTCATACAC
GATTCAGCTGAGATAAAGTATGAAGTTACAAAAAATTGTAACTTGACAGAAGTCGGCGAG
GTGTTCGCTGAACAGCCGTACGCTATCGGAGTCCAGCAGGGCTCCAGGCTGCAGGAGGAT

CTTTCCAGAGCACTGCTGGAGTTGCAGAAGGAAAGGTTCTTGGAACAATTAGCTTCCAAA
TATTGGAACGAGTCCGCGAGGCAAGCTTGTCCTGATGCTGACGAGTCTGAGGGTATTACT
TTGGAGAGTTTAGGCGGGGTCTTCATAGCCACCCTTTTCGGCTTGGGGTTGGCAATGATA
ACTCTCGCGTGGGAAGTGTTCTACTACAAACGCAAAGAGAAAAACAAAACACAAACCATG
GACGCAAATATCGAGAGGCGACAAGCCTTTGTCGAGCCGAAGAAGAAGGAAAAGAAACAC
GGTGTTGCCAGACTGAGGAAAAGGGATAAAAAAGTTGGCAACATTGGCCGTAGTGTCACG
ATTGGGGATACTTTTAAACCTGCTGTGAATAAAAGTAATGTTTCTTATATTAGTGTTTAT
CCCAAAGGTGGATTTCAACCTTAA

>EhipIR41a
ATGTTGACACTGC
AGCCAAACGTGTTTCATATTGAATTACTACTTCACACTATCCTTAATCAATATTTATACA
ATTCTTACTGTGTAACTTTCGTTTCAGAAATACCACTAGATATTAACTTTTCCATTACGT
TCACATGTATTGTTCCCGATCCGGAAAATCTCACGAATCAACTGTTGGAAGTATCCGAAA
AAGGTTGTTCTGATTACGTTGTGCGAATGAAGGAACCTCAGGAGTTCATGAATGCATATG
AGAAAGTCAATCATTTGGGAAATGCCAGACGCAGTGATAAAAAACTAATATTTTTGCCAT
TTCTTGAAGATGATAACACTACAAATGAAACTATCTCGCCGTTGTTAAATCTGTTATCAA
TGAAGGAAACTAGCTTTGTAGCAAATATTTTATTGTTGATTCCTTCTAGAGAAACGACAG
CAGAATGCAAATTTTATGATATAGTAACACACAAATATGTCGGACCAGACAAGGAAACTA
ATCATCCACTGTATTTAGATCGATGGAACTCTTGTACGGAAAAGTTTGTCATGAATGTAA
ATTTATTTCCTCATGACATGTCGAATTTGTATGGCAAAACGGTAAAGGTGGCTTGTTTTA
CATATAAACCTTATGTTTTGTTGGATCTCGATACACCAACTGGTCGTGATGGCATGGAAT
TGAGGATTGTAGAGGAATTTTGCAGATGGGTAAATTGCACCATGGAAGTTGTAAGGAATG
ATGCTCACGAATGGGGTGAAATTTATGACAATCAAACTGGTGTCGGTGTACTTGGAAATG
TTCTAGAAGATCGTGCAGATCTTGGCATTACCGCTCTCTATTCTTGGTATGAAGAATATG
TTGTTCTGGATTTCTCTGCTCCGTGTATAAGAACTGCTGTAACCTGCGTAGCACCGGCTC
CAAGGTTACTAGCAAGTTGGGAATTGCCGTTAATGCCCTTTACTTGGCATATGTGGATTG
CATTAATTTTCACTTTTCTTTTCTCTTCTGTCGCTCTTATTGTTGTAAAAGGATTTTCGT
CAAAGAATGTATTTATAACTACATTTGGAATGATGGTAACGCAATGTCAGCCGGAAGTAA
GGGCAGATTGGCGGGTTCGTAGCATCACAGGTTGGTTGTTGATTACTGGTTTAGTCTTCG
ACAATGCTTATTCAGGTGGTCTTGCATCCACTTTTACTGTGCCTAAGTATGAAACATCTA
TCGATACCGTTCAAGATATAGTCGATCGAAAAATGGAATGGGGAGCCACTCATGATGCCT
GGACTTTTTCGATCACTCTTTCAACAGAGCCTTTAATAAAACAATTGGTTAGTCAGTTTA
AAATTTATTCAGCTGAAGAATTAAAACGGAAGAGCTTTACAAGAAATATGGCTTTCAGTA
TAGAAAAATTACCAGCAGGTTACTTTGCGGTAGGCGAATACATTACGAAAGAAGCAATGT
TAGACTTGGAAATAATGTTAGAAGATTTTTATTACGAACAATGTGTAGTAATGTTGCGCA
AGAGCTCCCCGTACACGTCCAAGATCAGCGATCTTGTAGGGCGGCTGCATGAATCTGGTC
TCATGTTGGCTTGGGAGACACAGGTTGCTTTAAAATATTTAAATTACAAAGTGCAACTTG
AAGTTAAGTTATCGCGTTCCAGAAGAGACGTCGATAATATAGAGCCTTTAAGTTTTCGAC
AAGTTGTGGGAATTTTCATCATATATTTAATTGGTGTTTCACTTTCTATAGTCATATTTG
TGGCAGAGATGTGTATTAATGATAAAAACAAGAAAAAACTTAATCAGTAA

>EhipIR25a

ATGGGACCACATAAAGAGATTCTATCTTCATTGATGCTATTAATATTATTATTT
ATTCGAAACTCATTCTCTCAGACCACTCAAAATATAAATGTCTTACTGATCAATGAAGAA
AATAACGCTTTGGCTGAAAGAGCTTTTGAGGTTGCAAAGGAATATGTAAGGCGTAATCCA
AGTTTAGGTCTAGCCGTAGATCCAGTCATCGTAGTTGGTAACCGGACAGACGCTAAGGCT
TTTTTGGAGAACGTTTGCAGAAAATACAACGACATGTTGTCTGTAAAGAAAACGCCACAC
GTGGTCTTAGATTTTACTATGACCGGTGTCGGTTCAGAAACCATAAAGTCATTTACGGCA
GCTTTGGCTCTACCCACTGTCTCCGGTTCGTTTGGGCAGGCCGGCGACTTGCGCCAGTGG
CGCAACCTCAGCCCAAACCAAACGAGGTTCCTGCTTCAGGTCATGCCACCAGCGGACATC
CTACCGGAGGCAATAAGAGCGATAGTCACTAAACAAGACATCACAAGCGCTGCTATCATT
TTTGACGAATTTTTCGTCATGGACCATAAATACAAATCTCTTCTCCAAAACATTCCGACG
CGACATGTCATAACTCCGGTGAAAAGTTTTAATAAAGACGAGATAAAATCGCAATTGAGA
AGTCTGCGGGAATTAGACATTGTCAATTTTTTCATAGTCGGTAGTTTGAGAACTATAAAG
AACGTTTTAGACGCGGCCGATGATAACCAGTACTTCGGTAGGAAAACGGCATGGTTCGCT
CTTTCTTTGGATAAAGGAGACATCAACTGCGGATGTAAAGACGCCACAATAGTCTATATG
AGACCGACGCCTGACGCCAAGAGTAGAGACCGTTTGGGGAAGATCAAAACCACGTACAGC
ATGAATGGAGAGCCTGAAATCACATCTGCTTTCTACTTCGATCTGTCGCTAAGAACATTT
TTAGCTATAAAATCGCTACTGGACTCTGGCAAGTGGCCAAATGACATGAAATATATTACC
TGTGACGATTACGACGGTAAGAATACGCCTAACAGGACCTTGGACCTTAAGACCGCGTTT
CAAGAGGTGAAAGAGCCACCGACATATGCCCCATTTTATATCCCCGAAGATGACCCTATG
AATGGAAGGAGTTTTATGGAGTTTAGCACGGATCTATCAGCGGTCACAGTCAAAGATGGA
GCTTCCATAGGCAGCCGTTCCCTAGGCTCATGGAAAGCTGGTCTCGCAAGTCCATTATCC
TTGACTGACCCTGAAAACATGAGCGATTATTCAGCGCAGCTCGTTTACAGAGTGGTCACA
ATCGAGCAACAACCGTTCATTATAAGAGACGACAGTGCTCCTAAAGGCTATAAAGGCTAT
TGCATCGATCTAATCGAAGAAATACGTCAGATAGTCAAGTTTGATTATGAAATATCGCTC
ACACCGGATGGTAACTTCGGTACGATGGACGAAAATGGTAACTGGAATGGTCTTATAAAG
GAGTTGATAGAAAAGAGGGCGGACATCGCTCTAGCATCTTTGTCGGTCATGGCTGAAAGG
GAGAACGTGGTAGATTTCACTGTGCCTTACTACGACTTAGTTGGTATAACAATTTTGATG
AAATTACCGAGAACGCCGACGTCGCTCTTCAAATTTCTGACCGTTTTGGAGAATGACGTA
TGGCTTTCCATATTAGCGGCGTACTTCTTTACAAGTTTTCTCATGTGGGTCTTCGACAAA
TGGAGTCCTTATAGCTATCAAAACAATCGAGAAAAATATAAAGATGATGAAGAGAAGAGG
GAATTTACTCTAAAAGAATGCCTCTGGTTTTGTATGACATCACTAACACCGCAAGGAGGT
GGGGAAGCACCAAAAAATCTCTCTGGACGTCTATTAGCAGCAACTTGGTGGCTATTTGGC
TTCATCATAATAGCTTCATACACTGCTAACCTAGCAGCTTTTCTCACTGTATCTCGTTTG
GATACCCCCATAGAGTCATTGGATGACCTCTCAAAGCAATACAAAATACAGTACGCACCG
TTAAATGGATCTGCGGCCATGACATACTTCGAGAGGATGGCACACATTGAGGTCAAGTTT
TATGAGATATGGAAAGACATGAGTCTGAACGACAGCCTCAGTGACGTGGAACGTGCTAAG
CTGGCCGTGTGGGATTATCCAGTGAGCGACAAGTATAGTAAGATGTGGCAGGCAATGAAA
GAGGCTGGTCTCCCAAATACAGTGGAAGAAGCAGTCCAAAGGGTGCGTGATTCCACAAGT
TCCAGCGAGGGTTTCGCCTGGCTGGCAGACGCTACTGATGTTAGATACCAAGTACTGACA
AGCTGTGATCTACAAATGGTTGGCGACGAGTTCTCCAGAAAGCCGTACGCCATCGCTGTG
CAACAAGGATCACCATTAAAAGATCAGTTTAATAATGCTATTCTACAACTCCTAAACAAA
CGTAAACTGGAGAAACTCAAAGAGACGTGGTGGAATAACAATCCAGCCGCAATGAAGTGC
GAGAAGCAAGACGATCAGTCCGATGGCATCTCCATACAGAACATTGGCGGAGTGTTCATA
GTCATATTCATGGGCATTGGTCTCGCGTGTATCACTCTCGGCGTCGAGTATTGGTGGTAC

AAATGGAGGAAACGACCCATCATCGGTGATGTTACTCAGGTTGATCCTGCGAAATCAACC
AGAAATAACGTCGATAACTTTGGCAATACAAAAACTGGAGAAGGCTTTACGTTCAGGGCT
AGAAACTTGGGCTTGTCTAACTTTAGATCCAAGTTTTAA

>EhipIR75q1
ATGAAAGTAATCGTTGTAATGTTTACAATTTTATTTGGTACAA
AATTAGTTACTTCAAGCTTAAATAAAAACAATGAATTGGCAATGATTGTTGATGTAATTA
AATCCTACGACAAACCCACTTTTGTAATAGCCAACGTTTGTTGGCCGACGTTGCAAAAAG
TTAAATTGGCGACTGAATTGAGTGACACGGAAACTCCAAAAATGGTTCAATTTATTAACA
ATAATATGATAACTGAAGATTTTAATCATAAGGAAAATGTATTTTTCGTCATCGATACTA
ATTGTTCTTTTATTGAAGACTTTTTTACCCAGGCCAATAATTCTAAAAAATTTAATGCCC
CATATAGATGGTTAATACTTGAAAAACCTTCGAATGAGAGTAGTGACGTACTTCTGAGAC
TTAATCATTTAAACATATTAACAGATTCTGAAGTTATTATATCACGAAACAGAAGCAATG
GTAGTTTTGTTTTTCATATGATTTATAAAATAAAATCTAAAAGCGAGTGGCGCTTAGAAT
TTTTTGGGACTTGGACAACTACTAATGGCCTTCGGAAATTAAATAATATGGTCACCGTAC
CGATCTCTATGAGACGAAAAAACTTATTGGGAGCGTCTATAGTTACTTCGCTAGTCATTA
CTAACAATAAAACCAAAGGAAATTTGTATGATACTCGTGATATTGAGGTCGACGGCGTGT
CCAAGACAAGTTATCGTCAGATTATGCCTCTATATTATTTCATGAACGCAACAAGAGTCT
TAACATTTCCAGACGCTTGGGGTTATTATATAAATGGAACTTGGAATGGTATGATTGGAG
ATGTGGTTAGTGGAAACGCTCATCTTGCAGGTTCAGTAATGTTCATAACAAGACAACGTA
TCGACTTCTTGGATTACTTAATACACCCTTCACCAGGACTAACAGTGAAATTTTTGTTCC
GAGAACCACCATTGTCCTATCAAAACAATTTATTCTTACTTCCTTTCAAACTCAACGTAT
GGTTGTGTATCGCTGCATTTGTGATAATATTGACACTCATCCTCTACGTAAATGCACTTT
GGGAAACCCAAAAAACTGAGATCATTTACACGAATAATTTGGATCACACCACATTAAGAC
CAAATGTGAGCGACATCGCCTTTTTGGTTATTAGTGCTATTTCTCAGCAAGGAAGTGCAA
TGGAACTGAAAGGAACCCTCGGACGCATTGTAACATTCATTTTGTTCCTCACATTTCTAT
TCTTGTACACGTCATACTCAGCCAGTATAGTCGCGTTACTGCAGTCCAGCTCCAAACAAA
TTCGAACGCTTTCGGATCTTCTACATTCAAAATTAGAACTCGGAGCAGAGGATACGCCTT
ACAACAGATATCACTTTTCGACCGCCAAAGAACCCGTAAGAAAAGCAATCTATCAAAAAA
TAGCACCTCCGGAGTCAAAGCCTAATTTTTTGAATTTAGAAGATGGAATTAAAAAATTAC
AAAAAGTATGTTTCATCTTACGTAATTTTATTGTGAATATAGAAAATATTATTAGATCTG
AAAATTTTACAATATAG

>EhipIR31a
ATGTCTATTGCGAATATATTACTGCTCATATTAGCGGGTGTA
CCTACCTATTGGACAACAATGCTCGTTCAAGCTATAGCAGATTTTTTCAAATATAAAATA
ATAAACACTGTAATTGTCTTGTCTTGTTGGTCTTCTAATGATCGTGTTAAATTTATGCGT
CAATTATCTGATCATGGATTAATCGCCACAATTTCTTGCGACCCAACTATACTCGATTAT
GTTCAGAACCATCATTTTCAAGGAATACTATATGTGAAGCAAGTAAATGATAGTTTGTTG
GAAAAGGTGAATCCGGTCTATTTTTCCAACTGGTATAAATGGCTCGTAATCAGTGACGAA
GCACCCAGCAGTCTACACGCGACTCGTTATGATGCTGATGTGGTTCTTATTGAGTCATCA
AAGAGAATTATGGCACGTGCAACAGGAAATAATGAAATAATGTCCACAGCGGCAATAAAG
CAAACTATTTACTTTAATGATGTCTACGTGCATCCTCGAAATGGAGCTTCTTTGAACCCG

TGGGCAGTTTGGACTGGAACTCTTGAAGTCACACATGAAAGGGAGAGAATTTTACGACGA
CTTGATTTGAAAAAGTATCCGTTGAGAATCGCAACTCCTGTGGGTCATTACTCTGAAGAC
ACTTATAATGGTACTTTTGAGGAATATCTAGCAGATAACACCATGCCCGAGCGTGATTCA
GCTACTCGCTGTGGGCATGCTGCTTCTTCACTAATTCTAGAATCTTTAAAAGCTACTGAG
GTTTTAACACCGACGCTGTTGTGGGCCACTGAATTGAATAACAGTAGTATGATGTTGAGG
GTGGCTTCTGGTACAGCTGAAATAAGTGGCTCCATCTTAAGAGTTTTGCCTGAGCGTATT
AAGCGCCTTGACTACGTTATGCCGATATGGCCTTTCAGCGTCGGCTTTACATATTTAGCC
GAAAGGGCGAGCAGTAGCAACATGTTCGTGGAGCCATTTTCACCCGGTGTTTGGTGGACA
TGTCTCGCTATTGCTGTTTTATTATCATTTGCTCAAAGACTCACAGCTAGAGAGCCGATG
GAGAAAGAAGGCGCTTACATCGCTGTATTGGCGACTTGGTTGCAACAAGATGCTAGCGCA
GTACCAGAAGGGGCATCAGGCCGGTGGACGTTCATCGTTCTCTCTGTATGTTCGATGCTA
GTACACGCTTACTACACCTCGGCCATCGTCTCAGCTCTCATGAGTACCGGCAGGAGTGGA
CCAGATTCGCTGAAAGCCCTTGGCGATTCCAAATACGCTATAGCTTCGGAAGACTACGAC
TACATGCGTTACACTATGTTTGGTATGGAGACCAACTGGGACGATTTGGAATACCTCAAG
AAAAAGAAAATGCATTCGAATTTCTATCAAAATATAGAACGTGGAGTGGAACTCATACGA
GAAGGGAATACCGCTTTTCACACAGAGTACAATCACATTTACCCGCATCTGAGGACATTC
AATGATGAACACTTATGCAAACTTGCTTACGTTGATACTATTCCAGAGATAATGACATGG
ATAACAACAACTAAACGCTGCCAATGGACAGACGTGCTGCGTACTGCTGGCGGATGGCTA
AATGAAGTAGGATTAGTGAAACGTTTGGTATCTCGCTGGCGAATACGTCCACCGCCATGC
CGAGCTTCATTACTTGCAGAAAGAGTTAAATTCGGAGACGTAGCTCCAGTATTATGTCTA
ACTGCTATAGGTGCCATAGCATCATTGATACTATTAGGACTAGAAATAATATTTGCTAAA
TGGACAGGAAGTAAATATAGAAATTCTCCCGTCAGTGATGTCGCTGATGTTGCAAGTGGA
GACGAAAATATTAATAAATAA

>EhipIR76b
ATGGAGCTTATCA
TTTCATCAATTTGCAATGCCACGTTTTGCGAGGCCGTATATGACAATCCATTAATAGAAT
CGCAATTAACGAAGACACAAATCGAATTATTGGCTTTGGCCGAGGAACTAAACGGCAAAC
ATTTAAAAATCGGCACATACAACAATTATCCGCTAAGCTGGACAGAAAGAGCAGACAACG
GAACGTTAATAGGTGGTGGGGTTGCGTTCACTATTATAGATATTTTGCGTGAAAAATTCA
ATTTCACATTTGAAGTTGTTATCCCTAATAACAATTTTGAATTTGGCGGGTCCAGACCAG
AAGATTCTTTGATAGGTCTTGTTAACAGCAGTAAAGTGGATATGGTGGCTGCGTTTATGC
CGACATTGTATAAGTTGAAAGATCTAGTTTCGTCTTCGGTTGACATCGACGAGGGAGTAT
GGGTAATGATGCTGAAGAGGCCCGCCGAGTCCGCCGCAGGATCAGGCCTGCTCGCGCCGT
TTGAAAGCCACGTGTGGTATTTAATTCTAACAGCGGTGTTGTCTTACGGTCCGTGCATCA
CATTACTGACGCGCCTGCGCTCGAAGCTGATCAAAGATCACGAGCGCTACATCCCGTTGT
CGCCGAGCTGTTGGTTCGTTTATGGAGCCTTTATCAAGCAGGGGACTACTCTGTCTCCAG
AGGCAAACACAACCCGAGTTCTCTTCGCAACTTGGTGGCTGTTCATAATATTACTGTCTG
CTTTCTACACCGCTAACTTAACCGCATTTCTTACCCTCTCAAAATTTACATTAGCCATAG
AAACGCCAAGGGATTTGTATAAGAAAAACTATCGATGGGTCGCCACGCAAGGCGGAACTA
TAGAATATGCTGTTAGAGATCCAGATGAAGATATTCATTATCTAAATCAAATGATATCAA
ACGGTCGAGCAGAATTTCGTTCAGTGTCCAACAGCGAAGACTATCTGACCATGGTGAAAG
GAGGCGCAGTTCTTGTAAAAGATCGGACCGGCATAGACCACATGATGTATGCAGATTATT
TGAACAAAGCCAGGCAGGGGATCGAAGAAACTAGTAGATGTACTTATGTCATCGCTCCGA

ATTCCTTCATGAAGAAAAATCGAGCTTTCATATTTCCAAAAAATAGCAAATTGAAAAAGC
TTTTCGATCCAATCCTCACGAATCTATTGCAAGCAGGGATAGTGAATTTCCTTAAAAATC
GGGATTTGCCGAGCACCAAAATTTGCCCTCTGGACCTGCAGTCAAAAGACCGACGTCTCC
GGAACAGCGATCTTATGATGACCTATTTGATAATGGTAATCGGTCTGGCAGCTGCCATAG
CAGTGTTTATAATGGAGATTATAATAAAAAAATGTTTCCATATACGACTAAAGACGGAAG
GACCAAGACCGAAACGGACAAGGGCGAATAGAGTTAGATTCCAAAATCACGATGAAACCC
AACCGCCGCCGTACGAATCACTTTTCGGTCGTAACTCGAGGTACAAGATGACAGATCAAT
TCCAAACGAAGATAATTAACGGCAGAGAATATTGGGTCGTGGACACGGTGAGCGGGGACA
CCAGGCTGATACCAATGAGGACGCCGTCTGCTTTTCTATATCAACGTCAAAGGCGTATGT
AG

>EhipIR75p1
ATGGTGGGCAGAACGGCAGTCCGTTTTGTGTACCGCGAGC
CGCCTCTCGCTTACATCTCCAACATCTACGCGTTGCCGTTCAGCTCCGCCGTCTGGTGGG
CCATCAGCATCTGCGTACTTTGTTGCGCCTTGTTCCTTTACATCACGTCCAAATGGAAGC
TAAGTTTGAGAATGTATGTATGTTTGGGATAG

>EhipIR21a
ATGGAACTGTTATTAAATATATTAATTTTG
AAATTCCTTTTTTATGCACACGGTCAAGAGATTGAATATTACCCCTCGCAGAATGTGTTA
GATAATTCCGTTGTGAAAATTGGTAGTAATTCCAAACAACAGCTAATAAATACCGAGTAC
AATGAATTATTTTCTAAAAATGCTCACGATAAGATACAATGGAGATACTTTAACGAAAAC
GAATCGGATAAAATAAAAAATATATCTAAAAGGGCAGCAGATCCGGTATTTCATGGACAT
CCGAAAACTACAGAAGAATTATGGAACGAACATTTTCTAAACCAAAGCTCAGCTTTCGAT
CAAATGCCGTCACTTATAAAATTAATTCATAATATAACATTAACATATTTAAGTGATTGT
ATTCCTGTAATACTTTATGACAAACAAGTAAAATCACAAGAAAGTTACTTATTTCAAGAT
CTTTTCAAAGATTTTCCAGTTACCTATGTACATGGCTACATAAATGATGATGATACATTA
AAAGAACCAAAATTATTATTCTCGGATCAAAACTGCTTACATTTTATAGTGTTCTTAACA
GATGTGAAAACATGCACAAAAGTGTTAGGGAAGCAATCTCAAAGTAAAGTAGTAGTAGTT
GCGCGGTCTTCACAATGGGCAGTACAAGAATTCCTTGCTAGTCCACTCTCTAGAGTGTTT
GTTAATCTGCTGGTAATTGGACAAAGTTTTAAGGATGACGACGATGATAGCTTAGAAGTT
GCGTATATTTTGTATACACATAAATTGTATACCGACGGCTTGGGTGCTAGTCGGCCTGTA
GTTCTTAATTCTTGGTCTCACGGCAAGTATTCAAGAAAAGTTAATTTATTTCCAAAAAAA
ATGAGAAAAGGTTATGCAGGTCATAGGTTTTTAGTTGCAGCTGCTAATCAACCGCCGTTT
GTATTTAGAAGAATAAAAAGAGATGAAGAAAGTGGCAATCTGAAAGTCGTATGGGATGGC
ATAGAAGTTAGGCTGCTACAATTATTGGCAGACAGAAATAATTTCTCAATTGAAATAGTT
GAGCCCAGAGAACTAAATTTAGGCCCAGGAGATGCAGTGGCTAAAGAAATAGCAACAGGC
AGAGCAGACATCGGAATAGCAGGAATATATTTGACTCAAGATAGAATTCGTGAAATGGAT
GTGACTTTCGCGCATTCACAAGATTGTGCAGCATTTATTACAGTGATGTCTATTGCATTA
CCTCGATATCGAGCCATTCTTGGCCCATTTCATTGGCACGTTTGGTTAGCTTTAAGCTTC
ACCTATATCTTCGCTATATTCCCTTTAGCTTTTTCGGATAAGCTTACATTACGTCATTTG
ATGCATAATGGCGGAGAAGTAGAAAACATGTTTTGGTATGTGTTTGGAACATTTACCAAC

TGTTTTACTTTTGTTGGTAAAAATTCTTGGAGCAAAACAACAAAGATTACTACTAGACTG
TTGATCGGTTGGTATTGGGTGTTTACAATAATAATCACGAGCTGTTATACTGGTTCTATC
ATAGCTTTTGTGACCTTACCAGTGTACCCTGAAACAGTGGACTCTGTCAGACAGTTGCTA
TCAGGATTTTATAGAATTGGAACTTTAGATCGCGGTGGTTGGGAAAGGTGGTTTTTGAAT
TCATCGGATAAATACACGAATAAGCTTTTTAAGAAAATTGAATTCGTGCCTAGTGTTGAG
GCTGGTATTAGAAACACAACCAAGGCTTTCTTTTGGCCTTATGCCTTTTTGGGTTCACGT
GCTGAGCTTGAATATATTGTTCAATCGAATTTTTCTATGACTAAATCGAAACGAGGCCTA
CTGCATATTTCTAATGAATGTTTTGTACCTTTCGGAGTATCATTTGCTTTTCCAAATAAT
TCATTATACACAGCTAAATTCAGTAATGATGTAAGAAGAATGTTACAAAGCGGAATTATA
CAGAAAATTGTTGATGAAGTGCGGTGGGAAATGCAACGTAGCAGTACTGGAAAACTGTTA
TCGGCCGGTATAGGATCTTTAAACACATTATCGATCGAAGAGAAAGGACTAACTTTAGAA
GACACACAAGGCATGTTTCTTCTTTTAGGTGCTGGATTTCTTATAGCGGCATCAGCGCTT
ATATCAGAATGGATAGGCGGTTGTTCAAGACTGTGCCGTTTGAATAGGAACAAAAACCCA
CCCACCAGTGTCAATTCTGGAGATCATCTGATACCAACTCCTAAAACTGATACACAGAGT
ACGATAAACATAATTTCTGATGGCGCTGATAGTAGATTACATTTTGATACAAGACCGCCA
AGCGCCGATTCAAGAGATACTTTAGACGGTCAAATAATAAACGTTACTGAAGAAAATATC
ACAGTGCATGATAATTCCAAACTTGATGGATGGGATTCAAGAAGATCCAGTTCTATAGAT
TTAGACAGAGAAGTAAAAGAGATATTTGAGAAAGATCAAAAGAGAAGAAGGATCCTTTCA
GATGGTATGATCGAATTATCTGGTAATAAAAGGCATCCAACTGCGTCCAAAGGAGCATTT
GGAGATACTGTAGGAAATTAG

>EhipIR75p2
ATGTCGATGCTATCATTCTTATTGTTGCCAATTCCATGTATCAT
TTCCAGGGAACTTCAATATGATGCTTTCACTAAAATATGTTGGATAAATATAAAATTAGC
TTTTCAAATGTTGAATGCAACTCCGCGCTACATATTCAGTCATAGATGGGGATACAGGAG
GAACGGTCAGTGGTCGGGAATGGTGGATGACTTGCTCTCAGGAAGAGCAGATGTGGGTAC
GAACCTCTTAATGACCGTAGACCGTTTGGATGTAATTACTTATACTGAAGGTTTATCTCC
GTATAGGGTGCGTTTTATATTCCGCCAACCCCCTCTATCTTACGTCGCTAATATATTTTC
CCTGCCATTCTCGAGTGGAGTCTGGATAGCAACAGTCGTATGCGCAATTGTCTCGACTAT
TGCTTTATATTTGGCCAGCAAATGGGAAGTTGCTATTGGAAAGAGTCCAACACACTTGGA
TGGGTTTGGAGATTCCTTGTTTCTAACAATGAGTGCAGTAAGTCAACAAGGATGTATTAT
GGAACCTAAAAAGATTTCTGGTCGCATAATAATGTTGTTCGTGTTTGTCTCCCTCATGGC
TCTATACACGGCTTATTCTGCCAACATCGTGGTCTTGCTACAAGCCCCGTCGAATTCTAT
TAGAACATTGTCTCAACTGGCACGTTCCAAAGTCACTATCGCCGCTAATGATGTAGATTA
TAATCATATTGTGTTTAAACTTTTCAAAGATCCAGTTAGAGTCAGTATTCAAAAAAAACT
CGAACCTGAAAATGGAAAAGCACAGTTCTATGACATGAATGAAGGCGTTGAGAGAATCAG
ACAGGGCCTATTTGCATTTCATTCCATAGTTGAACCGGTGTATCGTCGTATAGAAAAGAC
GTTCTTGGAAACTGAGAAGTGTGACCTCACCGAAGTGGATTTTCTAAACAGTTTAGATCC
ATTTACGCCTATAAAGAAACATTCACCGTATACGGAATTATTGCGAGTTGTCATCAAACA
AATTCGCGAATCCGGTATATTATCGGCTGTCTACAAACGACTTCAAGTACCTAAGCCTCG
ATGCACCGAGAAAGTATCAGCATTTAGCAGCGTTGGTTTGTTGGATCTGAGAGCCGTTAT
GTTCCTCATGCTTATCGGGGCTGCTGTCTCCATAGGCGTTATGTTTATTGAAATAATATT
TCATAAATTAAATAAGCGGCAACGGGGAATATGA

>EhipIR68a
TTGCATTATTTGGACATGTTAAAATTAAAATTAGCCATTTTTATGTTTCTGA
GCATAGAAGTTAGTACCGATATTGCGCCTATTTTGAAACAACTCCAAGAACGCAGAGACC
TGGAGTATGTGGTCACCGACTTGGTCAATGTGGTGACACGGTATGATGACGTCACGTGTA
TCGCTATTATTTGTGACCAAGTCTATTTGAACGTGTTTGAGGGGACATTGTTCAGGAGAA
CTCTTGCTGTGCCTTATGTTATGATTGTAGTTGAAGATTATGAAGATTTACTATCGCCCA
ATTTCGATACTTTGCAGTCTTTACGAGAGACAAGGAAAGTCGGTTGCAATATTTATATCA
TATTACTTGCTAATGGTATTCAAGCTAGCAGACTCCTGAGATTTGGTGACAGATACCGCA
TTTTAGATACCAGAGCAAAATATATTATGCTACATGACTTCAGACTGTTCCAAAGCGATC
TTAATTACCTCTGGAAAAGGATTGTCAATGTAATATTTTTACGTTATCACAAAAAAATTG
TGGGAGTTTTAAAGAGTAAAGCGTGGTTCGACCTATCCACCGTTCCTTTCCCGAACCCAA
TCAAAGGGGTCTTTGTTTCACGAAGAGTTGACATATGGAAGAATGGGAAATTTCATTATA
ATAGAGCCTTATTTGCTGATAAAACGAGGAACTTAAATAAGGAAGTTTTGAATGTGGTAT
ATTTGGATTACGTTCCGTCGGTTGTGGTGATAAAGGATAATGATACTAGTAAAATTGGTG
GTGTGGAGATTGAGATACTGAATATGATAGCCGAAAAAATGAATTTCAAACCCAAATTAT
ACCAGCCCATGAACGTAGAACTTCACAAGTGGGGTCAAAAGCAGCCCAACGGTTCATTTT
CTGGTCTTCTAGGGGAAATGGTGAATGGTAATGCCGACGTGGCGTTAGGGAATCTACAAT
ATACTCCGTATCATTTGGATCTGACAGATTTGAGCGTCCCTTATACGTCGCAATGTTGGA
CGTTTTTGACACCAGAATCTCTGACTGATAACTCTTGGAAAACTTTAATACTGCCGTTCA
AATTATACATGTGGATAGCAGTTTTATTGGTGCTTCTGGTGACAGGAATGATATTCTATG
GCTTGGCGAGGTATTATATAAACTTAATGGCATATAAACATGACCCATCGGACATTGGCG
TCAGCGTACCTTTAAGTTCGAAGGGTAAACACGAGGAAGTCGACCAATTTGACGAGAAAC
CTGTCGGCCTGTACTTGTTTGGTGAAATAATTAACAGCATCCTTTACACGTATGGCATGT
TACTGGTCGTGTCCTTACCTAAATTGCCTACGGGATGGTCCATCAGACTTCTCACCGGAT
GGTATTGGCTGTATTGCATTCTTCTAGTTGTCTCGTACAAAGCTAGTATGACCGCCATCT
TAACGAACCCTGCACCGAGAGTAACAATTGACACTTTAACAGAATTAGTAGAAAGCAAAG
TCACATGCGGCGGATGGGGAAGCGAAACTAAGAAGTTTTTCGAAAACTCTCTAGACGATG
CAGGACAGAAGATTGGTCAAAGATTCCAAACTGTCGACGACCCTAACGAAGCCGCCAAGA
GAGTAGCACAAGGCCATTACGCTTATTACGATAATAAATATTTTCTAAAATATTTGAGCG
TGAAAAGGAGAAATGTATTCATGAATATGGAAATAGAAATGGATAATTCAACTATGAATG
GCACAGAAGTCACTGTTAGAACTGAAACCGAAAGAAATTTGCATATAATGACAGATTGTG
TCGTAAATATTCCCGTTTCTATAGGTTTTCATAAGAATTCGCCATTGAAACCCTTAGCAG
ATATTTATATTAGGAGAATAGTAGAAGTAGGTTTAGTAGAAAAATGGCTCAATGATGCCA
TGCACCCAATAAAATCTTTAGAAGCAGAAGATGAAGAAATTAAAGCTTTGATGAATTTAA
AAAAGCTATATGGTGCTTTTGTTGCTTTAGCTATAGGCTATTTTTTGAGTGCAGTTTGCT
TGATCGGTGAATTAATACATTGGTATTTGATTGTTAAAAAAGATCCACATTTCGACAAGT
ACGCTTTG

>EhipGR1
ATGTCCATGTCGATGGTAA
CCACTACTGTTTACCACATCGTGTTACTTTACCAATATATTACTGTAATTATAAAGCAGA

TCGATAATAGAAAATACTTCAACACAAATAATATGGTATTTTTTCTGACAAACTACTTTG
TCAGTTCAGTTTTGATACTGGTGGCTGCCCAAAAAGTTTATAATGAAAGTGTAAAATTGA
AAATGTCTCTAGCAAGATTGAACAATACATTGATGGCATGGCCGGAAGAAACAGAGATAC
TGCAAGCTATCAGAGATTTTCAACGAATAGTTAATACAGAACCGATTATAATAACACTGC
TGACTGCTATGCCGTTGAAAATGCCGCTGCTACTCAGTATGGTTTCTATAGCGACCACTT
ATGCTATTATAGCGTTACAGTTCAATCATGTAGTATGA

>EhipGR2
ATGGATTCTGTTTTTCTTAATACAATTGACTGCTTT
CTCAGTAACTTGCGCTTGGTTTGTAAAGCAAGCGAAAGATACAAAATTACTATGTATTCA
TGTTCTATCAATGTATTTCGAAGGCCCTGTGAGAAAAGCAGCGAAGGAAATGATTCGATT
CATCGAATTTCAGAAGCCTGCATTTTCTGTATACGGAATATTCGTAGTGGACAAACGATT
GCCTTTTACACTAATAAGTCTTGTCACAAGCAACGTCTTAGTTTTAATGCAAGTCACTCT
CTAATTTAA

>EhipGR3
ATGTATAATCCTTCAGGGTTGACAGAAT
TGAATAATCAACATTTAAGATCTAAATGTAAAATATCCACCGCATTGATAGTCTATTTTG
TGGTTGCGGACATTTGCTTTGGTTTAAATTTTGGTTTTACAAAATTGTTGAATAAAACGT
TAAGATCTATAGTTAGAGTGCTATCATGGATTTCGTCTTTGGCCATGGTTGTTATTGTGA
GTGCACCTTTGACTAGTTTTAATAAGCAGGCAGCTTATTTATCTTTGTTCACATTTCAAT
ATATATTATCGCTTCTTATATTATTACTATCGAAATATACTGTATGTGACTTTTTAGTTG
ACATAATTGAAGTGAACAAACTTGTGGATTTTGACAAATTTCATGTTGGTGTAAAGATTA
TGGCATATTCTTTGACTACTTTTGTGGTTAAACTTACTGTTTTATCAGTATTCTGTGGTT
ATGTGCTACAGTTCTGTGTTCATTCCATTTTTCCTGATTACATAACTTATATACCACTGT
TAGCACTTGACGTGGTGTCTGTTGTGCACACTTTAGTATTTTATTATATCTATTGTCACG
TCAAATTTCTGAAAGATGCTTTGAAAAATCGCGAAATAGATCTGGATGAAGTGAAGCAAA
TATATAAAGAGACGGGTGACTGTTTTGATAGAATTAAGCCTTCTTTCGACAAATTGTTTA
TTATGGGAGTAATATTAGGCATACCTAAATTAATGGGAGTAATATGGGAGCTCCTACTTT
ACATAAAAGAAAATGCTTCGTGGGTGACATACATGAGCCACAACGTGTTTGCATTGCATT
TCGTTATCCAAATGTGCGCACCCGCAGTCGTAGCAGAAATGGTATTGGCCGAAACGGACT
CCATCAAATTGATACTCAGCAATCGACTATTACTTAGTGGTGACGTAAAAGAAAACAGAA
AGGTGAACAGTCTCCTTGGGTACATGGAGACTCGTCCGCTCCATTACAAGGTCTGGCGCA
TAGTGCACGTCGATCTACATCTCCCAGTTTCACTGCTCAGCATATGCACCACTTATCTCA
TAGTTATCATACAGTTCACGCATCTGTACAATTAA

>EhipGR4
ATGACTCCTATTCTCTGTCTTATAGATGAAATGAA
GAGAATGTGCAAAAACATAAGGAGATTGAACCGAGCCACGTTCGTGAAAATGGCGCCGTG
TGGAATGTTCACAGTTGATGCAACGTTACCTTTGAATTTGGGTAAACTGATAGCTACTTA
TATTGTCGTGCTATTGCAGTTTGCTTTCCTTGGATAG

>EhipGR5
ATGACAGACGCTATCATGACTCAAATGAATTATATC
GTTGAACTTGTTCTTCTGCTCATCTTTTGTACCGTCGCTTATATCAGCACCTTCTTAAAC
CGTCATAGATACGTTAGAATCTTGAACGAAGTAATCTCATCCTGGATTGAATTACCGAAC
AGCAGCACCGATATTATTCTTGGACGCCTTCGTTATCAAGTGAACGTCATCGCAATGAGA
ACACTTCTTATACTTTTTGTTTTACAGATTTGCGTTAATTATACACGCAGTACTAGCATA
TGGAAAATGATATTGGTTTCAATCACTTTTAATTTA

>EhipGR6
ATGCGACTCGAGCTGCC
GCCTCGCCGGGCGCCTACAAATGTAAGCACGACGGGTTGGTTAATGGTCATCCCCGTGCT
TTTGATAACAGCAATTTTCGGAATTCTGTGTGGAATGACATTTACTGCACAGCGAGTGCA
GAATAACGCAGATGTGCTTAAAGAGCGCGTTACTAAAATTCTCCTAGATTTACACACAGA
TGACGTGTGTTATAAAGCCATAAAAGGATTCCTGAAAATCGTAGCTGCTCACCCGATCCG
CACCCGCGCATTCGGTGGAGTGTTATTTAACAATACTCTAATACCGACTTGCATCGGCTA
CATACTTACCTACACAGTGATCGCGTTACAGTTTGATAATGTAATCTGA

>EhipGR7
ATGCGTTTGTTCGCGTGCGTCGCCGTTCTCGTCAGCTTGGTGTACCACTGC
GAACAAGCCTACAGGCAGTCGGACAGAATTATTTGGATGATTGACCATTTATTGATAAAC
AAAAACCCTAGCGATGCCCTCAGGTCCGCTCTGGGCGAACTGCGCGCGCTCATACAATCC
CGACCTGTTCGCTTCCACATGGCGTATTTCTTTTGCCTCAATTATTCCCTACTGGTCTCT
ATCGCTTCCGTAGTAGTTACATATACAATCATATTATTACAAAACATGAACTAA

>EhipGR8
ATGTTATTGATTTGTTCAAGTCTATTAATAGAAATGATTACGTTTATGAACGTATGTATA
AGATTTGTCGTAGGAACAATGTTTACACTGTTCGAATCGAATCTTTTCCCTGCTGTGACG
TTTTTCATGCGGTTCTGTTCATGTATTGTGTTGTTGGGAGCTGTAGTCTATCGCTGCGAA
CAAAGTTACTGCCAGAGTGATAGGATTGTCTGCATATTGAATCAGATGTTGTTTACCAGG
AAAATTAATAAGGAGATGAGAACTCCGTTGAAAGAACTGCACATACTCGTAACTTCCAGA
CCGATTCGATTCTACGGTGCTAATTACTTTCTAATCGATTACAAACTATTGGTTTCTACT
GCATCTTTTGTCGTCACTTTCACAATAATATTGTTAGACAATCTTTAA

>EhipGR9
CTGCGAGTAATAGCAGACTATATAAAAAATTATTATCTTGTGACAGAA
TGTCTGCCAGGAAGTACTGTGGACTCGATTATTAACAAAAATTGGTTCCACTCAGATACA
TGTATTCGGCCTTTGGAATCTTTACCAAAAAGAAATCCATTAGAAACCTTTTCCAGCATC
GATAATAATCACCACGAACTTAAATGGCTCAGTCGTTGTTACTTGCTTTTGCTTGAGCAA
TGTATTTTTATCAACAGTATGTACGGAATGAGGATTTTACTAAACAGCTTGAGCTTACTT
ATCGATATGGTAAGGTTTACCAACATAGCAATCAGAATGATGATTGGATCACAGCACACT
ACATACGATTCGGGC

>EhipGR10
ATGTTATTGCGCGTGAAAAAATATTTTTCCTCAATTCCAAATCGGGACGAGGACTTAA
ATTTTCGCGGAATATTCAAACCATTATATATTGTGCTGTCAGTTCTCGGTTTATTTCCTT
ACAGTGTTAAATTTTACAGCAAAAAACAACATTTCAGAATAATAGATAAATCTATTTACA
TAAATTCTCTATGTGGCGTTACCATTGTCTTAATTATGTACACGTTTCTAGTGTTTCATA
TTGAATATCTTTTCCAGTCTAGCGAAGGC

>EhipGR11
ATGTATAAGTCCTTGGTATTATTATGTGTTGTGTTT
TGTATAGTGTGTGAGGCTCGGCCAAAATGGCAGTTCTTACCTCCCGTGCCAGGATACGTA
CCTGTGTACATAAGAAAGGGCGACACACCCCTCGAAGAAATCAACCCTGACCTAGCCGAG
GCTTTCCACGCGTTGCCCGCCGGACGCAGCGCCGGCAAACAGCTGAACGCATCGCCAGAA
TTTCCAGAACAAGCTGACCAACCACAGCCAGATGTTCCAGAACTTCCGATCGACCGACGT
CCTTATGAAAAGAAACTGCTAGAGAAAAAATCTAAAAAAGCGAGCTCGGAGCTTACAAAA
CCTATAGAACGGAGTTAG

>EhipGR12
TTGCTACTACTTTTTAATTTGTATACGAAAATCAGTCACCCTCAAGTTCT
GAAGGTAAACGTTTCTGTTAGAGTCACGGATTCATTGAAAAACATTTTTGAGTATTTTCA
ATATGTCGTTGACTTATATTTGGTTTATAAAGGTAAAAACGCTTGCTTGGAGTATTTAAA
ATTATACAAATCGATAGATAAAATAATCCGTATGCGTTACTATAGCGAAATTAGGTATAG
GGTGGTGAAAAATATTCTGCTCGTAGTTATAGTATGGTTAGTTAGTTCAATTTGTGATTA
CATAGCAATTGTTCAGGCATACGGATGGTTTCTCCCCACAGTACATAGTTTAGATTATCT
GAATTTCTTCGTGAAATGTTTGAGTGTGTTGGACATCATATCTCAAGTGGTTCAGGTTGA
ATACCGTTTAAAGATGATTGGAGATTTGCTTCTGGATTATTCTTCATCACTCGACGATGT
AATAAGCGATGTAAATTACCACCACTCTTGCGATATTAGTAGAAATACGCAAATGGATTC
ATTGCAGACATCGAATTATGAA

>EhipGR13
ATGCCAGATTTTAAAAGCGTCGATGACTTCGTTAGAACTA
TATCTGAACTTCTGGCATATTTAAGTTGGTCCGGTATAGCATGGAACCGAAGTATTCTCC
AGCAGTTATGGTCTATATTTCTGATTGGATCGTTTATTATTTATTCAATGTGTTGTGTGA
GTAAATTGACAAACCTTCGAAATTTTCATCAAACGACTTCATTGATAGGACATTTTATCA
TATACTTCACGTTGTGTATTACTCTTCTGGGCTTTCGGAGATTGAGAAATCTTTGGCCAT
CCTTATCTGCTCTATGGACGACTACGGAATACAATATGGCCAACTTAATTAAACCAGATA

AGTTGTTAAGAAAAAGAATGCTCTGCGTGACTGCCGTGTCTTTGTTTATAACTATTTTGG
AAAATATATTACGTTTTAGTGTTGGATCTGATTTCATAAACAAAACTTTCTTAGAAGCTT
TCCAACAATATGCAATCCATATGAATACATCAATATTGAACGCTAATAATTATACGATTT
TCAAAGGCATGTTCTTAAGCGTGATAACGTTAATATCATCGATTCTATGGAACTTACGTG
AAATGGTGCTAATATTGTTTAGTATCGGTCTAACGTCTAAACAGAGACGGGTGAATAAAA
TCATACAAAATTTCTGCATGGGAGACACATTTATGGGATCTGCAAACCTTCGGAGATCGC
GTCGCATTCGTGAGAATTATGCCAATTATATGACATTGGTTCGAGCCGTGAACAACGCTA
TAGGGCCATTAATTGCTATGTCGTGTGTTGGAAATATGTTTTTCATCGTCAGAAGTATAT
ATATGGGCATCGACACACGGACTTACAATTTTGTACCGGCAAACGAGAGACTGTTACAGA
CATTTTCACTAGTTTTTTTATGCGTTCGTACATTTATCGTATTATTGACAGCTTCGAACT
TGAATTGTCAATCGCAAGCGGCACTCCAATATTTGAAAAATGTCGACACGAGAAATTATA
ACTTGGAGATTCGTCGCCTGGAGTTTCAAATTCGTAAAGATGCGATGGGACTTAATGTCA
TGGGATACTTTACCCTTAATAGGACATTTATATTGAAAGCTATATCGACGATCATAACCT
ATGAATTGATTTTAGCTCAATATGAGAACCGTATGAGGGAACAGTATGATTACCATAATA
ATAAATAA
>EhipSNMP1
ATGGA
CAAGTATTCCAACAGCTGTCTCCTCATACGGACCGCTGCAGTCAGATACTATTCGCTGAA
GATGAAATTGCCTAAGCATATGAAAATCGCCATGGGTGCCGGAGGAGCGGCCGTTTTTGG
CGTATTGTTCGGTTGGGTTATATTCCCAGTTGTACTCAAAAGCCAACTCAAAAAGGAAAT
GGCATTATCACAAAAGACAGACGTAAGACAAATGTGGCAGAAGATACCATTCGCTTTGGA
CTTTAAAATTTATTTATTCAATTACACAAATCCGGAGGAGGTCCAGAAAGGCGGAATTCC
AATTGTAAAGGAAGTTGGACCTTATCATTTCGATGAATGGAAAGAGAAAGTGGAAGTAGA
AGATCACGAAGAAGATGACACCATCACGTATAAAAAGCTGGACGTATTCTACTTTAGACC
AGACCTTTCGGGCCCTGGACTAACTGGCGAGGAGATAATTGTTATGCCTCACGCTTTCCT
TCTGAGCGTGGTAACTGTCGTCTCGCGTGACAAGCCAAGTATGTTGAACATGATAGGCAA
AGCCATCAATGGTATATTCGATAACCCTCAAGACGTGTTCATGAGGGTCAAAGCGATGGA
CATACTTTTTAGAGGAGTGATCATCAATTGTGCAAGAACTGAGTTCGCTCCAAAAGCGCT
TTGCACGGCACTGAAAAAGGAAGCGGTCTCAGGATTGGTAATTGAACCAGACAATATGTA
CAAGTTCTCCATTTTTGGTACGCGCAATGGAACAGTAGACCCACATGTGGTAACCGTTAA
ACGTGGGGTGAAGAACGTGATGGAAGTGGGACAAGTGGTGGCAATCGATGGTAAAACCCA
GCAGGACAAATGGAAAGGCTCTTGCAATGAATATGAAGGTACTGATGGTACCATTTTCCC
GCCTTTTCTGACCGAATCCGATCGTCTGCAGTCGTTCTCCAGTGATCTATGCAGGTCATT
CAAACCCTGGTATCAAAAGAAAACATCCTACAGAGGAATAAAAACGAACCGATACGTTGC
CAACATTGGTGATTTTGCAAACGATCCAGAACTACAATGCTATTGTGAATCTCCAAGTCA
ATGTCCTAAGAAAGGACTGATGGACTTGACAAAATGCATAAGTGCGCCAATGTACGTCTC
CATGCCACATTATTTGGAGAGTGATCCTGAATTATTGCAAAATGTGAAAGGTTTAACTCC
TGACATCAACGCGCATGGCATCCAAATTGATTTTGAACCTATAACAGGCACTCCGTTGGT
CGCGAAGCAGAGAATTCAGTTTAATTTGCAACTTTTGAAGAATGACAAATTGGACCTTTT
TAAAGATCTGCCTGACACGATAGCGCCTCTATTTTGGATTGAAGAAGGTTTGGCGCTCAA
CAAAACATTCGTGAACATGATGAAACATCAGCTTTTCATTCCGAAAAGAGTAGTCGGTGT
TGTTCGTTGGTTGCTGCTGTCTTTTGGCATACTTGGCGTCTTAGGAAGCGTCGTCTTCCA
CTTCAAAGGTAGAGGTGTCCAGACGACCGCATAA

>EhipSNMP2
ATGATTGGAAAGCATTCGAGATTGTTTTTCGGGATATCTCTGGTGGTGCTC
GTGGTGGCTATCATTTTAGCCGCGTGGGGATTTCCTAAGATCATCCAAAATCAAATTCAT
AAGAATATACAACTGGACAACTCCTCGGCAATGTTCGAGAAGTGGCGGAAAATGCCGATA
CCTTTCGATTTCAAGATATACGTCTTCAACGTGACGAATGCCGAAGACGTCAACAACGGT
GCCAAGCCCAAATTAGCCGAAATAGGACCTTATGTTTATAAAGAATACAGAGAAAAAACA
ATATTAGGATATGGACCAAACGATACAATTCAATATACCGTGAAAAAGAGATTTGTGTTT
GATAAGAATGCCTCAGCGGAACTGTCTGAAGATGATGAGGTGACAGTCATCAATTTTTCT
TATATGGCTGCGATACTGTCGGTATACGAGCTGATGCCCAGCATGATAGGCATGATCAAC
AAAGCGCTGGAACAATTTTTCCCCAACCTTACTGATCCTTTCTTGCGTGTCAAAGTTAAA
GATTTATTCTTTGACGGAATTTATCTGAACTGCGTAGGAGATAATTCTGCTTTGGGACTC
GTGTGTGGTAAAATCAAAGCGGATATGCCACCGACTATGCGTCCTTCTGAGGACGGAAAT
GGTTTTTACTTCTCAATGTTCTCTCACTTAAACACAACAGAAATTGGTCCATACGAAATG
GTGCGAGGTAGAGTTAACATGTACGATTTGGGACACGTTGTCTCCTACAAGAGTAAAACG
TCTATGAGCCAGTGGGGTGACCCCTACTGCGGTCAAATCAACGGATCAGACTCAACTATA
TTCCCACCGATTGATGAAAACAACGTGCCGAGAAGACTGTACACATTCGAACCGGACATT
TGCAGATCGATGTACGTCGGTTTAGTGGGCAAAAGAAGTTTGTTCAACTTAACGACATAT
TATTACGAAATGACTGACGCCTTGGCAGCGAAAAGCGCTAATCCTGACAATAAATGCTTC
TGTAAAAGGAATTGGAGTGGCAATCACGACGGTTGCTTGCTTATGGGTGTGCTGAACTTA
ATGCCGTGCCAGGGCGCGCCAGCCATCGCCTCCATGCCACATTTCTACCTCGCTTCGGAG
GAACTCTTGGAGTACTTCGACGAGGGAATTCAACCGGACAAGGAGAAACACAATTCTTAC
GTCTATATTGACCCGGTGACGGGCGTAGTGTTGAAAGGTTTGAAACGACTTCAGTTCAAT
ATTGAACTACGAAATGTCAAAGATGCGCCACAATTGGAGAATGTACCCACTGGACTTTTC
CCGCTATTGTGGATTGAAGAGGGCGCAGAAATACCAGAATCCATACAAAATGAACTTCGC
CAATCACATTCAATGTTGGGCTACGTGGAGACCGCCCGCTGGATGCTGTTAGTCATCGGT
ATAGCGCTGACAATCATCAGCGCTGTTATATTGGCGCGCTCATCTTCGATCCTTGGTTGG
CCTCGAAACAGTAACTCAGTTAGTTTCATATTAAGGTCGGGTGTTAGTGCTTCTGTTAGA
AATAAAAGC
