# Supplementary material for: Antennal transcriptome analysis and expression profiles of odorant binding proteins in Eogystia hippophaecolus (Lepidoptera: Cossidae)
Source: BMC Genomics. 2016 Aug 18;17:651. doi: 10.1186/s12864-016-3008-4 (PMC4989532; doi:10.1186/s12864-016-3008-4)
Supplement: Additional file 3: — The protein names and gene accession numbers were used in phylogenetic trees. (PDF 182 kb) [file 12864_2016_3008_MOESM3_ESM.pdf]

Antennal transcriptome analysis and expression profile of odorant binding proteins in *Eogystia hippophaecolus* (Lepidoptera: Cossidae)

Ping Hu[a], Jing Tao[a], Mingming Cui[a], Chenglong Gao[a], Pengfei Lu[a], Youqing Luo[a]

Additional file 4

The protein names and gene accession numbers were used in phylogenetic trees

OR		OBP	
**Name**	**GI**	**Name**	**GI**
HarmORa	666916223	HarmOBPa	365919064
HarmORb	666916209	HarmOBPb	365919062
HarmORc	666916207	HarmOBP9	328879870
HarmORd	666916205	HarmOBP7	328879868
HarmORe	666916195	HarmOBP15	328879866
HarmORf	666916191	HarmOBP8	328879864
HarmORg	666916187	HarmOBP13	328879862
HarmORh	666916185	HarmOBP6	328879860
HarmORi	666916183	HarmOBP2	328879858
HarmORj	666916181	HarmOBP4	328879854
HarmORk	666916169	HarmOBP3	328879850
HarmORl	666916161	HarmOBP5	328879848
HarmORm	666916157	HarmOBP1	328879846
HarmORn	666916151	HarmGOBP1	15983753
HarmORo	666916147	HarmGOBP2	10129671
HarmORp	666916143	HarmPBP1	328879856
HarmORq	666916137	HarmPBP2	328879852
HarmORr	666916133	HarmPBP3	27464446
HarmORs	666916131		
HarmORt	666916129	**CSP**	
HarmORu	666916123	**Name**	**GI**
HarmORv	666916119	HarmCSPa	14091480
HarmORw	666916153	HarmCSPb	712120613

HarmOR17	486139589	HarmCSPc	712120595
HarmOR14b	486139562	HarmCSPd	712120580
HarmOR14a	486139541	HarmCSPe	712120564
HarmOR10	486139500	HarmCSPf	712120546
HarmOR9	486139482	HarmCSPg	712120529
HarmOR7	486139466	HarmCSPh	712120523
HarmOR6	486139449	HarmCSPi	712120519
HarmOR3	486139426	HarmCSP14	405117284
HarmOR83b	311901329	HarmCSP13	405117282
HarmOR12	194024962	HarmCSP12	405117280
HarmOR11	194024966	HarmCSP11	405117278
HarmOR3	194024958	HarmCSP10	405117276
DmelOR42a	22023972	HarmCSP9	405117274
DmelOR43b	22023986	HarmCSP8	405117272
DmelOR47b	45549172	BmorCSP1	112032026
DmelOR23a	28574001	BmorCSP2	112032057
DmelOR10a	17986023	BmorCSP3	145694413
DmelOR35a	24584456	BmorCSP4	112032114
DmelOR2a	17933522	BmorCSP5	112032133
DmelOR67b	24661763	BmorCSP6	112032153
DmelOR59c	17647785	BmorCSP7	112032179
DmelOR7a	17530847	BmorCSP8	112032195
DmelOR33c	17647767	BmorCSP9	112032214
DmelOR82a	24643875	BmorCSP10	112983056
DmelOR98a	24650735	BmorCSP11	112032244
DmelOR19a	24643445	BmorCSP13	112032283
DmelOR65a	24659319	BmorCSP14	112032298
DmelOR88a	24646756	BmorCSP15	112032318
DmelOR92a	24648414	BmorCSP16	112032338
DmelOR9a	17986019	DpleCSP11b	357621528

DmelOR56a	24655965	DpleCSP15	357621529
DmelOR49a	28573387	DpleCSP12	357621531
DmelOR85d	17986171	DpleCSP13	357621533
DmelOR45a	28573313	DpleCSP1	357626242
DmelOR74a	17986153	DpleCSP4	357626246
DmelOR22c	24580998	DpleCSP7	357626878
DmelOR1a	24638847	DmelCSPA98a	665395309
DmelOR30a	24582997	DmelCSPB42c	442622606
DmelOR13a	24642365	DmelCSPB93b	78711852
DmelOR94a	17738133	DmelCSPB93a	78709085
DmelOR24a	221472522	DmelCSPA75a	78707583
DmelOR83a	24644225	DmelCSPB38c	78707549
BmorOR18	290651022	DmelCSPA7a	78707537
BmorOR36	290650661	DmelCSPA56a	78707262
BmorOR16	290566751	DmelCSPA46a	78707184
BmorOR11	290563295	DmelCSPA86a	78706742
BmorOR54	290560855	DmelCSPA84a	78706694
BmorOR59	290560851	DmelCSPB74a	78706662
BmorOR14	290560836	DmelCSPB53b	62471727
BmorOR55	290559929	DmelCSPB53a	62471725
BmorOR57	261245111	DmelCSPB42a	45552485
BmorOR17	254939545	DmelCSPB42b	45552481
BmorOR8	254939543	DmelCSPB38a	45552427
BmorOR60	240255406	DmelCSPB38b	45552425
BmorOR9	182509188	DmelCSPA29a	28574075
BmorOR25	162462502	DmelCSPA87a	24646535
BmorOR37	162461284	DmelCSP2	48994226
BmorOR45	162461258	DmelCSP1	48994224
BmorOR33	158711751		
BmorOR35	158508574	**GR**	

BmorOR15	148298756
BmorOR30	148298744
BmorOR2	112983084
BmorOR4	112982926
BmorOR10	162462631
BmorOR7	163838688
BmorOR47	162462595
BmorOR21	162462571
BmorOR12	162462524
BmorOR34	158711753
BmorOR38	158508576
BmorOR41	148298822
BmorOR42	148298766
BmorOR19	148298665
BmorOR1	112983558
BmorOR6	112982988
BmorOR3	112982950
BmorOR5	112982948
BmorOR29	290650771
BmorOR27	290650676
BmorOR63	290563364
BmorOR56	290563360
BmorOR20	290563309
BmorOR61	290560861
BmorOR22	290560841
BmorOR13	290559921
BmorOR24	240255412
BmorOR46	240255410
BmorOR49	197322776
DmelOrco	24644231

**Name**	**GI**
DmelGR43a	47117920
DmelGR61a	17986119
DmelGR64a	24657115
DmelGR21a	118500892
DmelGR5a	24639922
DmelGR66a	281365837
DmelGR63a	221330835
DmelGR32a	45549158
DmelGR64f	45551511
DmelGR68a	24662881
DmelGR33a	45549284
DmelGR93a	24648814
DmelGR57a	17986103
DmelGR28a	45549155
DmelGR10a	24641287
DmelGR8a	28571153
DmelGR59f	28573623
DmelGR39b	24585658
DmelGR94a	24649189
DmelGR58c	28573606
DmelGR36a	45550994
DmelGR22a	24580943
DmelGR77a	24667642
DmelGR47b	45551079
DmelGR89a	45550757
DmelGR98d	24650640
DmelGR92a	45551934
DmelGR85a	24645557
DmelGR10b	17986021

AsegOrco	528523184
PexcOrco	747191675
PoctOrco	749692139
LdisOrco	558064779
ScerOrco	669192116
MsexOrco	940350663
CmedOrco	966344399
CpunOrco	451039884
AdisOrco	938453817
ObruPR1	749404788
MsexPR1	221329199
CmedPR4	966344409
cmedPR3	966344407
cmedPR2	966344405
cmedPR1	966344403
HarmPR14	752822706
PxylPR	768428803
OnubPR7b	768428803
OnubPR7a	732889446
OnubPR1	732889428
OnubPR3	732889402
OnubPR8	732889373
OnubPR5	732889350
OnubPR6	732889324
OnubPR6	732889294
MsexOR1	940350546
MsexOR4	940350551
MsexOR5	940350554
MsexOR 6	940350557
MsexOR 8	940350563

DmelGR9a	24640947
BmorGR68	350536295
BmorGR67	350536275
BmorGR60	195963349
BmorGR45	195963347
BmorGR9	195963345
BmorGR8	195963343
BmorGR10	912719236

**IR**	
**Name**	**GI**
HarmIRa	666916257
HarmIRb	666916255
HarmIRc	666916249
DmelIR25a	316994955
DmelIR10a	158031786
DmelIR52c	21645383
DmelIR56a	21626963
DmelIR67a	442631348
DmelIR41a	442622278
DmelIR84a	442617872
DmelIR11a	442616103
DmelIR7b	442615429
DmelIR75d	386771401
DmelIR94e	221458656
DmelIR54a	221330374
DmelIR52a	221330289
DmelIR7f	221329768
DmelIR21a	161076594
DmelIR62a	85725098

MsexOR 9	940350566
MsexOR 10	940350569
MsexOR 11	940350572
MsexOR 12	940350575
MsexOR 13	940350578
MsexOR 15	940350581
MsexOR 16	940350584
MsexOR 17	940350587
MsexOR 18	940350590
MsexOR 20	940350593
MsexOR 21	940350598
MsexOR 22	940350601
MsexOR 58	390276117
MsexOR 52	390276105
MsexOR 62	390276125
MsexOR 51	390276103
MsexOR 50	390276101
MsexOR OR-5	304423103
MsexOR4	304423101
MsexOR3	221329203
MsexOR2	221329201
MsexOR80	940350660
MsexOR65	940350656
MsexOR42	940350653
MsexOR40	940350648
MsexOR36	940350645
MsexOR33	940350635
MsexOR31	940350632
MsexOR30	940350629
MsexOR29	940350625

DmelIR100a	45550870
DmelIR60a	24762594
DmelIR68b	24663135
DmelIR56d	24655848
DmelIR51b	24653793
DmelIR48b	24652806
DmelIR47a	24652423
DmelIR85a	24645175
DmelIR20a	24643741
DmelIR7a	24640399
DmelIR76b	316994957
DmelIR8a	316994953
DmelIR64a	316994961
DmelIR75a	312434883
DmelIR93a	440217690
DmelIR92a	440217656
DmelIR87a	440217387

SNMP

**Name**	**GI**
TmolSNMP2	758213866
TmolSNMP1	758213864
DponSNMP2	471180467
DponSNMP1a	471180441
ItypSNMP1	459277281
ItypSNMP2	459277279
TcasSNMP1	189236600
TcasSNMP2	91092044
DmelSNMP1	440217709
DmelSNMP2	158516729

MsexOR27	940350622	HarmSNMP1	27462830
MsexOR26	940350619	BmorSNMP1	112984488
MsexOR25	940350616	BmorSNMP2	827550980
MsexOR24	940350607	AmelSNMP2	374253753
MsexOR23	940350604	AmelSNMP1	384872681
