# Supplementary material for: Antennal transcriptome analysis and expression profiles of odorant binding proteins in Eogystia hippophaecolus (Lepidoptera: Cossidae)
Source: BMC Genomics. 2016 Aug 18;17:651. doi: 10.1186/s12864-016-3008-4 (PMC4989532; doi:10.1186/s12864-016-3008-4)
Supplement: Additional file 4: — Primers were designed for fluorescence quantitative real-time PCR. (PDF 90 kb) [file 12864_2016_3008_MOESM4_ESM.pdf]

Antennal transcriptome analysis and expression profile of odorant binding proteins in *Eogystia hippophaecolus* (Lepidoptera: Cossidae)

Ping Hu[a], Jing Tao[a] , Mingming Cui[a], Chenglong Gao[a], Pengfei Lu[a] , Youqing Luo[a]

Additional file 3

Primer used for fluorescence quantitative real-time PCR

Name	Forward primer	Reverse primer
OBP1	CAATGCAAAGTACCGAAGCA	CTGGAAGACACACGCAAAGA
OBP2	GCAGATGGTCGTGGTAGCAG	CACCAGCTAGCGCTTCATTG
OBP4	CCATGTCTTCGTGTGTGGAC	CAGAGACGAGATCGGAAAGG
OBP5	CAAGTGGGCAACATAGAGCA	TGGCGGAAACATCATATCAA
OBP6	TATTCAGGAAAGCGGGATGG	TGTAGACACCTTGGGCTGGA
OBP8	CATGGCACGTTCACAACTCA	AGACGGCGGCAATTTAAGAA
OBP10	TTACGCGCCTGTTCTTATCC	CATGCCCATACTGATTCGTG
Actin	CGACTTCGAACAGGAGATGG	TCGTCTCATGAATGCCACAG
GOBP1	CAGAGGAGCAGATGGAGGAG	TGGGGAAGGACTTGATGAAC
GOBP2	AAGCACTTCTGGAGCGAAGA	CTTCTCGCAATTGTGGATCA
