# Supplementary material for: Antennal transcriptome analysis and expression profiles of odorant binding proteins in Eogystia hippophaecolus (Lepidoptera: Cossidae)
Source: BMC Genomics. 2016 Aug 18;17:651. doi: 10.1186/s12864-016-3008-4 (PMC4989532; doi:10.1186/s12864-016-3008-4)
Supplement: Additional file 5: — Reference gene and statistics analysis of CQ in fluorescence quantitative real-time PCR. Table S1. Normfinder result of four reference genes. Figure S1. M value of gNorm result of four reference genes. Figure S2. Normal distribution tests results of nine OBPs. Figure S3. Normal Q-Q plot results of nine OBPs. Figure S4. Equal variances test results of nine OBPs. (PDF 501 kb) [file 12864_2016_3008_MOESM5_ESM.pdf]

Antennal transcriptome analysis and expression profiles of odorant binding proteins in *Eogystia hippophaecolus* (Lepidoptera: Cossidae)

Ping Hu[a], Jing Tao[a] , Mingming Cui[a], Chenglong Gao[a], Pengfei Lu[a], Youqing Luo[a]

Additional file 5

Reference gene and statistics analysis of CQ in fluorescence quantitative real-time PCR

Table S1 Normfinder result of four reference genes

Gene name	Stability value
A1-2	0.197
A2-1	0.017
T1-1	0.039
T1-2	0.029

Best gene:A2-1

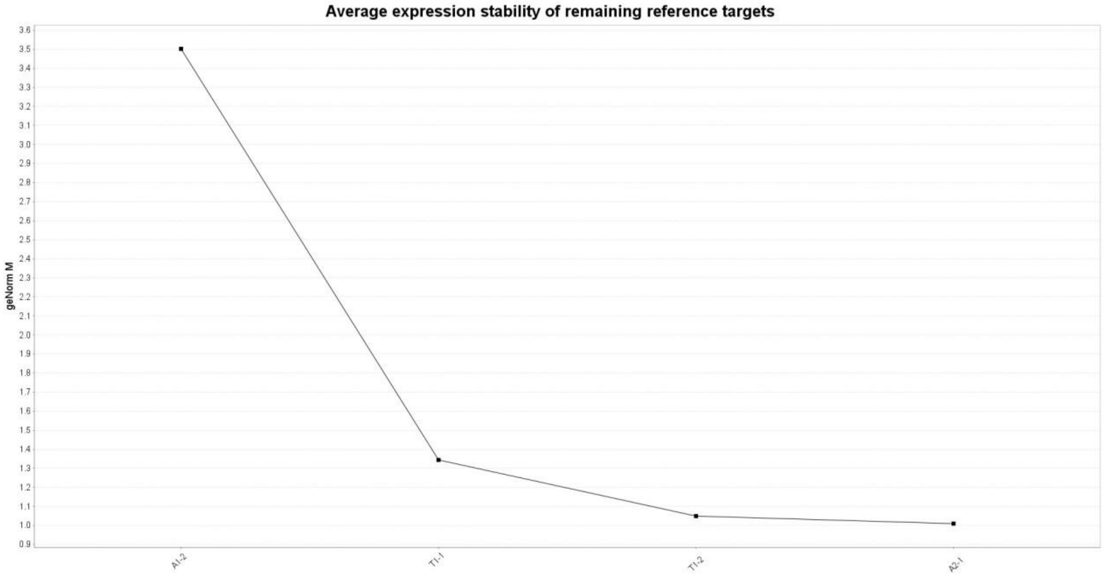

Figure S1 M value of gNorm result of four reference genes

One-Sample Kolmogorov-Smirnov Test of OBP1

		CQ
N		15
Normal Parameters[a,b]	Mean	.0021
	Std. Deviation	.22537
Most Extreme Differences	Absolute	.137
	Positive	.100
	Negative	-.137
Kolmogorov-Smirnov Z		.529
Asymp. Sig. (2-tailed)		.942

a. Test distribution is Normal.
b. Calculated from data.

One-Sample Kolmogorov-Smirnov Test of OBP2

		CQ
N		14
Normal Parameters[a,b]	Mean	-.01929
	Std. Deviation	.500197
Most Extreme Differences	Absolute	.190
	Positive	.111
	Negative	-.190
Kolmogorov-Smirnov Z		.709
Asymp. Sig. (2-tailed)		.696

a. Test distribution is Normal.
b. Calculated from data.

One-Sample Kolmogorov-Smirnov Test of OBP4

		CQ
N		14
Normal Parameters[a,b]	Mean	.2838
	Std. Deviation	.38957
Most Extreme Differences	Absolute	.289
	Positive	.289
	Negative	-.210
Kolmogorov-Smirnov Z		1.081
Asymp. Sig. (2-tailed)		.193

a. Test distribution is Normal.
b. Calculated from data.

One-Sample Kolmogorov-Smirnov Test of OBP5

		CQ
N		16
Normal Parameters[a,b]	Mean	.71608
	Std. Deviation	1.168728
Most Extreme Differences	Absolute	.258
	Positive	.258
	Negative	-.179
Kolmogorov-Smirnov Z		1.033
Asymp. Sig. (2-tailed)		.237

a. Test distribution is Normal.
b. Calculated from data.

One-Sample Kolmogorov-Smirnov Test of OBP6

		CQ
N		16
Normal Parameters[a,b]	Mean	.25818
	Std. Deviation	1.060942
Most Extreme Differences	Absolute	.241
	Positive	.241
	Negative	-.166
Kolmogorov-Smirnov Z		.965
Asymp. Sig. (2-tailed)		.310

a. Test distribution is Normal.
b. Calculated from data.

One-Sample Kolmogorov-Smirnov Test of OBP8

		CQ
N		15
Normal Parameters[a,b]	Mean	-.2067
	Std. Deviation	.94499
Most Extreme Differences	Absolute	.165
	Positive	.165
	Negative	-.163
Kolmogorov-Smirnov Z		.639
Asymp. Sig. (2-tailed)		.809

a. Test distribution is Normal.
b. Calculated from data.

One-Sample Kolmogorov-Smirnov Test of OBP10

		CQ
N		16
Normal Parameters[a,b]	Mean	-.7251
	Std. Deviation	.79784
Most Extreme Differences	Absolute	.280
	Positive	.261
	Negative	-.280
Kolmogorov-Smirnov Z		1.121
Asymp. Sig. (2-tailed)		.162

a. Test distribution is Normal.
b. Calculated from data.

One-Sample Kolmogorov-Smirnov Test of GOBP1

		CQ
N		15
Normal Parameters[a,b]	Mean	.88733
	Std. Deviation	0.662253
Most Extreme Differences	Absolute	.398
	Positive	.398
	Negative	-.229
Kolmogorov-Smirnov Z		1.540
Asymp. Sig. (2-tailed)		.067

a. Test distribution is Normal.
b. Calculated from data.

One-Sample Kolmogorov-Smirnov Test of OBP9

		CQ
N		14
Normal Parameters[a,b]	Mean	.20149
	Std. Deviation	1.526434
Most Extreme Differences	Absolute	.213
	Positive	.213
	Negative	-.203
Kolmogorov-Smirnov Z		.798
Asymp. Sig. (2-tailed)		.547

a. Test distribution is Normal.
b. Calculated from data.

FigureS2 Normal distribution tests results of nine OBPs

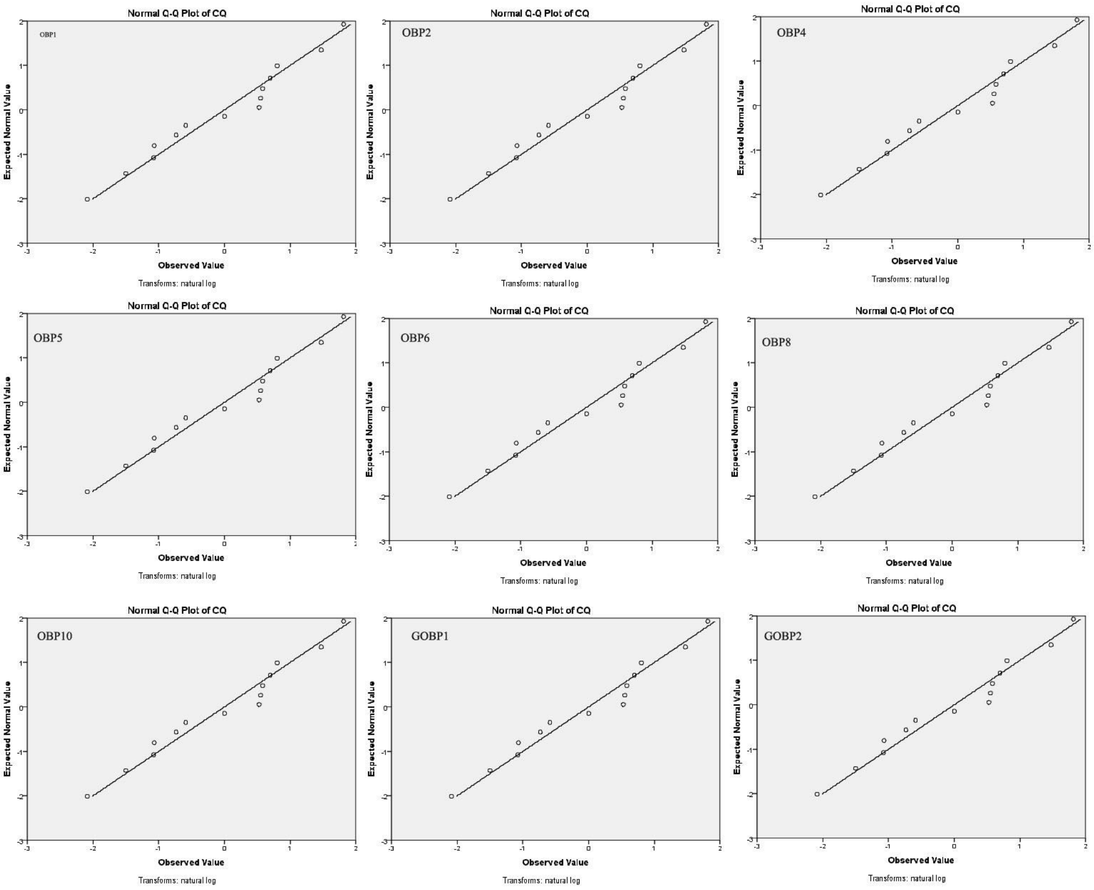

FigureS3 Normal Q-Q plot results of nine OBPs

Homogeneity test of variance of OBP1

Levene Statistic	df1	df2	significance
1.142	3	11	.375

Homogeneity test of variance of OBP2

Levene Statistic	df1	df2	significance
1.826	3	10	.206

Homogeneity test of variance of OBP4

Levene Statistic	df1	df2	significance
2.669	3	10	.105

Homogeneity test of variance of OBP5

Levene Statistic	df1	df2	significance
2.751	3	12	.089

Homogeneity test of variance of OBP6

Levene Statistic	df1	df2	significance
2.094	3	12	.154

Homogeneity test of variance of OBP8

Levene Statistic	df1	df2	significance
1.585	3	11	.249

Homogeneity test of variance of OBP10

Levene Statistic	df1	df2	significance
1.127	3	12	.377

Homogeneity test of variance of GOBP1

Levene Statistic	df1	df2	significance
.744	3	11	.548

Homogeneity test of variance of GOBP2

Levene Statistic	df1	df2	significance
2.592	3	10	.111

Figure S4 Equal variances test results of nine OBPs
